
JOURNAL INFORMATION
==============================
NLM Title Abbreviation: J Child Orthop
Journal ID: J Child Orthop
NLM Journal ID: 101313582

Journal of Children's Orthopaedics

ISSN: 1863-2521
EISSN: 1863-2548
Publisher: SAGE Publications

ARTICLE INFORMATION
==============================
PMCID: PMC3955428
PMID: 24634255
DOI: 10.1007/s11832-014-0575-2
Article ID: 575
Article version: 1
Subjects: Abstracts

EPOS 33rd Annual Meeting

Electronic publication date: 2014 Mar 15
Print publication date: 2014 Apr
Volume: 8
Issue: Suppl 1
First page: 67
Last page: 104
Copyright: © The Author(s) 2014
License: This article is published under license to BioMed Central Ltd. Open Access This article is distributed under the terms of the Creative Commons Attribution License which permits any use, distribution and reproduction in any medium, provided the original author(s) and source are credited.
License URL: https://creativecommons.org/licenses/by/4.0/

issue-copyright-statement: © The Author(s) 2014

==============================
J Child Orthop (2014) 8 (Suppl 1):S67–S104
DOI 10.1007/s11832-014-0575-2

ABSTRACTS

### EPOS 33rd Annual Meeting

© The Author(s) 2014. The abstracts are published with open access at Springerlink.com

### EP1
### Experimental Study of Progressive Tibial Lengthening in Dogs with Ilizarov Technique. Comparison With and Without Associated Intramedullary K Wires

**Dmitry Popkov**[1], Arnold Popkov[1], Natalia Kononovich[1], Dominique Barbier[2], Pierre Journeau[2], Dimitri Ceroni[3], Pierre Lascombes[3]

1. Russian Ilizarov Scientific Center for Restorative Traumatology and Orthopaedics, Kurgan, Russia; 2. Service d'orthopédie pédiatrique, Hôpital d'Enfants, CHU de Nancy, Nancy, France, Metropolitan; 3. Service d'orthopédie pédiatrique, Département de l'enfant et l'adolescent, Hôpitaux universitaires de Genève, Genève, Switzerland.

**LEVEL 2/Basic science**
Keywords: Limb lengthening, Flexible intramedullary nailing.

***Purpose*** The delay of union of the bone regenerate in lengthening surgical procedures and the healing index (HI) are major factors in the quality of the results in progressive bone lengthening. Early removal of an external fixator is associated with a lower rate of postoperative complications including pin track infections, and with a better muscle and joint function recovery. The addition of intramedullary wires (IMW) to the external fixator led to a 9 to 49 % HI decrease depending on the series. The clinical based hypothesis was that IMW may accelerate the formation and the quality of the bone regenerate.
***Methods*** A progressive tibial lengthening of 28 mm was performed in 12 dogs operated on using the classical Ilizarov technique (Group I), and in 12 dogs operated on using the same technique but with the addition of two IMW of 1.5 mm diameter (Group II). The following criteria were assessed: HI, post operative complications, weekly X-ray aspect of the bone regenerate during the lengthening and fixation periods, and the histological aspect at sacrifice.
***Results*** The HI was 32.1 % shorter (mean value) in Group II compared with Group I. No clinical complication was observed in either Group. The radiological criteria of bone union were observed at d15 of the fixation period in Group II versus d30 in Group I. The histology showed that maturation happened earlier and bone cortices were thicker in Group II versus Group I. An endo medullary ossification was present along the IMW in Group II, whereas it was absent in Group I. The presence of the IMW contributes definitively to a stimulation of the ossification processes of the bone regenerate, and to the acceleration of the bone union. The IMW allowed an earlier removal of the external fixator: a 32 % reduction in time compared with cases without IMW. In addition, the new endo medullary bone formation and the presence of IMW are expected to increase the mechanical resistance of the bone regenerate.
***Conclusions*** Improvement of quantitative and qualitative criteria of bone regenerate in progressive bone lengthening with external fixator combined with IMW was demonstrated in this experimental study.
***Significance*** These favourable results encourage the authors to propose IMW in addition to the external fixator in patients treated by bone lengthening.

### EP2
### Is Thermal RFA Epiphysiodesis a Safe Procedure for the Articular Cartilage?

**Juan Manuel Shiguetomi-Medina**, Ahmed Abdul-Hussein Abood, Ole Rahbek, Hans Stødkilde-Jørgensen, Bjarne Møller-Madsen

Aarhus University Hospital, Aarhus, Denmark

**LEVEL 1/Basic science**
Keywords: Epiphysiodesis, RFA, MRI, Cartilage damage.

***Purpose*** Proof of concept that epiphysiodesis made with RFA is a safe procedure that disrupts the growth plate without damaging the adjacent joint articular cartilage.
***Methods*** Epiphysiodesis using RFA (92–98 °C) was done in vivo for 8 min in the tibial physis of 40 growing (skeletally immature) pigs. In addition, three tibiae were ablated for 16 min (double time), and three more for 24 min (triple time). As a cartilage-damage reference, 6 tibiae were ablated on the joint articular cartilage for 8 min. After the procedure, the animals underwent euthanasia and all the tibiae were harvested. On each tibia, MRI was done ex vivo after the procedure to evaluate the joint articular cartilage in all samples using T1-weighted, T2-weighted and water content sequences under a 1.5 T magnetic field. Morphology, intensity, and calculated water content (from T1-map values) in cartilage were studied.
***Results*** On the intentionally damaged articular cartilage, intensity changes were observed on the MR images. These images were used as a reference for damage. We found no evidence of articular cartilage damage on the 40 RFA procedures. The tibiae ablated for 16 min showed a lesion on the physis but the articular joint cartilage was intact. No articular cartilage damage was found on the tibiae ablated for 16 min. On the burned cartilage group, based on T1-map values, a mean water content increase of 30 % (20–42 %, SD = 8 %) was calculated in the articular cartilage. Water content of the articular cartilage was not affected on any of the ablated tibiae. Overall, a mean difference of 0.72 % (0.31–1.08 %, SD = 0.72 %).
***Conclusions*** Epiphysiodesis using RFA is safe for the adjacent articular joint cartilage. Structural bone characteristics of pig bone resemble those of human bone. This study simulates possible results of RFA epiphysiodesis in humans. Previous studies suggest that an 8 min ablation is enough to disrupt the growth plate. This study shows that RFA can be done safely in the growing physis of pigs even with triple-long procedures.
***Significance*** RFA offers an alternative to do epiphysiodesis. This study probes it is a safe procedure to the articular cartilage and shows the possibility of a follow up based on specialized MRI.

### EP3
### Fracture Tolerance Related to Skeletal Development: 3-Point Bending of Human Femurs

**Julio Duart**[1], Jason Forman[2], Eduardo Del-Pozo[3], Myriam Brun[1], Serafin Garcia- Mata[1], Ioannis Symeonidis[4], Maria Segui-Gomez[3]

1. Navarra Hospital Complex, Pamplona, Spain.; 2. University of Virginia Center for Applied Biomechanics, Virginia, USA; 3. European Center for Injury Prevention, Pamplona, Spain; 4. Institute for Legal Medicine-University of Munich, Munich, Germany.

**LEVEL 1/Basic science**
Keywords: Femur, Fracture, Tolerance, Development, Biomechanics.

***Purpose*** To investigate the roles of geometric and material factors in the changes that occur in long-bone fracture tolerance throughout skeletal development.
***Methods*** We have performed a dynamic 3-point bending test of human femora. The specimens were prepared by first removing all of the superficial tissue and muscular attachments. Then, the proximal and distal ends of the specimens were encased in blocks of a polymer material. The specimens were oriented such that the bending moment would be applied about the anterior-posterior axis, with load applied to the mid-shaft of the femur in a medial direction. The geometry of each specimen was documented after plotting via a clinical computed tomography scan. A bending moment was then applied via a loading ram engaging the specimen at the mid-shaft, with a target velocity of 1.5 m/s. An analytical model was developed to decompose the observed changes with age into contributing geometric and material factors. We have considered an analytical model of mid-shaft 3-point bending of a simplified beam. We have studied the relationship between fracture moment and age, fracture moment and geometric factors, material factors and age.
***Results*** Ten tests were performed with specimen ages ranging from 1.3 to 20 years. The fracture moment ranged from 61 to 533 Nm. The entire dataset tended to show a relation between fracture moment and age as an upward curve, rising steeply through adolescence. Consistent with the beam equation, the fracture moment followed a general linear trend with the cross-sectional geometry parameter. This relationship did not diverge for increasing cross-sectional geometry, suggesting the absence of change in the material characteristics (or other factors) affecting the failure moment.
***Conclusions*** The analysis suggests that the increase in fracture moment during development was primarily attributable to changes in the cross-sectional bone geometry during growth.
***Significance*** This is the first study (to the authors' knowledge) to quantitatively relate changes in long-bone fracture tolerance with age to contributing mechanistic factors. The descriptive models developed here provide an understanding of the relationship between those geometric factors, remaining factors, and fracture moment with age. These component-level models may facilitate improved prediction of femur fracture risk.

### EP4
### First Experience with Molecular Analysis of Pseudoarthrosis Tissue in PFFD

**Monika Frydrychova**, Jirí Chomiak, Pavel Dungl, Michal Burian, Daniel TvrdíK

Orthop. Dept. 1st Medical Faculty Charles University and Hospital Na Bulovce, Prague, Czech Republic.

**LEVEL 3/Basic science**
Keywords: Proximal femoral focal deficiency, Gene expression, Microarray analysis, Angiogenesis, Osteogenesis.

***Purpose*** Although the congenital short femur is morphologically well characterized, changes at molecular level have not been described in the literature to date. The absence of such information together with unknown etiology of the defect was the reason why the authors chose to analyze angiogenesis and osteogenesis in the pseudoarthrosis tissue in PFFD patients compared to physiological bone. The authors had expected differences in gene expression, especially in the quantity of expressed genes.
***Methods*** A piece of bones was removed during an elective surgery procedure, placed in a RNA stabilization reagent, which prevents RNA degradation, and deep frozen. Thereafter, RNA was isolated and profile of transcription was analyzed by biochip analysis (SuperArray Bioscience Corporation). It is possible to detect 113 genes of osteogenesis and angiogenesis. During the study period from the end of 2005 till the end of 2008, 7 samples from patients with PFFD and 3 physiological bone samples were examined. Several analyses were repeated to confirm the results; altogether 13 chips for osteogenesis and 11 for angiogenesis were used.
***Results*** The differences in quantity and representation of the genes were noted. Some genes were considered as over-expressed in PFFD tissue vs. control (e.g. the gene for calcitonin receptor, collagen XII, collagen I alpha 2, collagen II, collagen IX, FGFR2, fibronectin, integrin) and other genes as under-expressed (e.g. the gene for annexin A5, collagen XVIII alpha 1, collagen I alpha I, cathepsin K, FGFR1, FGFR3, IGF2, VEGFB).
***Conclusions*** The differences in gene expression confirmed the authors' hypothesis. So far, the results cannot be generalized: this is the first step for follow-up experiments to confirm the suggested information and to integrate it with clinical findings, such as the alternative blood supply of the affected extremity in some patients.
***Significance*** The study is a pilot project in this field, without any possibility of comparison but which may stimulate further research.

### EP5

### Effect of Botulinum Neurotoxin A Injection on Neuromuscular Transmission of High Frequency Stimulation in Juvenile Rat Skeletal Muscle

**Sofie Gjessing**[1], Ole Rahbek[1], Ole B. Nielsen[2], Bjarne Moeller-Madsen[1]

Department of Children's Orthopaedics, Aarhus University Hospital, Aarhus, Denmark; 2. Department of Biomedicine, Aarhus University, Aarhus, Denmark

**LEVEL 2/Basic science**

Keywords: Cerebral palsy, Botulinum toxin A, Spasticity, Motor programs, Neuromuscular transmission.

***Purpose*** Intramuscular injection of botulinum neurotoxin A (BoNT-A) is a widespread treatment of focal spasticity in children with cerebral palsy (GMFCS levels I-III). Often the aim is to improve gait pattern by reducing hypertonicity while preserving motor function. Walking and other dynamic movements require fast generation of force early in contractions, which often is achieved by initiating trains of activating action potentials in the motor nerves by two (or three) closely spaced pulses. In humans, the interpulse interval in such doublets may be as short as 2.5 ms, approaching the maximal transmission rate of the neuromuscular junction. Here, we hypothesized that BoNT-A reduces the ability of the neuromuscular junction to transmit doublets and, therefore, also reduces their potentiating effect on force development.
***Methods*** Juvenile Wistar rats received under anaesthesia (hypnorm/midazolam) injection with BoNT-A (6 U/kg bw) into the gastrocnemius muscle leaving the contralateral leg as a control. Two animals were sacrificed every 24 h for 96 h, and isolated soleus muscles were incubated in KR-buffer and stimulated electrically with single pulses or doublets with interpulse interval from 2 to 10 ms, corresponding to high frequency stimulation from 100 to 500 Hz, while isometric force was measured. Muscle fibers were stimulated directly or via the nerve using pulses of 0.2 and 0.02 ms duration, respectively.
***Results*** Preliminary results from 8 rats showed that BoNT-A caused a progressive decline in nerve stimulated force in the treated leg, and after 96 h, force was reduced by $86.0 \pm 9.4$ % ($n = 2$). In control muscles, doublet stimulation increased single twitch force by up to 120 %. This potentiation was, however, attenuated by BoNT-A in a manner that depended on the reduction in nerve stimulated force and the interpulse interval. Thus, in muscles where BoNT-A caused a 50–75 % reduction of maximal nerve stimulated force, potentiation of twitch force by 2 ms doublets was reduced by $\sim$55 % whereas longer doublets produced normal potentiation. In muscles where maximal force was reduced by more than 75 %, the potentiation by 4 ms doublets was in addition reduced by $\sim$20 %.
***Conclusions*** Intramuscular injections of BoNT-A caused a reduction in the ability of the neuromuscular junction to transmit doublets that depended on the remaining nerve stimulated force in juvenile rat muscles.
***Significance*** The results suggest that BoNT-A can interfere with motor function during dynamic contraction, which, depending on the dose used, could compromise gait in children treated with the compound.

### EP6

### Effect of Botulinum Toxin Type A on the Treatment of Muscle Contractures in Lower Limbs of Children With Spastic Type Cerebral Palsy

Hakan Omeroglu

Eskisehir Osmangazi University Hospital, Eskisehir, Turkey

**LEVEL 4/Cerebral palsy**

Keywords: Cerebral palsy; Botulinum toxin type A.

***Purpose*** The aim of this case series was to evaluate the effect of Botulinum Toxin type A (BTXA) application in the treatment of lower limb muscle contractures in children who had spastic type cerebral palsy (CP).
***Methods*** This study included 91 children (44 girls, 47 boys) with spastic type (CP) and walking ability with or without supports. All BTXA (BOTOX, Allergan) applications were performed under mask anaesthesia using the palpation method by the same surgeon. A cast in the corrected position was applied for 10–14 days followed by physiotherapy and use of the usual orthoses. While seventy children had one session of BTX-A application, the rest had repeated applications (19 had injections twice, one had three and one had four injections). The mean age for the 115 sessions was 6 (2.5–15) years. Pre BTXA and maximum corrected post BTXA contracture values were recorded. Thomas test was used for the assessment of the iliopsoas contracture, Holt test for the hamstring group contracture and Ely test for the rectus femoris contracture. Adductor group contracture was assessed by measuring the amount of maximum hip abduction while the knees were fully extended. Gastrosoleus contracture was evaluated by measuring the amount of maximum ankle dorsiflexion with the knees fully extended. A minimum 3 months complete follow-up was obtained to assess the maximum correction of the contracture following BTXA applications.
***Results*** 20 iliopsoas (mean dose 1.3 μ/kg), 53 adductor longus (mean dose 1.3 μ/kg), 105 medial hamstring (mean dose 3.1 μ/kg), 7 rectus femoris (mean dose 1.3 μ/kg) and 168 gastrocnemius (mean dose 4.4 μ/kg) applications were performed. Mean total dose per patient was 10.2 (3.5–20) μ/kg. A significant correction was obtained in all but rectus femoris contractures within the first 3 months following BTXA application ($P < 0.001$). Prone Ely test did not improve in 5 of 7 patients.
***Conclusions*** With the applied doses available, BTXA application under mask anesthesia and followed by short-term cast application, physiotherapy and orthosis use is efficient to treat the iliopsoas, adductor, medial hamstring and gastrocnemius contractures of the children with spastic type CP.
***Significance*** The BTXA dose for rectus femoris in this study (about 1.5 μ/kg) appears insufficient for overcoming the rectus femoris contracture and a higher dose may be needed to overcome this contracture in children.

### EP7

### Are Clinical Measurements Linked to the Gait Deviation Index in Cerebral Palsy Patients?

**Yoshimasa Sagawa**[1], Geraldo De Coulon[2], Pierre Lascombes[2], Stephane Armand[2]

UMR CNRS, Valenciennes, France; 2. Hopital universitair Geneve, Geneve, Switzerland

**LEVEL 4/Cerebral palsy**
Keywords:

***Purpose*** From a dataset of clinical assessments and gait analysis, this study was designed to determine which of the assessments or their combinations would most influence a low gait index (i.e., severe gait deviations) for individuals with cerebral palsy.
***Methods*** A retrospective search, including clinical and gait assessments, was conducted from August 2005 to September 2009. Population: 155 individuals with a clinical diagnosis of cerebral palsy (CP) [mean age (SD) 11 (5.3) years] were selected for the study. Quinlan's Interactive Dichotomizer 3 algorithm for decision-tree induction, adapted to fuzzy data coding, was employed to predict a Gait Deviation Index (GDI) from a dataset of clinical assessments (i.e., range of motion, muscle strength, and level of spasticity).
***Results*** Seven rules that could explain severe gait deviation (a fuzzy GDI low class) were deduced.
Overall, the fuzzy decision-tree method was highly accurate and permitted us to correctly classify GDI classes 9 out of 10 times using our clinical assessments.
***Conclusions*** There is an important relationship between clinical parameters and gait analysis. We have identified the main clinical parameters and combinations of these parameters that lead to severe gait deviations. The strength of the hip extensors, the level of spasticity and the strength of the tibialis posterior were the most important clinical parameters for predicting a severe gait deviation.
***Significance*** The strength of hip extensors and tibialis posterior should be preserved.

### EP8

### How Do Semitendinosus and Gastrocnemius Tenotomies Affect the Popliteal Angle in Children With Cerebral Palsy?

**Ilker Abdullah Sarikaya**[1], Muharrem Inan[2]

Mus State Hospital, Mus, Turkey; 2. Department of Orthopaedics, Cerrahpasa Faculty of Medicine, Istanbul, Turkey

**LEVEL 3/Cerebral palsy**
Keywords: Popliteal Angle, Semitendinosus Tenotomy, Gastrocnemius Tenotomy, Cerebral Palsy.

***Purpose*** Flexion contracture secondary to hamstring and gastrocnemius muscle spasticity is the most common knee problem in children with cerebral palsy (CP). Semitendinosus tenotomy is commonly performed to eliminate knee flexion contracture and improve gait as a part of the hamstring lengthening procedure. Triceps surae affects knee kinetics and may require surgical lengthening in CP. The purpose of this study was to determine the efficacy of semitendinosus and gastrocnemius tenotomies on popliteal angle in children presenting with a knee flexion contracture.
***Methods*** Of the 44 patients, 19 were females and 25 males. Mean age at surgery was 8.1 years (range 4–14). There were 50 semitendinosus and 28 gastrocnemius tenotomies in this prospective study. Subjects were included if they had a diagnosis of CP and needed semitendinosus and/or gastrocnemius tenotomies. Subjects were not included if they had previously undergone any open hamstring lengthening or gastrocnemius tenotomy on the same side. The popliteal angle was measured under general anaesthesia with the patient in supine position, the hip in 90° flexion and the contralateral limb in extension before and after the tenotomy. A separate grouping of patients older and younger than seven years of age at time of surgery was made.
***Results*** Mean popliteal angles were 47.7° before semitendinosus tenotomy and 50.9° before gastrocnemius tenotomy. Popliteal angle decreased to 14.5° and 6.0° following the semitendinosus and gastrocnemius tenotomies respectively. The improvement of the popliteal angle was 34 % in patients younger than the age of seven and 28 % older than age of seven who were managed with semitendinosus tenotomy.
***Conclusions*** The results of this study indicate 30.8 % increase in knee flexion with semitendinosus tenotomy and 11.3 % with gastrocnemius tenotomy in children with CP. These findings provide important data for surgical planning for knee flexion contracture in CP.
***Significance*** These findings provide important data for surgical planning for knee flexion contracture in CP.

### EP9

### Whether the Hamstring Muscles Should be Preserved or not for Treatment of Hip Subluxation of Cerebral Palsy With Matsuo's Muscle Release Procedure

**Mayuki Taketa**, Yutaka Oketani, Akifusa Wada, Hideaki Kubota, Toshio Fujii

Saga Handicapped Children's Hospital, Saga-Shi, Japan

**LEVEL 3/Cerebral palsy**
Keywords: Cerebral Palsy, Hip Subluxation, Surgery.

***Purpose*** The concept of Matsuo's procedure is release of both of antagonistic polyarticular muscles while preserving monoarticular muscles as much as possible. Matsuo's procedure for the hip consists of lengthening or section of psoas, rectus femoris, adductor longus, adductor magnus, gracilis and hamstring muscles. We have performed Matsuo's procedure for treatment of cerebral palsy (CP) since 2003. We have preserved hamstring muscles (HM) from 2003 to 2008: this preserving procedure was tendon lengthening of proximal semimembranosus, semimembranosus and biceps femoris. Since 2008, we totally sectioned HM for treatment of subluxation of the hips of children classified Gross Motor Function Classification System (GMFCS) level 4 or 5. The purpose of this study was to evaluate the effect of section of HM for treatment of dislocation of the hip in patients of GMFCS level 4 and 5.
***Methods*** We had surveyed children with CP who were performed Matsuo's procedure for their hip subluxation in Saga Children's Hospital from 2003 to 2011. The candidates were classified with the Gross Motor Function Classification System (GMFCS) level 4 or 5 before surgery. We excluded patients with matured pelvis at surgery and/or within one year after surgery at the survey. Thirty-eight hips in 26 patients (15 boys and 11 girls) were included. HM preserved group (Group 1) included 26 hips and HM sectioned group (Group 2) included 12 hips. We examined retrospectively the radiographic images by Migration Percentage (MP %) to evaluate subluxation of the hip.

***Results*** The average age at surgery was 5.2 years of age in Group 1 and 5.4 years in Group 2. Observation period after surgery was 5.5 years on average in Group 1 and 2.1 years in Group 2. MP improved from 57.1 to 36.0 % on average ($p < 0.01$) in Group 1 and from 56.7 to 28.8 % in Group 2. MP after surgery were not different between Group 1 and 2.
***Conclusions*** We proposed that Matsuo's procedure was useful for treatment of subluxation of the hip in CP. To treat subluxation of the hip, it is not necessary to preserve HM with Matsuo's procedure.
***Significance*** Our findings are helpful for decision making of surgical procedures for subluxation of the hip in CP.

### EP10
### Daily Life Benefit of Wrist Arthrodesis in Severely Involved Cerebral Palsy Patients

**Elke Viehweger**[1], Jean-Camille Mattei[2], Elisabeth Castanier[2], Marie-Ange Rohon[3], Christophe Boulay[1], Jean-Luc Jouve[4]

1. Timone Children's Hospital, Pediatric Orthopaedics, Motion Analysis Center, Aix-Marseille University, Marseille, France; 2. Timone Children's Hospital, Pediatric Orthopaedics, Marseille, France; 3 Rehabilitation Institute Pomponiana-Olbia, Hyeres, France; 4. Timone Children's Hospital, Pediatric Orthopaedics, Aix-Marseille University, Marseille, France

**LEVEL 4/Cerebral palsy**
Keywords: Wrist, Arthrodesis, Cerebral Palsy, Daily Life, Outcome.

***Purpose*** Wrist flexion contracture is a major component of disability in children with severe neurological involvement: appearance, hygiene and function are affected significantly but less considered in the global treatment strategy than lower limb or spine surgery. The efficacy of wrist arthrodesis regarding functional outcome and satisfaction was evaluated.
***Methods*** 14 patients (18 wrists), 5 girls and 9 boys (mean age: 17.3 years; min: 12, max: 23) were treated by wrist arthrodesis. Demographic and surgical data and complication rate were collected. The preoperative wrist resting position and follow-up wrist fusion position were noted. All patients were reviewed by a neutral reviewer using pre and post operative House Classification of upper extremity functional use, Manual Ability Classification System (MACS) and grasp/release possibility. Caregiver based questionnaires were proposed separate from the final follow-up visit: the Disability Assessment Scale (DAS) which assessed personal hygiene, daily activities, limb position and pain and a Visual Analog Scale (VAS) to evaluate appearance, function, daily cares, hygiene and pain. Statistical tests were processed to compare pre and post surgery House, DAS, VAS and GRASP possibility. Significance level was $p < 0.05$.
***Results*** Preoperative mean MACS was $4.8 \pm 0.6$. The House scale showed an average score of 0.65 which signifies that the patient does not use his/her limb. DAS improved statistically and clinically significantly from 6.1 to a 2.2 average post operatively which meant that major discomforts were globally less troublesome than before surgery. With the subjective VAS, 94 % of the caregivers reported improvement in appearance and function and global satisfaction, 76 % in daily care and 70 % in hygiene.
***Conclusions*** Wrist arthrodesis is a reliable technique which may improve patients' disability, and caregivers' satisfaction.
***Significance*** Upper limb deformities have to be part of the treatment strategy to improve daily life in neurologically severely involved patients.

### EP11
### Soft-Tissue Release as Prevention and Treatment of Lateral Migration of the Femoral Head in Children With Spastic Cerebral Palsy

Karel Urbasek

University Hospital Brno, Brno, Czech Republic

**LEVEL 3/Cerebral palsy**
Keywords: Cerebral Palsy, Soft Tissue Release, Hip Subluxation, Migration Percentage.

***Purpose*** Subluxation of the hip joint is one of the most frequent complications in children with spastic forms of juvenile cerebral palsy. Without treatment, lateral migration of the femoral head increases on average by 7.7 % per year and can progress, in association with acetabular dysplasia, to hip dislocation. The risk of progression to hip dislocation is 15–20 % in the total population of children with juvenile cerebral palsy, and it varies considerably among the individual types from 0 % in simple ataxia up to 79 % for spastic tetraplegia. This study evaluated the advantage of soft-tissue release of spastic hip adductors and flexors to prevent lateral migration of the femoral head.
***Methods*** 72 spastic cerebral palsy patients were included (27 girls and 45 boys) (27 diparesis and 44 quadriparesis) who underwent open adductor and rectus femoris tenotomy and iliopsoas tendon recession for either hip subluxation or for severe adduction contracture. Criteria for inclusion into the study were the following: (1) The operation was performed as a stand-alone surgery or in combination with hamstring release or plantar flexor release of the foot in association with single-event multi-level surgery. (2) No previous operation had been performed on the hip joints. (3) If bony surgery was performed along with soft-tissue release of the hip, this hip joint was excluded. Gross motor function classification system (GMFCS I-0, GMFCS II-8, GMFCS III-18, GMFCS IV-18, GMFCS V-27 patient). Migration percentage (MP) was measured on anteroposterior radiographs of hip joints in neutral rotation before and exactly 5 years after surgery. Data were analysed statistically.
***Results*** Average preoperative MP was 37.0 %, and 5 years after surgery it was 27.2 %.The difference was statistically significant ($p < 0.001$). The primary operation failed in 26 hip joints (18.4 %) because MP was = 50 % (9 hips) or an additional bony operation was performed (17 hips) within 5 years. There were no statistical differences between boys and girls ($p = 0.116$), when patients had simple release in the hip joint area, or when lengthening of the knee flexors was performed concurrently ($p = 0.240$). There was no statistically significant difference between age categories. Preoperative MP value impacted significantly on the result ($p < 0.001$). If preoperative MP was = 35 %, the primary operation failed in only 1 hip joint (1.4 %) in comparison with the group of patients with MP > 35 %, where soft-tissue release failed in 25 hip joints (36.8 %) ($p < 0.001$). When preoperative MP was ~50 %, operation failure ranges around 55 %.
***Conclusions*** Tenotomy of spastic adductors and flexors can fully correct hip subluxation or stop progression of lateral migration of the femoral head if it is performed before reaching the critical MP limit around 35 % and should be performed bilaterally at the same time.

***Significance*** The results of the study could help us improve our management of cerebral palsy patients with improvement in decision making about soft tissue surgery and its timing.

### EP12

### Surgical Treatment of Congenital Idiopathic Clubfoot After "French" Functional Treatment: Outcome in a 10-Year Prospective Study

**Nicolas Bocahut**[1], Philippe Souchet[2], Brice Ilharreborde[1], Keyvan Mazda[2]

Department of Orthopaedic Surgery, Paris, France; 2.Robert Debre Hospital, Paris, France

**LEVEL 2/Clubfoot**

Keywords: Clubfoot, à La Carte Surgery, Functional Method.

***Purpose*** There have been few studies with sufficient follow-up and power to report results and incidence of surgery in management of clubfoot, or congenital talipes equinovarus after "French" functional treatment or Ponseti method. The purpose of this study was to report the long-term results and incidence of surgical treatment in a prospective series of idiopathic clubfeet followed up from birth, managed functionally and if necessary with a secondary surgical procedure.
***Methods*** All the children with idiopathic clubfeet born between the 1st of January 1995 and the 31st of December 2006 and seen for the first time before the age of 3 month were included. They received functional treatment, comprising physical therapy and orthoses, and were followed up prospectively regardless of compliance, up to the 1st of March 2013. Initial assessment of severity was based on the Dimeglio score, and functional assessment at follow-up on the Bensahel score. When necessary, the surgical procedure performed was always a one-stage medioposterior release "à la carte" with or without calcaneal osteotomy.
***Results*** At a mean 10.3 years' follow-up (range 5.8–18.0 years), 155 clubfeet (48.3 %) had required surgical management (excluding Achilles tenotomies) among 321 feet treated functionally with complete follow-up. Clubfeet requiring surgery were initially more severe: Dimeglio 11.4/20 vs 8.6/20. Mean age at surgery was 22.1 months. Initial Dimeglio score correlated with incidence of surgery: 15.4 % (2/13) in group I, 30.7 % (47/153) in group II, 70.5 % (91/129) in group III and 100 % (13/13) in group IV. At latest follow-up, distribution according to the Bensahel evaluation was as follows: 65.8 % (102/155) in class I, 29.0 % (45/155) in class II, 4.5 % (7/155), 0.6 % (1/155) in class IV and none in the upper classes (V, VI and VII). Mean post-operative follow-up was 9.1 years (range 1.1–17.3 years), 94.9 % of good results 147/155 of surgically treated feet were in Bensahel class I or II at latest follow-up, meaning these 94.9 % of patients had good results. Only 8 bad results (class III or IV) were observed in this operated group. Eleven feet (7.1 %) needed more than one surgical procedure (average 1.4). Mean Bensahel score was poorer in the surgical than in the pure functional group: 4.0/50 vs. 1.4/50.
***Conclusions*** Many authors nowadays prefer the Ponseti method, because of a low rate of associated surgery. The present results confirm the high rate of surgery associated with the "French" functional method especially in feet initially classified Dimeglio III or IV but also the high rate of good results after surgery in all groups. This score at birth remains an essential predictive factor. Management of the most severe deformity remains a difficult challenge.
***Significance*** Although surgery was frequent, long-term functional results after surgery were very satisfactory in all feet. Therefore, the objective of reducing surgical incidence should not be at the cost of persisting deformity.

### EP13

### Ilizarov External Fixation for Management of Severe Relapsed Clubfeet in Older Children

Ashraf Atef

Tanta University, Tanta, Egypt

**LEVEL 3/Clubfoot**

Keywords: Clubfoot, TEV, Relapsed, Recurrent, Stiff, Ilizarov, Deformity.

***Purpose*** Although the standard treatment of clubfoot deformity is conservative by serial casting techniques, relapses are not uncommon. Management of relapsed clubfoot deformity in older children is an orthopedic challenge. There is a growing interest in management of such complex deformities using the Ilizarov technique.
***Methods*** In this study, the Ilizarov frame was used to correct severe relapsed clubfoot deformities in older children, who had all undergone previous surgical interventions. 42 relapsed clubfeet were included. The Dimeglio classification was used for clinical assessment of the relapsed feet pre-operatively as well as post-operatively.
***Results*** After an average follow-up period of 4.6 years, and according to the Beatson and Pearson numerical assessment, favorable results (excellent or good) were found in 37 feet, whilst poor results were identified in only five feet.
***Conclusions*** Based on the final clinical and radiographic results, the Ilizarov technique could be considered as a good management technique for such severe deformities.
***Significance*** This article adds to the general knowledge and to the pool of management of severe and relapsed cases after surgical correction in older children with residual deformities. The Ilizarov frame proved sufficient for management of such severe deformity cases.
This was published in The Journal of Foot and Ankle Surgery, J Foot Ankle Surg, 19 (2013) 177–181.

### EP14

### Relapsed Clubfoot After Ponseti Treatment: Idiopathic or Not?

**Vladimir Kenis**, Alexey Baindurashvili, Julia Stepanova

The Turner Institute for Children's Orthopaedics, Saint-Petersburg, Russia

**LEVEL 2/Clubfoot**

Keywords: Clubfoot, Ponseti Treatment, Relapse

***Purpose*** The Ponseti method of clubfoot treatment provides the best results in children with idiopathic clubfoot. Despite successful initial treatment, relapse is still a problem. The risk factors for relapse include non-compliance with bracing protocols, but even in children who wear braces properly relapse can occur. We postulated that in children with relapsed clubfoot. A non-idiopathic cause of the deformity should be suspected and thus the aim of this study was assess the incidence of concomitant neurogenic conditions in children with congenital clubfoot relative to the tendency to relapse.

***Methods*** A retrospective analysis of a consecutive series of 250 children with the diagnosis of idiopathic congenital clubfoot was done. All children were treated with Ponseti method with follow-up from 18 to 52 months. In 28 (11 %) patients different degrees of relapse were detected. For relapsed and non-relapsed group musculoskeletal, neurological and developmental disorders were assessed.
***Results*** Among developmental orthopaedic disorders DDH, metatarsus adductus and flatfoot of contralateral side in unilateral clubfoot were seen more often in relapsed group (not statistically significant). Delayed motor milestones achievement was statistically significant for sitting, and crawling for relapsed group ($p < 0.05$). Seizures were detected in 4 patients of relapsed group and were not seen in non-relapsed. Behavioral disorders were noted in 21 % of relapsed group and 3 % of non-relapsed. Delayed or altered speech and language skills were seen in 35 % children of relapsed group and 6.3 % of non-relapsed ($p < 0.05$).
***Conclusions*** Relapse of deformity after successful initial treatment of clubfoot with Ponseti method is associated with a higher incidence of neuro-developmental disorders. Seizures and speech problems had significantly higher incidence in relapsed patients.
***Significance*** Pathological signs of complex conditions can appear later during infancy, may be important predictors of prognosis and can lead to re-estimation of the idiopathic nature of the clubfoot deformity.

### EP15

### The Effectiveness of Prenatal Detection of Structural Congenital Talipes Equinovarus Deformity in An Unselected Population: Better Information For Better Counseling

**Michael Pullinger**[1], Victoria Easton[2], Thomas Southorn[2], Rachael Hutchinson[2], Richard Smith[2], Anish P Sanghrajka[2]

University of East Anglia, Norwich, UK; 2. Norfolk & Norwich University Hospital, Norwich, UK

**LEVEL 2/Clubfoot**
Keywords: CTEV, Prenatal Diagnosis.

***Purpose*** There is much inconsistency in the reported results of prenatal detection of congenital talipes equinovarus (CTEV) foot deformity. Positional deformities have been included in some studies, but not others. Most studies have been performed in specialist foetal medicine units and include a large number of tertiary referrals, making their results less relevant for screening in unselected populations. Some studies have become outdated as they used the need for open surgery as an outcome measure. This makes it very difficult to provide accurate information when counseling expectant parents.
Our aim was to determine the effectiveness of prenatal ultrasound scanning in the detection of structural CTEV (i.e. deformities which required the Ponseti treatment) in an unselected population. This was to allow us to provide parents with accurate, factual information regarding the reliability of scan results during the counseling process.
***Methods*** This study was performed at a University teaching hospital with specialist tertiary level foetal medicine and children's orthopaedic units, with a catchment population of over 1 million.
The database of the antenatal ultrasound department was interrogated for all suspected cases of CTEV on the 18 + 0–20 + 6 week foetal anomaly ultrasound scan, between August 2006 and June 2012. Terminations, stillbirths and tertiary referrals were excluded from the study.
The Ponseti-service database was searched to identify all patients treated for structural CTEV between January 2007 and November 2012. Cases were excluded if the mother had not received the antenatal care during that pregnancy at our centre.
Results from the two searches were cross-referenced, and statistical analysis performed. Case-notes of the mothers of all the children treated for CTEV were also reviewed.
***Results*** During the study period, there were 30,077 prenatal scans and 34,373 live births at the hospital. CTEV was detected on prenatal ultrasound scans in 74 cases. After exclusions, 37 cases remained eligible for analysis. Of the 37 cases in which prenatal scans were positive for CTEV, 30 newborns were found to have structural CTEV at birth. The positive predictive value of a scan suggestive of CTEV was therefore calculated to be 81.1 %.
Over the study period, 48 patients were treated for structural CTEV; after exclusions, 42 patients (54 feet, CTEV-incidence 0.001) were eligible for the study. Of these 42 children, 30 had been detected prenatally; sensitivity of ultrasound scanning for CTEV was therefore calculated to be 71.4 %.
The negative predictive value and specificity were over 99.5 %.
***Conclusions*** Prenatal ultrasound scans have a good sensitivity for the detection of CTEV, and an excellent positive predictive value when applied to an unselected population. However, these scans are not completely reliable for the detection or exclusion of structural CTEV deformity.
***Significance*** This is important information for Orthopaedic surgeons that are involved in the prenatal counseling of expectant parents. Parents should be made aware that a positive finding on an ultrasound scan means that there is an 80 % chance that their child will require treatment for CTEV. However, parents and clinicians should be aware that just over a quarter of cases of CTEV will be missed on prenatal scans.

### EP16

### Comparison of Osseous and Cartilaginous Acetabular Angles on MRI in Children

**Sophie Merckaert**, Pierre-Yves Zambelli

CHUV-Pediatric Orthopedic Surgery, Lausanne, Switzerland

**LEVEL 3/DDH**
Keywords: Hip Dysplasia, Residual Dysplasia, MRI, Acetabular Reorientation Surgery.

***Purpose*** In patients with residual developmental hip dysplasia, plain radiography of the pelvis can show an insufficiency of osseous acetabular coverage of the proximal femoral epiphysis but we are not able to quantify the quality of the real coverage of the femoral head consisting of the acetabular bone, cartilage and labrum. The MRI allows good tissue differentiation between the bone, the cartilage and fibrous tissues. The measure of the cartilaginous angle of Hilgenreiner (C-HTE) appreciates the morphology of the cartilaginous borders of the acetabulum, which will gradually ossify with growth. This cartilaginous HTE defines the theoretical cover in a mature hip.
The purposes of this study was to find a method to both measure and classify hips presenting with residual dysplasia and to find a consensus in indication for acetabular reorientation surgery.
We performed coxometric analysis of the cartilaginous and osseous limits of the acetabulum on frontal MR imaging from healthy and dysplastic hips in order to find a consensus in indication for secondary surgery in children with residual hip dysplasia.

***Methods*** 30 children (9 boys and 21 girls, mean age 58 months) who had MR imaging of both hips were included in this study. The diagnoses for the 60 hips were 24 healthy hips, 11 hips treated for Perthes disease (control group) and 23 hips with DDH (pathologic group). 2 hips were excluded because of irreducible late diagnosed dislocation. We measured the cartilaginous and osseous angles of Hilgenreiner (HTE) on both hips on T2 weighted frontal images and calculated the ratio of the square of cartilaginous HTE (C-HTE$^2$) above the osseous acetabular index (O-HTE). This ratio C-HTE$^2$/O-HTE expresses the residual cartilaginous growing-potential of the acetabulum.

***Results*** For the healthy hips, the ratio varies from 0.07 to 3.85. The hips with DDH show a ratio from 4.52 to 23.3 with 87 % above 5, including 17 % with a ratio above 10. The hips with Perthes disease show a mean ratio of 2.3 with no hip above 5.

The comparison of the ratios between the pathologic group and the healthy group, respectively the group with Perthes was highly significant, but we didn't find a difference between the healthy hips and those with Perthes disease.

***Conclusions*** Considering a mature hip, the value of the osseous angle tends towards that of the cartilaginous angle; the ratio C-HTE$^2$/O-HTE is then equivalent or inferior to the O-HTE. The mean value of the O-HTE at end of growth was evaluated by Bédouelle et al. at 12° at the age of 8 to 10 years and for younger children between 15° and 20°.

The ratio C-HTE$^2$/O-HTE enables us to classify the hips in 3 categories: Group A includes hips with a ratio inferior to 5. Group B includes hips with a ratio from 5 to 10 and group C includes hips with a ratio above 10. Group A represents the hips that are considered as normal, with a great potential of standardization with growth.

Group B includes hips that have less optimal correction potential, but still have a chance of normalization with growth and Group C represents severely dysplastic hips in which the cartilaginous potential of growth is insufficient and where a surgery of reorientation of the acetabulum is indicated.

***Significance*** We propose MRI imaging at the age of 4 year if standard x-ray still shows signs of dysplasia after conservative treatment.

### EP17

### Does Abduction Brace Treatment of DDH Influence Motor Development?

**Marcin Zgoda**[1], Piotr Wasilewski[2], Dominik Golicki[3]

Department of Orthopaedics, Crosshouse University Hospital, Kilmarnock, UK; 2. Department of Orthopaedics and Traumatology of the Locomotor System Warsaw Medical University, Warsaw, Poland; 3. Department of Pharmacoeconomics, The Medical University of Warsaw, Warsaw, Poland

**LEVEL 3/DDH**

Keywords: Developmental Dysplasia of the Hip (DDH), Treatment, Walking Age.

***Purpose*** To assess the influence of treating developmental dysplasia of the hip (DDH) with the abduction brace on locomotor development in children.

***Methods*** One hundred children treated for DDH served as the study group. There were 80 girls and 20 boys. The children's average age at the beginning of the treatment was 8 weeks. The control group consisted of 100 healthy children with normal hips and without any locomotor system disorders. We have evaluated factors such as the age at which the treatment started, the duration of the treatment, the birth weight of the child and the time when the children started sitting and walking independently.

***Results*** On average, treatment with the abduction brace lasted 13 weeks (ranging from 6 to 26 weeks). The mean age at which the patients began to sit was 7 months, which was one week later compared to children from the control group ($P = 0.28$). The age at which they started walking was 12 months and 2 weeks, which was 3 weeks later than in the control group ($P = 0.002$).

***Conclusions*** For children with DDH, the abduction brace is a safe and effective method of treatment and, although the infants begin to walk about 3 weeks later compared to healthy children, this practice does not seriously affect the child's locomotor development.

***Significance*** To our knowledge this is a first study that evaluates the influence of bracing on locomotor development in children with DDH.

### EP18

### Early Treatment of Developmental Dysplasia of Hip With Tubingen Hip Flexion Splint: Our Experience

**Vito Pavone**, Gianluca Testa, Maria Riccioli, Luca Cannavò, Giuseppe Sessa

A.O.U.P. Vittorio Emanuele-University of Catania, Catania, Italy

**LEVEL 4/DDH**

Keywords: Hip, Developmental dysplasia, Conservative management, Timing, Tubingen splint.

***Purpose*** Developmental dysplasia of the hip (DDH) is an anomaly of the hip joint. In patients with early diagnosis, within 3/6 months of life, the treatment is fundamentally conservative and involves the use of a dynamic harness. The indication for the use of the Tübingen hip flexion splint is a dysplastic hip. The aim of this study is to report the experience of the Orthopaedic Clinic of the "University of Catania" regarding conservative treatment of dysplastic hips diagnosed in children within 6 months of life.

***Methods*** From January 1997 to July 2012, 5137 infants (10,274 hip) underwent an ultrasonographic assessment of their hips within first 3 months of life and were treated for hip dysplasia with Tubingen hip flexion splint. The start and the duration of treatment, rate of success and complications were evaluated.

***Results*** A total of 351 (6.83 %) patients affected by DDH for a total of 544 dysplastic hips (5.3 %) were treated with the Tübingen hip flexion splint. Therapy was started on average at 39 days of life. Harnesses were worn for 24 h a day and applied for a mean of 3.8 months. Thus 482 (90.44 %) dysplastic unstable or dislocated hips were successfully converted into type I hips with an alpha-angle of more than 64° in the splint. Complications were reported in 3 (0.55 %) patients.

***Conclusions*** Onset, typology and duration represent important aspects of conservative treatment of DDH. It is essential to start treatment as early as possible using a dynamic harness, such as the Tubingen harness, inspired by the model proposed by Pavlik, to prevent adduction and extension of the hips. The duration of treatment depends on the age of the child and on the severity of the DDH. Failures of treatment are mainly due to inappropriate use of the brace. The most common complication was represented by avascular necrosis of the femoral head.

Dysplastic unstable and dislocated hips can be treated successfully with the Tübingen hip flexion splint, reporting good clinical and ultrasonographic outcomes.

***Significance*** No statistically significant relationship was found between the duration of therapy and the time when treatment was started, early within the first week of life or late (p = 0.152).

### EP19

### Evaluation of the Ultrasound Alpha Angle in At-Risk Infants

**Ajay Asokan**, Evangelia Protopapa, Paul Humphries, Andreas Roposch

Great Ormond Street Hospital for Children; UCL Institute of Child Health, London, UK

**LEVEL 1/DDH**
Keywords: DDH, Ultrasound, Alpha angle.

***Purpose*** An alpha angle of 60° at the age of 6 weeks is regarded as normal. However, it is unclear if infants with lower alpha values at 6 weeks can be discharged or need repeat ultrasound. We aimed to determine the number and outcomes of infants presenting with an alpha angle <60° at the age of 6 or more weeks.
***Methods*** Between 2010 and 2013, infants with risk factors were included in this prospective study. They underwent hip ultrasound at 6 weeks. Ultrasounds were performed by trained professionals in dedicated clinics. Image acquisition was according to Graf. Images were reviewed by a team of experts in consensus for the purpose of this study. We considered abnormal hips to be those showing an alpha <60° at age 6 or more weeks.
***Results*** 1,945 patients were included. At 6 weeks or greater, 102 infants (5.2 %) exhibited alpha <60°, with a mean angle of 56° (range 45°–59°). All such hips were re-scanned and improved without treatment to 60° or greater.
***Conclusions*** This prospective study demonstrated that in infants 6 weeks or older, a mean alpha angle of 56° ensured spontaneous progression to a value of >60° in all cases.
***Significance*** The data suggests that infants exhibiting an alpha of 56° at age 6 or more weeks do not warrant further ultrasound follow-up because their hips will improve to values >60°.

### EP20

### Natural History of Hip Instability in Infants (Without Subluxation or Dislocation)

**Blazej A Pruszczynski**[1], H Theodore Harcke[2], Laurence Holmes[3], J Richard Jr.[4]

1. Department of Orthopedics and Pediatric Orthopedics, Lodz, Poland; 2. Medical University of Lodz, Wilmington, USA; 3. Department of Radiology, Wilmington, USA; 4. Nemours/Alfred I. duPont Hospital for Children, Wilmington, USA

**LEVEL 2/DDH**
Keywords: Development dysplasia of the hip, Hip instability in infants, Ossification of the acetabulum, Sonography.

***Purpose*** The natural history of hip instability (without subluxation or dislocation) and treatment in infants remain controversial. We performed a retrospective cohort case-only study with blinded, prospectively collected data to assess normalization of the acetabular index (AI) in consecutive untreated infant hips with sonography instability.
***Methods*** Consecutive hips meeting inclusion criteria were followed by sonography/radiography and data analyzed using tabular and regression models.
***Results*** In 48 hips, AI measured by radiography normalized within 3 years of age without treatment. Normalization occurred: by age 7 mos in 35 %, 12 mos in 67 %, 18 mos in 75 %, 24 mos in 81 %, and 36 mos in 100 %. Two patterns of normalization of the AI were observed: group I showed ossification in a physiological range of normal by 7 mos of age, and group II had delayed ossification with later normalization of the acetabular index measurement. Breech presentation (p = 0.013) and cesarean delivery (p = 0.004) correlated statistically, directly, with a later normalization.
***Conclusions*** The natural history of infant hip instability (without subluxation or dislocation), which is reduced at rest and unstable with stress as diagnosed by the Harcke method of sonography, has spontaneous normalization of the AI within 3 years of age. We suggest three patterns of acetabular ossification in unstable infants' hips: (I) normal ossification, (II) delayed ossification with normalization of the AI by age 3 years, and (III) defective secondary centers of ossification with an upward tilt of the lateral acetabular rim in adolescence.
***Significance*** Unstable infant hip which is reduced at rest and unstable with stress has history of spontaneous normalization of the AI within 3 years of age with three possible acetabular ossification patterns: (I) normal ossification, (II) delayed ossification with normalization of the AI by age 3 years, and (III) defective secondary centers of ossification with an upward tilt of the lateral acetabular rim in adolescence.

### EP21

### Successful Pavlik Harness Treatment for Developmental Dysplasia of the Hip and Normal X-ray at Age Two: Is a Longer Follow-up Necessary?

**Nanni Jeanine Allington**, Isabelle Schrouff

CHR Citadelle, Liege, Belgium

**LEVEL 3/DDH**
Keywords: Developmental dysplasia of the hip, Long term follow-up.

***Purpose*** A selected group of patients successfully treated for developmental dysplasia of the hip (DDH) with a Pavlik harness (PH) might not require long term follow-up with irradiating X-rays.
***Methods*** A retrospective review of a consecutive series of patients treated for DDH between January 1995 and July 2004 was undertaken. A very selected group was then defined: the child had to be otherwise normal (neuromuscular diagnosis or syndromes were excluded), the treatment with the PH had to be successful, and the X-ray at age 2 had to be normal (normal acetabular index, no signs of avascular necrosis).
At last follow-up a clinical exam was performed and the following measurements were performed on the radiographs: center-edge angle, Seringe-Severin score, teardrop, acetabular index (open growth plate), Sharp's angle (mature), Mose's classification.
***Results*** Out of 170 consecutive children seen with DDH, 130 fitted those criteria: 47 had insufficient data at follow-up and thus 83 were studied. 22 had bilateral DDH and a total of 105 hips were thus available for review. Of these 105 hips, 12 were Ortolani positive, 10 were Barlow positive, 83 were diagnosed at one month of age by dynamic ultrasound (done for risk factors) with subluxation/instability/dysplasia. The mean follow-up was of 10 years and 4 months (range 8 to 16 years).

All 105 hips had a normal clinical exam as well as normal X-ray at the last follow-up: mean center-edge angle of 32° (25°–40°), Seringe-Severin score IA, normal teardrop, mean acetabular index 18° (9°–24°), mean Sharp's angle of 35° (32°–47°), Mose's circle below 2.
***Conclusions*** Since the clinical exam and the X-ray done at long term follow-up were normal it might not be necessary to continue follow-up if X-ray at age two are normal after treatment with the PH in otherwise normal children.
***Significance*** Although the results of this study suggest that long term follow-up might not be required in this selected group. An increase of the number of patients as well as a still longer follow-up (not all were skeletally mature) would be useful.

### EP22

### One Stage Treatment of Late Diagnosed Bilateral Hip Dysplasia with Combined Surgical Technique Including Salter Innominate Osteotomy

**Haluk Agus**, Muhammed Bozoglan, Kalenderer Onder, Burak Onvural

Tepecik Training Hospital, Izmir, Turkey

**LEVEL 3/DDH**
Keywords: Salter osteotomy, Combined procedure, One stage, Bilateral hip dysplasia.

***Purpose*** The purpose of this study is to analyze the results of combined surgical treatment methods including Salter innominate, femoral shortening and derotational osteotomy for bilateral developmental dysplasia of hip (DDH) diagnosed at walking age which is done at one stage for both hips.
***Methods*** 24 hips of 12 children with DDH which was diagnosed at walking age (and with no previous treatment) were evaluated (Group I). There were 10 females, 2 males and their mean age was 21.7 months (18–27 months). Hips were classified according to the Tönnis classification system. The amount of acetabular correction in dysplasia was calculated radiologically by the measurement of acetabular index before and after the operation. All measurements of both hips were compared with each other and with the measurements of an identical 12 dysplastic hips of 12 children (Group II) who had unilateral DDH treated with the same method and evaluated statistically using the Mann–Whitney $U$ test. Acetabular index and CE angle values of both hips in group I at last control were also compared with each other. Osteonecrosis of femoral head according to Kalamchi and MacEwen classification and clinical outcome according to McKay criteria were evaluated at last control for Group I.
***Results*** Mean follow up period for group I was 4.4 years (2–6 years). Differences of in the amount of acetabular correction of both hips compared with each other in group l and separately with the correction rates of hips in group II were not statistically meaningful. The osteonecrosis rate was 16.6 %. There were 7 (58.3 %) excellent, 3 (25 %) good and 2 moderate (16.6 %) clinical results.
***Conclusions*** One stage treatment of bilateral dysplastic hips with combined surgical treatment methods including Salter innominate, femoral shortening and derotational osteotomy is an effective treatment method. Although it is more invasive, clinical results and complication rates are comparable with the results of bilateral DDH cases treated with the same method in multiple stages.
***Significance*** This abstract suggests that Salter innominate osteotomy can be done safely for both hips in bilateral DDH simultaneously in contrast to its prerequirements and these cases can also be surgically treated by combined methods safely in one stage.

### EP23

### Early Treatment of Congenital Dislocation of the Hip with Pavlik Harness

**Alexandre Francisco Lourenco**[1], Paulo Kenji Honmoto[1], Simone Battibugli[2], Giovanna Galvão Braga Motta[1]

Federal University of São Paulo, São Paulo, Brazil; 2. MEDCARE Orthopedic and Sipne Hospital, Dubai, United Arab Emirates

**LEVEL 4/DDH**
Keywords: DDH, Ortolani sign positive, Hip dislocation, Pavlik harness.

***Purpose*** Report 10-year experience with early use (application at the first or second day of life) of Pavlik harness in infants with frankly positive Ortolani manoeuvre.
***Methods*** 78 newborns (135 hips; Bilateral 57, Left Hip 12 and 9 Right Hip). Type of birth presentation: 39 breech, 39 cephalic. 64 % female, 36 % male. 14 % of the babies presented with a positive family history of DDH. The Pavlik harness was applied during the first 2 days of life and used in a full time regimen for 2 weeks (24 h a day). The follow-up was done by clinical examination (same orthopedist) each 7 days, US control (same doctor) each 14 days until reaching the subtype Ia of Graf's ultrasound classification and X-ray up to 30 months of age.
***Results*** On reassessment after two weeks, 117 (87 %) of the hips had a negative Ortolani's manoeuvre and 18 hips (13 %) were still dislocated. In the third week only 4 (3 %) of the hips had still not reduced and at this point the treatment with Pavlik harness was suspended and a closed reduction under anesthesia and spica cast application was indicated. The average alpha angle on the first ultrasound (performed at 2nd treatment week) was 45.40 (range 0–50) and the final was 65.26 (61–74). Mean use of Pavilik was 95.63 days (45–145 days).
***Conclusions*** The very early application of Pavlik harness (within the first two days of life) showed excellent results. Out of 135 dislocated hips, 129 (97 %) obtained reduction in 3 weeks. The average treatment time with complete resolution of dysplasia was 95 days. The harness full-time use in the first weeks after early diagnosis enables development of normal hips in a condensed treatment time frame. There were no serious complications with this method. The only changes found were skin rashes, which resolved without sequelae. The use of ultrasound is critical for the follow up and also to determine the treatment cessation.
***Significance*** The very early initial treatment with the Pavlik Harness might have allowed the excellent results encountered in this cohort of patients with 97 % of reduction in less than 3 weeks, only 3 % of the hips were treated with closed reduction. The literature shows up to 40 % of failure on the treatment with Pavlik harness for dislocated hips (Ortolani sign positive).

### EP24

### Late Diagnosed Congenital Dislocation of the Hip (After One Year of Age) in France: Prospective Study and Analysis Of Screening Pitfalls

**Philippe Wicart**, Christian Morin

French Society of Pediatric orthopedics, Paris, France

**LEVEL 4/DDH**
Keywords: Dislocation hip late diagnosis.

***Purpose*** A preliminary study displayed a significant ($p = 0.0000001$) increasing of late diagnosed (after one year old) congenital dislocation of the hip (CDH) from 2003 to 2010 in France, suggesting a precise update in 2010.
***Methods*** The French Society of Pediatric Orthopedics conducted an exhaustive national prospective study analysing the incidence of late diagnosed CDH in 2010 in France and their social/medical characteristics.
***Results*** The study evidenced 70 children with late diagnosed CDH. They were males in 17 % of cases. The CDH was bilateral for 3 children (4.3 %). The CDH was right in 51.5 % of cases. Average age at diagnosis was 20 ± 10.2 months old. A vast majority of children were born in 2008 and 2009. The incidences of late diagnosed CDH during 2010 in France for children born in 2009 and 2008 were respectively 2.9/100.000 and 4/100.000 births. Social origin and type of birth or medical institution did not influence this risk. The parents worried by a limp were at the origin of the diagnosis in 85.9 % of case. One CDH risk factor was noticed in 27.4 % of children and one child had 2 risk factors. Then, there was at least one risk factor in 28.8 % of children. Clinical screening was performed at birth for 90 % of children but a disorder was observed only in 7 % of cases without inducing an alert. The rate of unchecked children during the first 3 months of life was 28%, and 64 % between 3 and 12 months old. Only 10.9 % of children underwent a sonography and 2.8 % a pelvis X-ray before 1 year old.
***Conclusions*** This series reveals the recent failure of CDH screening in France, due to a lack of clinical examination and sonography and/or Xray under-prescription. These data motived a new campaign for CDH screening and new national prospective study involving also radiologists and pediatricians.
***Significance***: Prospective study.

### EP25
### Long-Term Results of Triple Pelvic Osteotomy in Developmental Dysplasia of the Hip

**Klein Céline**, Glorion Christophe, Fontanarosa Antonio, Seringe Raphaël, Wicart Philippe

Hopital Necker Enfants Malades, Paris, France

**LEVEL 4/DDH**
Keywords: Triple pelvic osteotomy, Acetabular dysplasia, DDH.

***Purpose*** Acetabular dysplasia is an essential component of bone deformities observed in developmental dysplasia of the hip (DDH). Triple pelvic osteotomy (TPO) may be performed in order to correct this architectural flaw. The goal of this study was to evaluate clinical and radiological long-term results and complications of this procedure.
***Methods*** Thirty-seven children (50 hips), who underwent surgery between 1975 and 2005, were included. Average age at surgery was 6.3 years old (3–15.2 yrs). TPO was mainly indicated for residual acetabular dysplasia after closed reduction (n = 48). 34 hips underwent previous surgery: 29 femoral osteotomies, 3 open reductions and 2 Salter osteotomies. Clinical evaluation was assessed with Harris hip score and modified Postel Merle d'Aubigné (PMA) score. Radiological evaluation included Wiberg angle (VCE) and acetabular index (AI) (preoperative, postoperative, last follow-up). At the final evaluation, the results were classified according to the Severin score and vertical center anterior angle (VCA) was measured.
***Results*** Average follow-up was 14 years (4.6–28.7 yr) with an average age of 20.1 years old. Average Harris hip score was 94 (45–100) and 17/18 (7–18) in the PMA score with respectively 94 % (n = 47) and 90 % (n = 45) with good or excellent results. The VCE and the AI were significantly improved, respectively, from 10° ± 5° in preoperative to 33° ± 8° in postoperative and 30° ± 10° to 5° ± 9.5°. The modified Severin score was I (42 % of cases), II (40 %), III (10 %), IV (6 %) and VII (2 %). Average VCA was 40° ± 9°. At last follow-up and with closed triradiate cartilage (n = 41), VCE, AI and VCA (n = 33) exceeded the upper limit value respectively, in 12 (27 %), 6 (15 %) and 24 hips (72 %). Fifteen hips (36 %) showed acetabular retroversion (cross-over sign). Complications included 2 secondary displacement of the osteotomy, 24 (48 %) nonunion of iliopubic and/or ischiopubic branches (performed extraperiosteally) and 2 femoral head necrosis.
***Conclusions*** TPO efficiently corrects an isolated acetabular dysplasia or dysplasia associated with mild subluxation. However, the high risk of anterior and lateral coverage overcorrection justifies understanding the importance of acetabular switch. Unsatisfactory results (Severin III, IV, VII) were observed for patients with significant subluxation requiring other surgical steps. Iliopubic and ischiopubic nonunions, and induced pelvic growth disturbances, should be prevented by subperiosteal osteotomies.
***Significance*** Retrospective study.

### EP26
### Reverse Ponseti-Type Method for Congenital Vertical Talus: Comparison Between Idiopathic and Teratologic Patients

Jonny Wright, Di Coggings, Claudia Maizen, **Manoj Ramachandran**

The Royal London and Barts and The London Children's Hospitals, London, UK

**LEVEL 2/Foot and ankle**
Keywords: Congenital vertical talus, Reverse Ponseti.

***Purpose*** Congenital vertical talus (CVT) has been historically treated with extensive soft tissue releases with significant associated complications. Recently, reverse Ponseti-type casting followed by percutaneous reduction and fixation has been described with excellent results in separate cohorts of either idiopathic or teratologic cases of CVT. There are currently no studies that compare the outcomes between the two types.
***Methods*** We present a prospective cohort of 13 patients (21 feet) in which this technique has been used in both idiopathic and teratologic associated cases of CVT. Clinical, radiographic (Adelaar score) and parent-reported outcomes (PODCI score) were obtained at a mean follow up of 36 months (range 8–57).
***Results*** Six patients (9 feet) had associated neuromuscular conditions or syndromes; seven patients (12 feet) were idiopathic. Initial correction was achieved in all patients with significant improvement in all radiographic parameters. Recurrence was seen in 10 out of 21 feet. Of the recurrences, 6/9 feet were in the teratological and 4/12 feet in the idiopathic patient group. The difference in the recurrence rate between the two groups did not extend to statistical significance (p = 0.198).
There was no significant difference between the Adelaar score of the teratological group and the idiopathic group (median 9 vs 9;

$p = 0.72$). The PODCI global function score was 87 in the idiopathic group and 45 in the teratological group, likely reflecting the reduced global function due to the underlying disorders present in the latter group. The pain/comfort scores were similar with an average of 95 (85–100) in the teratological group and all patients scoring 100 in the idiopathic group.
***Conclusions*** The reverse Ponseti-type technique is effective in initial correction of both idiopathic and teratologic cases of congenital vertical talus. Recurrence is a problem in both these groups, with higher rates than first reported in the original paper. However, these rates are less than those reported for open surgical releases. Modification of the technique to include limited capsulotomy at the initial operation may reduce the risk of recurrence.
***Significance*** Although the reverse Ponseti is a successful technique in both idiopathic and teratologic vertical talus and limits the extensive surgery that was historically used to correct this condition with resultant significant complications, there is a higher risk of recurrence than initially reported. This recurrence rate could be modified by modification of the extent of the initial limited surgical technique.

### EP27

### Hip Instability in Patients With Down's Syndrome: Evaluation of Surgical Management

**Mira Ramanoudjame**, Vicken Topouchian, Christophe Glorion, Georges Finidori

Hospital Necker Enfants Malades-APHP, Paris, France

**LEVEL 3/Hip/Lower extremity**
Keywords: Hip instability down syndrome.

***Purpose*** The hyperlaxity associated with Down's syndrome can induce hip instability with total or partial dislocation, the treatment of which is notoriously difficult. The present study assessed the results of surgery for hip instability with partial dislocation in Down's syndrome.
***Methods*** Fifteen unstable hips in 14 patients were operated on between 1982 and 2012. Eight hips with partial dislocation (mean age, $8.4 \pm 2.7$ years) were managed by femoral varization-derotation osteotomy associated with pelvic osteotomy in 7 cases (4 Degas, 2 Salters, 1 triple) and capsulorrhaphy (group A). Seven hips with more evolved dislocation (mean age, $14.1 \pm 2.6$ years) were managed by isolated pelvic osteotomy without capsulorrhaphy (group B). Preoperatively, mean acetabular index was $18° \pm 12°$ ($14° \pm 7°$ in group A, $23° \pm 16°$ in group B). Mean Reimers' index was $36 \pm 17$ % and mean cervico-diaphyseal angle $141° \pm 5°$ with no significant intergroup difference.
***Results*** Mean follow-up was $5.8 \pm 4$ years. Ten hips (62 %) were at end of growth. At end of follow-up, pelvic architectural disorder was corrected in group A, while there were residual defects in 3 patients (42 %) in group B. In group A, mean acetabular index was $6.6° \pm 7°$, Reimers' index $2 \pm 5$ %, and cervico-diaphyseal angle $113° \pm 12°$. In group B, mean acetabular index was $15.7° \pm 6°$, Reimers' index $11 \pm 13$ %, and cervico-diaphyseal angle $140° \pm 11°$. Three cases required revision surgery: in group A, 1 Chiari osteotomy for secondary dislocation; in group B, 1 femoral varization-derotation shortening osteotomy, and 1 total hip replacement. At end of follow-up, all hips were stable.
***Conclusions*** Surgery should be complete (femoral and pelvic osteotomy and capsulorrhaphy) and early in case of progressive hip dislocation: incomplete or late surgery invites failure. Adapted surgery can stabilize the hip in children with Down's syndrome.
***Significance*** Statistically significant.

### EP28

### Hip Dysplasia in Charcot-Marie-Tooth Type 1a: Evidence for Acquired Dysplasia

**Marie Gdalevitch**[1], Katie R Willis[2], Joshua Burns[2], Paul Gibbons[2]

Shriners Hospital for Children, Montreal, Canada; 2. Children's Hospital at Westmead, Sydney, Australia

**LEVEL 3/Hip/Lower extremity**
Keywords: Charcot-Marie-Tooth, Hip dysplasia, Hip reconstruction.

***Purpose*** Charcot-Marie-Tooth type 1A (CMT1A) is one of the most common inherited peripheral neuropathies. The incidence of radiographic hip dysplasia in CMT1A is reported at 16 % and directly correlated with a valgus neck shaft angle (NSA). The pathogenesis of dysplasia is unknown but may be due to weakness of proximal musculature. The purpose of this study is to retrospectively determine the incidence of radiographic hip dysplasia in children with CMT1A and assess the progression of their dysplasia.
***Methods*** Medical records of 66 children with a confirmed diagnosis of CMT1A between 2000 and 2011 were reviewed. Fifty-five children (110 hips) had at least one antero-posterior preoperative radiograph of the pelvis available. Radiographs were assessed for eight abnormalities: a center edge angle (CEA) less than 20°, a migration percentage greater than 20 %, a break in Shenton's line, a wide tear drop, a medio-lateral joint space ratio greater than 2, a NSA greater than 147°, an acetabular index greater than 24° and an upward angulation of the sourcil. Twenty-two children had more than one radiograph available and their initial and final radiographs were assessed for progression to dysplasia. Radiographic dysplasia was defined as a CEA of less than 20.
***Results*** On initial radiographs 9 children (16 %), 13 hips (11 %), had dysplasia and each dysplastic hip had four or more radiographic abnormalities. All children with dysplasia had an abnormal CEA with an average of 1.85 (range −35 to 15), an abnormal migration percentage with an average of 44.65 (range 24 to 83) and at least two other radiographic abnormalities. Two children (3 hips) that initially did not meet radiographic criteria for dysplasia developed dysplasia on subsequent radiographs taken within less than 2 years. Two of those hips only had 1 radiographic abnormality when first assessed. At final assessment, 11 children (20 %), 16 hips (15 %), had dysplasia. Five of the dysplastic hips (31 %) had a normal NSA. Average age at diagnosis of dysplasia was 9.7 years (range 7 to 15).
***Conclusions*** Incidence of hip dysplasia in CMT1A patients is 20 %—higher than the previously published rate. Pathogenesis remains unclear since one-third of children with dysplasia had a normal NSA. However, children that had four or more radiographic abnormalities all had hip dysplasia.
***Significance*** CMT1A patients with hip dysplasia should be treated aggressively since the dysplasia is severe and can progress quickly. A screening radiograph is recommended by age 4, then every 2 years.

### EP29
### Results of Tibial Epiphysiodesis Using Eight-Plate in Leg Length Discrepancy

**Elodie Gaumetou**, Brice Ilharreborde, Georges François Penneçot, Keyvan Mazda

Hopital Robert Debré, Paris, France

**LEVEL 4/Hip/Lower extremity**
Keywords: Leg length discrepancy, Epiphysiodesis, Eight-plate.

***Purpose*** Among the numerous epiphysiodesis procedures described in the literature, the guided growth principle of the 8-plate technique, initially described for angular deformities correction, has progressively gained popularity among paediatric orthopaedic surgeons to treat lower limb discrepancy (LLD). The goal of this study was to assess the efficacy of the 8-plate procedure for tibial epiphysiodesis in LLD.
***Methods*** All children treated for LLD using tibial 8-plate were prospectively included. A minimum 2-year follow-up was required. Efficacy of the tibial epiphysiodesis was calculated at 6 months postoperative and at latest follow-up, according to a validated method, previously published for percutaneous epiphysiodesis using transphyseal screws (PETS). Radiological measurements were performed on low-dose stereoradiography (EOS) using 3D segmental lengths measurements. Intraoperative and postoperative complications were reported.
***Results*** 26 patients (mean age 12.6 years at surgery) were included. The efficacy was only 17 % at 6 months and 31 % at follow-up. The epiphysiodesis effect appeared to be greater at follow-up when the screws were initially introduced in parallel position, compared to the angled position (40 and 30 %, respectively), within the plate. In 7 cases (26.9 %), the epiphysiodesis was completely inefficient during follow-up (0 % growth reduction). The angle between the 2 screws significantly increased during follow-up, but the progression of the angle was not correlated to the epiphysiodesis efficacy. No intraoperative complication occurred, and no valgus deformity was reported during follow-up. Six patients (23 %) presented pain on the plate and 4 revisions for material removal were performed. In addition, 4 (15 %) underwent revisions, 1 for infection, 1 for plate repositioning, and 2 for insufficient growth arrest.
***Conclusions*** Results of the current series show that the eight-plate technique is inappropriate for tibial epiphysiodesis in LLD. The procedure is simple and was associated with few perioperative complications, but the growth arrest observed at follow-up only reached 31 % of the expected gain, twice inferior to the one previously reported with PETS using the same calculation method. The influence of patient age, screw direction and operative timing need to be further studied.
***Significance*** The eight-plate technique is inappropriate for tibial epiphysiodesis in LLD.

### EP30
### Reliability of Radiological Criteria for Anticipating a Pathological Fracture in Simple Bone Cysts of the Proximal Femur

**Ozren Kubat**[1], Marko Šimunovic[2], Marko Bergovec[1], Kristina Potocki[2], Darko Anticevic[1]

1. Department of Orthopedic Surgery, Clinical Hospital Centre Zagreb, Zagreb, Croatia; 2. Department of Radiology, Clinical Hospital Centre Zagreb, Zagreb, Croatia

**LEVEL 3/Hip/Lower extremity**
Keywords: Bone cyst, Proximal femur, Pathologic fracture, radiography.

***Purpose*** Simple bone cyst (SBC) located in a biomechanically unfavourable area such as the proximal femur significantly raises risk of pathological fracture (PF). Various radiological criteria (bone cyst index-BCI, bone cyst diameter-BCD; minimal cortical thickness-MCT) have been suggested to help predict which cysts will lead to PF. The aim of this study is to evaluate these criteria.
***Methods*** Twenty-six patients treated at our department from 1981 to 2008 for proximal femoral SBC were divided into two groups, with presence of PF at presentation being the difference between groups. Pertinent radiological criteria were measured (BCI, BCD-longitudinal and transversal, MCT-medial and lateral) on plain radiographs.
***Results*** Average age of the patients was 10.6 years (range 3–20 years). Diagnosis of proximal femoral SBC was confirmed in all patients. Group A consisted of 12 (9 male, 3 female) patients who presented with PF through the proximal femoral SBC. Median BCI was 4.19 (IQR 3.08, Q1 2.96, Q3 6.04). Median BCD (mm) longitudinally was 48.15 (IQR 34.2, Q1 37.8, Q3 72), transversely 27 (IQR 15.75, Q1 23.4, Q3 39.15). Median MCT (mm) medially was 1.8 (IQR 1.4, Q1 0.9, Q3 2.3), laterally 1.8 (IQR 1.8, Q1 0.9, Q3 2.7). Group B included 14 (12 male, 2 female) patients without PF through proximal femoral SBC at presentation. Median BCI was 4.26 (IQR 2.85, Q1 2.83, Q3 5.68). Median BCD (mm) longitudinally was 54.9 (IQR 34.2, Q1 42.3, Q3 76.5), transversely 29.2 (IQR 14.4, Q1 21.6, Q3 36). Median MCT (mm) medially was 1.8 (IQR 1.8, Q1 0.9, Q3 2.7), laterally 1.8 (IQR 0.9, Q1 0.9, Q3 1.8).
***Conclusions*** No significant differences of measured values were found between the two groups. The proposed radiological criteria are not reliable to anticipate occurrence of PF through SBC.
***Significance*** Ability to predict PFs using simple radiological criteria would help lower the considerable morbidity they cause.

### EP31
### Characterization of Posterior Acetabular Wall Calcification: The Posterior Rim Sign

**William Z Morris**[1], Daniel R Cooperman[2], Raymond W Liu[1]

Case Western Reserve University, Cleveland, USA; 2 Yale University School of Medicine, New Haven, USA

**LEVEL 4/Hip/Lower extremity**
Keywords: Acetabular ossification, Posterior wall, Posterior rim sign.

***Purpose*** A recent study evaluating ossification of the posterior wall of the acetabulum noted a secondary ossification center (the "posterior rim sign") that briefly appears on plain films and MRI. This finding can appear similar to a labral avulsion or traumatic injury to the posterior wall of the acetabulum. This study aims to better characterize this secondary ossification center on plain radiographs in order to allow physicians to more easily recognize this normal finding.
***Methods*** The Brush Inquiry is a collection of radiographs obtained at a minimum annually in a longitudinal study of over 2,800 healthy children between 1926 and 1942. Anterior–Posterior radiographs of the left hip were retrieved for 150 subjects of the study, and 112 (51 females and 61 males) were included in the study as they had at least four consecutive annual radiographs including documented closure of the triradiate cartilage. In total, 612 images were evaluated for appearance and fusion of the posterior rim of the acetabulum.
***Results*** The posterior wall secondary ossification center was identified in 77.4 % of males and 76.9 % of females ($p = 0.475$). The mean age at time of radiographic appearance was 14.1 years of age for males and 12.3 years of age for females ($p < 0.001$). Among those subjects in whom the posterior rim sign was visualized, it was seen on only one image in 86 % of the subjects while in 14 % of subjects the sign was visualized for 2 consecutive annual images. In 96 % of the subjects, the ossification center was visualized the year before or the year of triradiate closure.
***Conclusions*** The appearance of the secondary ossification center of the posterior wall of the acetabulum (the "posterior rim sign") is a common radiographic finding that was visualized in more than three quarters of all patients with no significant difference in the incidence in males and females. This sign presented earlier in females in accordance with the earlier closure of the triradiate cartilage. This radiographic finding is also short-lived and was likely missed in the remaining quarter of patients in serial images that were obtained annually.
***Significance*** The posterior rim sign is a predictable and common finding in adolescents around the time of fusion of the triradiate cartilage. Understanding its timing and appearance can help guide physicians in diagnosis and avoiding unnecessary and costly advanced imaging.

### EP32

### Validity of a Rotational Mechanism for Stable Slipped Capital Femoral Epiphysis

**Raymond W Liu**[1], Sara M Fraley[1], William Z Morris[1], Daniel R Cooperman[2]

Case Western Reserve University, Cleveland, USA; 2 Yale University School of Medicine, New Haven, USA

**LEVEL 4/Hip/Lower extremity**
Keywords: Slipped capital femoral epiphysis, Calcar, Stable slipped capital femoral epiphysis.

***Purpose*** It has been proposed that the mechanism for slipped capital femoral epiphysis (SCFE) may occur through rotation on the eccentrically located epiphyseal tubercle. There is a ridge of bone extending from the lesser trochanter along the postero-inferior femoral neck which corresponds with the femoral calcar. This ridge points directly towards the fovea and appears to be a good marker for epiphyseal rotation. The goal of this study was to better define this ridge, and determine whether it can be used to support a rotational mechanism for SCFE.
***Methods*** The position of the calcar ridge in comparison to the fovea was measured on 11 cadaveric femora with SCFE, 100 control femora, and 25 immature femora ages $10.6 \pm 2.3$ years where the epiphysis was rotated 0°, 30°, 60° and 90° on the epiphyseal tubercle. The perpendicular distance from a line extending along the calcar ridge versus the center of the fovea was measured. For the epiphyseal rotation specimens AP and axial Southwick angles were measured to determine the amount of varus and retroversion deformity.
***Results*** The mean distance from the calcar ridge line to the fovea in SCFE specimens was $23 \pm 10$ mm, and significantly different from the mean in controls ($1 \pm 5$ mm, $P < 0.0005$). With epiphyseal rotations of 0°, 30°, 60° and 90° the distance was $1 \pm 3$, $7 \pm 5$, $13 \pm 6$, and $17 \pm 5$ mm ($P < 0.0005$). For epiphyseal rotations of 30°, 60° and 90° the changes in AP Southwick angles from baseline were $-3.6° \pm 3.2°$, $-1.9° \pm 3.2°$ and $-0.1° \pm 4.7°$ ($P = 0.001$) and the changes in axial plane modified Southwick angles were $-18.8° \pm 6.7°$, $-28.5° \pm 9.0°$ and $-37.8° \pm 9.2°$ ($P < 0.0005$).
***Conclusions*** In normal specimens a line projecting from the calcar ridge approximately bisects the fovea. With SCFE, the fovea is significantly posterior to this line. When the epiphysis is experimentally rotated, the relationship of fovea to the line approaches that of a SCFE specimen, supporting rotation on the epiphyseal tubercle as a mechanism for stable SCFE. Our angular measurements demonstrated minimal varus but significant retroversion deformity with stable SCFE.
***Significance*** Based on a rotational etiology, open treatment of SCFE should include awareness of a possible chronic rotational component to the deformity. In stable SCFE one should be cautious that any suspected varus deformity may be projectional, secondary to external rotation of the hip.

### EP33

### Lengthening For Congenital Limb Length Discrepancy Using the PRECICE™

Kevin Debiparshad, David Chong, **Dror Paley**

Paley Advanced Limb Lengthening Institute, West Palm Beach, USA

**LEVEL 3/Hip/Lower extremity**
Keywords: Congenital limb length discrepancy, Limb lengthening, PRECICE nail.

***Purpose*** Lengthening for congenital limb length discrepancy (LLD) is usually carried out using external fixation which can bridge across the knee and ankle to protect these joints. The PRECICE™ (Ellipse Technologies, Irvine, CA) is a recently FDA approved lengthening nail which can be used in older patients with LLD. The purpose of this study is to review its use in congenital LLD.
***Methods*** The prerequisites were bone length at least 230 mm and diameter allowing reaming to 12.5 mm; growth plates closed for the tibia and growth plates open or closed for the femur. The hip had to be stable, knee and ankle braceable and daily physiotherapy required. Forty congenital limb deficiencies, mean age 18.4 years (10.3–43.7 years) were lengthened by the PRECICE™ device. The pre-operative measured/predicted mean LLD was 5.7 cm (1.5–18.2 cm). There were 34 femoral and 7 tibial lengthenings and one simultaneous femoral and tibial lengthening.
***Results*** The mean lengthening was 4.3 cm. The distraction rate was 0.80 mm/day (0.5–1.07 mm/day), and the mean time for bony healing was 140.7 days (61–262 days). Four patients required a bone grafting procedure. Five patients stopped distraction early due to complications. There were two deep infections requiring surgery. In total there were 12 unplanned surgeries to treat and resolve complications.
***Conclusions*** This study demonstrates that lengthening with the PRECICE™ can be done safely for congenital LLD. Rate control and ability to achieve desired length was excellent. Bracing can protect the unstable knee and ankle from subluxation in most cases. The mechanism of lengthening was reliable in all but one patient. Complication rates were comparable or lower than previous internal or external lengthening devices. Latent intramedullary infection is a risk in patients that had previous external fixation lengthening.
***Significance*** Lengthening for congenital LLD is practical with the PRECICE$^{TM}$ if certain precautions are taken.

### EP34

### Bilateral Oblique Anterior Pelvic Osteotomy in Classic Bladder Exstrophy

**Emmanouil Morakis**, Mohan V. Belthur, Farhan Ali, Raimondo M Cervellione, Tahir Khan

Royal Manchester Children's Hospital, Manchester, UK

**LEVEL 4/Hip/Lower extremity**
Keywords: Pelvic osteotomy, Bladder exstrophy.

***Purpose*** To report our experience in the management of patients with classic bladder exstrophy treated with urologic reconstruction and bilateral anterior oblique pelvic osteotomies.
***Methods*** This is a retrospective study of 35 children treated, between 2007 and 2013. All patients were treated within the first two years of life with urologic reconstruction and primary abdominal wall repair. Bilateral anterior oblique pelvic osteotomies with external and internal (symphyseal sutures) fixation were done to facilitate tension free reconstruction. The average age of the patients at surgery was 5.1 months. There were 13 girls and 22 boys. The average follow up was 35 months. The patients were immobilized post-operatively with traction (15) or mermaid dressings (20) for 6–8 weeks. We measured clinical, radiologic, functional and patient satisfaction outcomes.
***Results*** All pelvic osteotomies healed and the external fixators were removed at 5.6 weeks post-operatively. Duration of hospital stay was 5.9 weeks. The average pre-operative, immediate post-operative and final follow-up pubic symphyseal diastasis was 42, 17 and 45 mm respectively. One patient had a partial wound dehiscence, which required revision surgery. There were no neurovascular, skin or bone complications. Three patients had a superficial pin tract infection that was treated with oral antibiotics. No patient showed acetabular dysplasia at final follow up. No patient had limb length discrepancy.
***Conclusions*** Bilateral oblique anterior pelvic osteotomies are safe and facilitate tension free urologic and abdominal wall closure in children with classic bladder exstrophy in the short and medium term follow-up.
***Significance*** Bilateral oblique anterior pelvic osteotomies are a safe and effective technique to allow the urologic and abdominal wall closure in children with classic bladder exstrophy. Long term follow-up is needed to assess its effect on the urologic and musculoskeletal function.

### EP35

### 11 Years Follow-Up of Congenital Hip Joint Dislocation Treated By Ludloffs Approach

**Jesper Holbeck-Brendel**, Line Kjeldgaard Pedersen, Ole Rahbek, Michel Bach Hellfritzsch, Peter Dalsgaard Nielsen, Bjarne Moeller-Madsen

Department of Children's Orthopedics, Aarhus University Hospital, Aarhus, Denmark

**LEVEL 3/Hip/Lower extremity**
Keywords: Ludloff, Congenital hip joint dislocation.

***Purpose*** To evaluate the clinical and radiographic outcome after treatment of congenital hip dislocation by Ludloffs approach at the Department of Children's Orthopedics, Aarhus University Hospital.
***Methods*** 19 patients underwent Ludloffs approach from 1997 to 2005 and were invited for follow-up. 14 children accepted and a clinical and radiographic examination of the hip and pelvis were performed. In total 17 hips were treated with Ludloffs approach. Radiographic outcome measures were acetabular angle, migration index (MI) and signs of late complications in form of both distinct and subtle signs of AVN. The clinical outcome measures were range of motion (ROM) of the hip joints, individual orthopedic complaints and the HAGOS hip questionnaire.
***Results*** Mean age at surgery was 12 months. Mean follow-up time was 11.2 years. Radiographic evaluation showed signs of AVN in 5 of 17 operated hips (29.4 %). Six operated hips have required additional surgery. A significant correlation was found between both MI and AVN ($p < 0.05$) and follow-up time ($p < 0.01$). Difference in ROM for unilateral treated hips was only significant for flexion ($p < 0.02$). Two of five questions of quality of life in the HAGOS hip questionnaire showed a significant worse outcome for patients with AVN ($p < 0.01$, $p < 0.01$).
***Conclusions*** Ludloffs approach is relevant in the treatment of congenital hip dislocation. This study indicates an acceptable rate of late AVN and a general good clinical and radiographic outcome.
***Significance*** This study provides evidence that Ludloffs approach can be considered a successful treatment option for congenital hip dislocation.

### EP36

### "To Pin or not to Pin'' The Contralateral Hip in Unilateral SUFE

**Harvey L George**, Raj Maheshwari, Ankur Sinha, Rebekah Thorne, Colin Bruce

AlderHey Hospital, Liverpool, UK

**LEVEL 3/Hip/Lower extremity**
Keywords: Prophylactic pinning, Unilateral, SUFE.

***Purpose*** To improve on current criteria for prophylactic pinning of the contralateral side in unilateral SUFE.
***Methods*** Between 2008 and 2013, 68 patients were treated for SUFE in our institution. Detailed demographics, operation notes, follow-up clinical and radiographic findings, complications and secondary surgical procedures were noted. The criteria used for prophylactic pinning were assessed and they were age, severity of slip and endocrine abnormalities.
***Results*** 68 patients were treated for SUFE. 28 patients had bilateral pinning. 14 of them had early prophylactic pinning of the opposite side. None of these patients developed any complications in the prophylactically pinned hips. 14 other patients became symptomatic and had the other hip pinned at a later stage. In this group of patients 3 developed AVN.
***Conclusions*** Among 54 patients who did not have early prophylactic pinning of the contralateral hip, 26 % eventually underwent late contralateral pinning because of the onset of new symptoms. Of these, 20 % subsequently developed AVN. Had these patients been prophylactically pinned the risk and complications of a later contralateral slip might have been avoided.
***Significance*** We discuss whether the introduction of more robust criteria to determine the need for prophylactic pinning such as the use of BMI, type of slip (acute or chronic), stability of slip (stable or unstable), posterior sloping angle together with bone age severity of slip and endocrine abnormalities, might reduce the risk and complications of a later contralateral slip.

### EP37

### Temporary Hemi-Epiphysiodesis With Plates: Comparison of Two Devices

David Chong, Kevin Debiparshad, **Dror Paley**

Paley Advanced Limb Lengthening Institute, West Palm Beach, FL, USA

**LEVEL 3/Hip/Lower extremity**
Keywords: Hemi-epiphysiodesis, Guided growth, 8 plate, Hinge plate.

***Purpose*** Temporary hemi-epiphysiodesis with the 8-plate (Orthofix, Italy) is often referred to as guided growth. The 2 cannulated screws tether the growth of the physis until the screws impinge on the holes of the plate. Breakage of screws is reported to occur related to impingement especially in large patients. In the hinge plate (Pega Medical, Canada), the plate hinge and the solid screws are free to angulate increasing the range without impingement. Theoretically, screw breakage should be less likely to occur. The purpose of this study was to compare hinge plates and 8-plates used for temporary hemi-epiphysiodesis.
***Methods*** 123 patients treated with 170 hemi-epiphysiodeses were reviewed retrospectively: 34 hinge and 136 8-plates. The deformities treated were in the frontal (5°–28°) and sagittal planes (6°–53°). Measurements of the specific joint orientation angles being corrected as well as the mechanical axis deviation and mechanical femoral-tibial angles were recorded before, at removal and at latest followup.
***Results*** There was no significant difference in age, deformity or indication for the two plates. There was no breakage of any screws or plates. There was no significant difference in rate, degree or rebound of correction. Expected guided growth was achieved equally for both plates. The only difference was the conformity of the plate to the surface of the bone. The hinge plate conformed better to the bone contour. It was easier to insert and remove the screws of the 8-plate because they are cannulated.
***Conclusions*** Both 8 and hinge plates are equally effective in correcting angular deformity. The hinge plate may be better suited to non-flat bony surfaces (e.g. medial distal femur). The 8-plate is easier to insert and remove.
***Significance*** Temporary hemi-epiphysiodesis is a good technique independent of the make and design of the plate device.

### EP38

### A Prospective Study of the W/M Serrated Osteotomy for Varus Deformity in Ghanaian Patients, The Preliminary Results of the First 14 Juvenile Blount's Patients

**Heleen M Staal**[1], Joseph Korpisah[2], Freek Hollman[1], Paul J.J.M Rompa[2], Lodewijk W[1]

Maastricht University Medical Centre, Maastricht, The Netherlands; 2. St. Anthony's Hospital, Dzodze, Ghana

**LEVEL 2/Hip/Lower extremity**
Keywords: W/M serrated osteotomy, Juvenile Blount.

***Purpose*** The W/M serrated high tibial osteotomy is a not frequently described surgical technique that offers the opportunity to simultaneously correct the varus and the torsional deformity of the tibia in Blount's disease. This technique normally requires no additional internal fixation but only the application of a cast. Therefore the technique is ideal for the use in developing countries.
So far only one study was published on patients in Western Europe. In a prospective study done in Ghana 48 patients with varus deformity of the tibia were included. Out of this group we selected 20 tibias of 14 patients with juvenile Blount's disease that were operated on with the serrated W/M-osteotomy. The short term results are presented.
***Methods*** Between 2008 and 2011, in St Anthony's Hospital in Ghana, 48 patients were enrolled in a treatment protocol for tibial varus deformities. A W/M serrated osteotomy of the proximal tibia was performed. All patients with juvenile Blount were selected. Preoperatively, patients were clinically evaluated. Pre- and postoperative X-rays were measured: the femoro-tibial angle (FTA), metaphyseal-diaphyseal angle (MDA) and medial physeal slope (MPS). They were classified according to the Langenskiöld classification. Patients were followed for 12 weeks for surgical complications and clinical and radiological outcome.
***Results*** Of 14 patients undergoing bilateral (6) and unilateral (8) procedures 20 tibias were included. The group consisted of 5 males and 9 females, there mean age was 4.7 years (SD 2.21). Langenskiöld classification of the X-rays showed 5 type I, 2 type II, 1 type III, 2 type IV, 4 type V and no type VI. Of the 20 procedures we found only one wound infection. The mean FTA improved from 34.6° varus to 4.8° of valgus. All osteotomies reached full consolidation.
***Conclusions*** The W/M serrated high tibial osteotomy shows good short term results in correcting the deformity of the tibia in patients with juvenile Blount's disease in a developing country. Long term follow-up is needed.
***Significance*** This is the first study on the results of the W/M serrated high tibial osteotomy for juvenile Blount preformed in a developing country, under which circumstances the technique is very suitable but the patient follow-up is challenging.

### EP39

### Natural Course of Tibiofemoral Angle in Excessive Physiological Genu Varum in Children

**Kwang Soon Song**, Si Wook Lee, Hyung Gyu Jang

Department of Orthopedic Surgery, Keimyung University, Daegu, Korea

**LEVEL 3/Hip/Lower extremity**
Keywords: Physiologic genu varum, Genu varum, Tibiofemoral angle, Children.

***Purpose*** This study was performed to identify natural course of tibiofemoral angle (TFA) in children who had excessive physiologic genu varum without any underlying pathologic conditions.
***Methods*** An IRB approved study of 73 limbs (25 bilateral, 23 unilateral) of 48 healthy children (27 male, 21 female) who presented consecutively with excessive physiologic genu varum [equal or above the range of one standard deviation (SD) of anatomic TFA (aTFA)] at initial visit were evaluated until 6 years old without any exception. We divided patients into two groups according to severity (group A: within the range of 1 SD to 2 SD, group B; more than 2 SD. We analyzed the results including value of aTFA at last follow up, changing patterns of TFA with aging, the difference between gender, and also compared our result with those from previous studies in the literature.

***Results*** The mean TFA at initial visit was varus 13° (range 7°–15°) in group A and 17° (range 7°–36°) in group B. All limbs improved spontaneously to within 1 SD of TFA at the last follow up. The mean age of normalization was 25 months in group A and 20 months in group B. (*p* value = 0.410). The patterns of chronological changes of aTFA was similar to previous studies however, duration for normalization of aTFA was prolonged. The mean value of aTFA was 13.4° at 1 year, followed by straightening decrease to 0° at almost 3 year. From the age of 4 year, aTFA was 5.5° of genu valgum and was gradually changed until the age of 6 years, and sustained.
***Conclusions*** An excessive genu varum exceeding 1 SD in young children improved spontaneously without any treatment. Although the physiological genu varum period was prolonged with neutralization of the aTFA (TFA = 0°) delayed by 1.5 year and the definite physiologic genu valgum period was often missing, nevertheless, aTFA became similar to previous reports of 6° valgus at 6 years old at the last follow up.
***Significance*** From our knowledge, there are several reported studies discussing average value and normal range of aTFA in growing children from different races. This study was designed to observe natural course of excessive physiological genu varum (equal or over 1 SD).

### EP40
### A Study of the Validity of Biplanar Imaging for Measuring Femoral Anteversion

**William Z Morris**[1], Havalee Henry[2], Raymond W Liu[1], Jonathan J Streit[1], Richard E Grant[3], Daniel R Cooperman[3]

Case Western Reserve University, Cleveland, USA; 2 Yale University School of Medicine, New Haven, USA; 3 Albert Einstein Medical Center, Philadelphia, USA

**LEVEL 4/Hip/Lower extremity**
Keywords: Anteversion, Neck-shaft angle, Biplanar imaging.

***Purpose*** Femoral anteversion can be difficult to determine intraoperatively, particularly in cases with complicated proximal femoral deformity and cases involving midshaft and distal femoral osteotomy. Although biplanar methodology exists for measuring femoral anteversion, the measurements are generally based on the proximal femur, without consideration for the femoral bow. We designed an anatomical study to test the validity of the Ogata and Goldsand methodology for biplanar imaging.
***Methods*** We measured anatomical femoral version in 35 mature cadaveric femora, specifically chosen to represent a wide range of femoral version. Version was then separately calculated using two methodologies. First, a standard Ogata and Goldsand approach was utilized, with femoral version geometrically calculated after measuring apparent neck-shaft angle and the beta angle, which is the angle between the femoral neck and proximal femoral shaft on a direct lateral view. Second, a modified approach was utilized, using the same geometric formula and apparent neck-shaft angle measurement, but with a modified beta angle between the femoral neck and a line representing the entire femur, passing from the posterior condyles to the posterior aspect of the greater trochanter.
***Results*** Mean anatomical femoral anteversion was 19.6° ± 11.6°. Mean calculated femoral version using standard biplanar imaging methodology was 29.6° ± 13.6°, while mean calculated femoral version using the modified biplanar technique was 21.9° ± 11.9°. Repeated measures ANOVA analysis found an overall statistically significant difference between the three groups ($P < 0.0005$), with pairwise comparisons revealing statistically significant differences between all three groups ($P < 0.02$ or lower for all comparisons). There was higher correlation between measured version and the modified biplanar technique as compared to the standard biplanar technique (intraclass correlation coefficient 0.96 versus 0.69).
***Conclusions*** Standard biplanar imaging techniques do not account for the femoral bow, and can significantly overestimate femoral anteversion. This study demonstrates that if a line is drawn from the posterior distal femoral condyles to the posterior aspect of the greater trochanter for the laterally derived beta angle, femoral anteversion is better approximated. Intraoperatively, we have obtained this line by appropriately positioning a straight rod over the skin under fluoroscopy.
***Significance*** In complex operative cases where biplanar imaging is desired to measure intraoperative femoral version, we recommend a modified lateral view measurement technique, which improves accuracy by accounting for the femoral bow.

### EP41
### Timing of Premature Physeal Closure in Legg-Calve-Perthes Disease

**Kwang-Won Park**, Hae-Ryong Song

Korea University Rare Diseases Institute, Seoul, Korea

**LEVEL 3/Legg-Calve Perthes disease**
Keywords: Legg-Calve-Perthes disease, Premature physeal closure.

***Purpose*** Premature physeal closure of the proximal femoral physis has been reported in Legg-Calve-Perthes Disease (LCPD). However, the timing of its occurrence has not yet been reported. We determined the timing of premature physeal closure in unilateral LCPD with serial radiographic evaluation. Age at the time of premature physeal closure was determined and compared to the contralateral hip. We hypothesized that premature physeal closure results in (1) advanced Herring Classification (2) leg length discrepancy (LLD), and (3) trochanteric overgrowth.
***Methods*** Serial radiographs of 29 patients diagnosed with LCPD were assessed (mean 14.2 years: range 11–18 years). The difference in the timing of physeal closure between the hips was calculated. The involved hip was classified according to Herring classification. The LLD and articulotrochanteric distance (ATD) index at latest follow up was measured. The mean values were calculated and statistical comparison of variables was done using the Fisher exact test.
***Results*** The mean difference of physeal closure at the involved hip compared to the uninvolved was 3.5 years (range 2–5 years). Hip with premature physeal closure was associated with Herring B/C and C ($p = 0.006$) and LLD >1 cm ($p = 0.022$). There was no correlation between trochanteric overgrowth and premature physeal closure ($p = 0.190$).
***Conclusions*** We may expect premature physeal closure of the proximal femoral physis in patients with LCPD to occur 3.5 years earlier than normal hips. Presence of premature physeal closure can be an adjunct in the prognostication of LCPD. Future studies directed on premature physeal closure in LCPD and associated growth disturbance are needed.
***Significance*** These results help us select the proper treatment modality for LLD which could occur in the patient with unilateral LCPD according to their expected timing of premature physeal closure.

### EP42

### Limb Length Discrepancy in Legg-Calve-Perthes Disease

**Daisuke Tamura**, Hidehiko Kawabata

Pediatric Orthopedics Surgery, Osaka Prefectural Medical Center and Research Institute for Maternal and Child Health, Izumi, Japan

**LEVEL 3/Legg-Calve Perthes disease**
Keywords: Limb length discrepancy, Legg-Calve-Perthes disease.

***Purpose*** Severe Legg-Calve-Perthes disease (LCPD) involves not only the epiphysis but the metaphysis and growth plate of the proximal femur. It sometimes induces limb length discrepancy (LLD). Moreover, poor remodeling after collapse of the femoral head or early proximal femoral physeal closure have been thought to increase the risk of LLD. However, what factors may be the cause of LLD remain unclear now. The purpose of this study is to determine the prognostic factor of LLD in LCPD patients.
***Methods*** To clarify this problem, we retrospectively reviewed the medical records of all patients who were diagnosed LCPD at our hospital between 2006 and 2010. There were 86 patients with LCPD and 68 patients were skeletally mature. In these patients, 59 patients had unilateral involvement and were evaluable for this study. All hips were treated by containment methods [abduction brace, abduction cast, operation (varus osteotomy and/or Salter osteotomy)]. Radiographs were evaluated for severity of the disease (Catterall classification and Herring's lateral pillar classification), shape of the femoral head (Stulberg classification) and LLD at the final follow-up. Chi squared tests were used to determine whether potential predictive factors altered the degree of LLD. We also assessed odds ratio for the predictive factors of LLD.
***Results*** LLD was found in 26 patients and ranged from 5 to 41 mm (average 14.8 mm). The severe limb shortening was observed in Catterall group 4 (average 10.8 mm), lateral pillar Group C (average 9.5 mm) and Stulberg class 4 (average 16.1 mm). LLD was also observed in the older patients who underwent an operation. Severity of the disease (Catterall classification and lateral pillar classification), age of onset and operation were associated with LLD in the Chi square test. Hence odds ratios revealed that age of onset (OR 6.8; 95 % CI 1.82–26.1) and operation (OR 3.6; 95 % CI 1.06–12.6) had a strong association with LLD. The statistical analysis revealed that sex was not associated with LLD.
***Conclusions*** The amount of residual shortening of the affected limb in Legg-Calve-Perthes disease at the end of skeletal growth seems to depend on the extent of involvement and the presence of a growth arrest. The extent of femoral head involvement, age of onset (more than 8 years old) and operation, including varus osteotomy for containment therapy were associated with LLD at final follow up.
***Significance*** In this study, we found the extent of femoral head involvement, age of onset and operative treatment were the positive predictors of the LLD with unilateral LCPD. Among them, age of onset was the strongest predictor, whereas extent of femoral head involvement was only weakly associated with LLD.

### EP43

### Quantitative Measure of Lateral Pillar in Legg-Calvé-Perthes Disease

Tohru Futami

Shiga Medical Center for Children, Moriyama, Japan

**LEVEL 3/Legg-Calve Perthes disease**
Keywords: Legg-Calvé-Perthes disease, Lateral pillar, classification.

***Purpose*** The lateral pillar classification is widely used in the treatment of Legg-Calvé-Perthes disease (LCPD). However, the system has the major limitation of being a retrospective and categorical estimation during fragmentation stage rather than a continuous measure. In addition, the reliability of the system seems to be not entirely satisfactory even with the modified Herring classification (Rajan JPO 2013). We attempted to develop a method for quantitative measurement of the lateral pillar height to provide a more accurate prediction of the final sphericity in LCPD.
***Methods*** We retrospectively analyzed radiographs of 90 patients who were treated conservatively between 1980 and 1999. Inclusion criteria were: (1) age at onset >5.0 years, (2) early presentation within 4.0 months after onset, (3) unilateral disease, (4) patients who underwent containment therapy by the unilateral brace, and (5) patients who had regular follow-up until skeletal maturity with sufficient sets of radiographs. The mean age of onset was 7.6 ± 1.7 years (range 5.0–11.6). At the latest follow-up, their ages averaged 17.0 years (range 15.0–25.8). The lateral pillar height was measured using a line tangent to both femoral epiphyses and was compared with the contralateral normal side representing the percent lateral pillar (% LP). The relationship between the final outcome using Stulberg's classification and the lowest % LP during the first 3 years was determined by logistic regression analysis. The reliability of measurements for the % LP was assessed by intraclass correlation coefficients (ICC).
***Results*** Seventy-four of the 90 cases (82 %) had the lowest lateral pillar height between 4 and 16 months after onset. The overall mean % LP decreased to a minimum at 10–12 months after onset. The logistic likelihood ratios for the lowest % LP and age at onset indicated a significant influence on the final sphericity ($p < 0.001$). The analysis also indicated the desired % LP required for good results (desired % LP = onset age × 5 + 20, e.g. if onset age is 7.0 years, the lowest % LP should be >55 for Stulberg I or II). The measurements for the % LP showed high intra- and interobserver reliability with mean ICC of 0.896 and 0.831.
***Conclusions*** The lowest % LP combined with the age at onset can enhance accurate prediction of the final femoral head sphericity (Stulberg I, II or III–V) with 87.8 % accuracy.
***Significance*** Quantitative evaluation of the pillar height can facilitate a continuous measure and detailed analysis of the collapsing pillar. The desired % LP may be a reference value for the patient undergoing treatment.

### EP44

### Correction of Severe Equinus Deformities in Older Children Using Taylor Spatial Frame

**Pavel Kotlyarsky**, Alexander Katsman, Mark Eidelman

Rambam Health Care Campus, Haifa, Israel

**LEVEL 4/Limb reconstruction**
Keywords: Arthrogryposis, Equinus correction, Taylor spatial frame.

***Purpose*** Treatment of rigid equinus deformities might be a difficult and challenging problem. Most patients have already undergone at least one soft tissue release and recurrence of equinus is common. The purpose of this study was to evaluate the results of treatment of severe equinus using the Taylor Spatial Frame (TSF).
***Methods*** 12 patients (4 females and 8 males, mean age 14.1 years) who were treated in our institution from 2005 to 2012 were involved in this study. Five patients had clubfoot, three arthrogryposis, two developmental equinovarus, and two patients had deformities secondary to growth arrest of the distal tibia and extensive deep burns. Mean preoperative dorsiflexion was −15.8° (range 5° to −60°). Soft tissue distraction with standard TSF was done in 10 patients and two patients underwent supramalleolar osteotomy in order to achieve the correction goal (more than 10° dorsiflexion).
***Results*** The correction goal was achieved in 10 patients. In two patients there was a failure of correction with subsequent ankle fusion as salvage procedure. Mean frame time was 91 days (range 62–162), mean postoperative dorsiflexion was +10.4° (range −5° to +20°). At the latest follow-up functional outcome was excellent in two patients, good in seven, fair in two and poor in one patient.
***Conclusions*** Despite severe and rigid equinus, majority of our patients achieved correction goal and good functional results.
***Significance*** Based on our experience TSF is a reliable and powerful tool for correction of rigid equinus in older children.

### EP45

### Percutaneous Epiphysiodesis Using Transphyseal Screws (PETS) in the Management of Leg Length Discrepancy: Optimal Operation Timing and Techniques to Avoid Complications

**Mi Hyun Song**[1], Eun-Seok Choi[2], Moon Seok Park[3], Won Joon Yoo[2], Chin Youb Chung[3], In Ho Choi[2], Tae-Joon Cho[2]

Jeju National University Hospital, Jeju, Korea; 2 Seoul National University Children's Hospital, Seoul, Korea; 3 Seoul National University Bundang Hospital, Seongnam, Korea

**LEVEL 4/Limb reconstruction**
Keywords: Leg length discrepancy, Percutaneous epiphysiodesis using transphyseal screws, Epiphysiodesis.

***Purpose*** Percutaneous epiphysiodesis using transphyseal screws (PETS) has been used to manage leg length discrepancy (LLD) in growing children. It is a minimally invasive and reversible method for the management of LLD. The purposes of this study were to analyze effects of PETS on LLD, its associated complications, and to determine optimal surgical timing and find ways of preventing complications.
***Methods*** Sixty-nine physes (50 distal femur and 19 proximal tibia) of 59 LLD patients (36 boys and 23 girls), who were treated by PETS from August 2005 to January 2011, who had been followed until skeletal maturity or screw removal, and who had not undergone other surgical procedure affecting limb length were subjects of this study. Medical records and plain radiographs were retrospectively reviewed. Retrospective growth calculations were done using the multiplier method. The efficacy of leg length discrepancy correction was calculated, and the predicted segment length with the index operation was compared with the finally measured length. The screw insertion angle in three dimensions was calculated, and was correlated with the efficacy. Complications associated with screw design and the techniques used were analyzed.
***Results*** The LLD correction efficacy averaged 75.5 % (5.0–114.0) at the distal femur and 78.9 % (11.0–111.0) at the proximal tibia. However, the final LLD was less than 1 cm in 89.8 %, and the mean final LLD was 3.0 mm (−10.0 to 16.7), presumably because operations were performed on average 1.3 years earlier than estimated by growth calculation. The mean 3-D screw insertion angle of a physis was 48.4° (42.0–55.5) at the distal femur and 50.7° (45.2–55.7) at the proximal tibia, and the three-dimensional screw insertion angle was positively correlated with LLD correction efficacy. Complications were closely related to the screw design and the implantation techniques. Failure of screw removal was associated with the titanium screw and screws with small heads. Screw dislodgement and subsequent axial deviation was attributable to use of screws with too short thread depth. In addition, axial deviation was also attributed to inadequate purchase of the epiphysis.
***Conclusions*** PETS provides a minimally invasive and effective means of LLD correction. In view of its delayed effect, we recommend that PETS be performed at least 1 year earlier than estimated optimal epiphysiodesis timing. The careful selection of screw design, material, and length, and accurate screw placement are the keys to successful results.
***Significance*** PETS is one of the useful methods of LLD correction, with minimal postoperative morbidity.

### EP46

### Alternative Treatment in Patients With Idiopathic Internal Tibial Torsion by Supramalleolar Derotation Osteotomy of the Tibia Fixed With Locking Plates and Minimal Incisions

**Miguel Angel Galban**, Roceli Villanueva, Adolfredo Santana

Centro Clinico Leopoldo Aguerrevere, Caracas, Venezuela

**LEVEL 3/Limb reconstruction**
Keywords: Internal tibial torsion, Locking compression plate, Supramalleolar osteotomy, Minimal incisions.

***Purpose*** Idiopathic internal tibial torsion is not common in normal children but when it occurs, it can cause disturbances of gait. Flexible flatfoot and shortening of the Achilles tendon often accompany this deformity. In order to treat this a supramalleolar osteotomy of the tibia has been proposed, with or without concomitant fibular osteotomy. The method of fixation has been described with cast, Kirschner wires, Steinmann pins, staples, intramedullary nails, dynamic compression plates and external fixation.
***Methods*** We evaluated 36 patients, 63 tibias with idiopathic internal tibial torsion treated from November 2008 to December 2012. The mean age at the time of surgery was 10.7 years (5–25). All osteotomies were fixed with 3.5 mm straight locked plates and 4 locking screws (2 proximal and 2 distal). We used three minimal incisions, one anterior for proximal screws, another lateral for distal screws, these two incisions of 3 cm, and a third antero-medial incision of

5 mm for the percutaneous osteotomy. The plates were placed under the peroneal muscles and over the periosteum. The osteotomy was done by a percutaneous incision 3 cm above the physis.
***Results*** 62 % of the patients were male. 27 patients presented bilateral deformity and 9 patients had unilateral tibial torsion. The average thigh-foot angle evaluated before surgery was $-16^\circ$ and after surgery the correction gave an average of $+10^\circ$ for the thigh-foot angle. 27 % of the limbs needed lengthening of the Achilles tendon and casting for three weeks. The remaining patients did not use any immobilization and were free to move. All patients were allowed to fully weight bear at three weeks and they started to walk with crutches. Bone healing was obtained in all patients at a mean of seven weeks (5–12). The plates were removed in a period that ranged from 4 to 24 months (mean 10 months). We did not find any screw loosening. Two patients with severe internal rotational deformities needed osteotomy of the fibula, and the decision to do it was intraoperatively. Two patients (4 limbs) had drop foot and paraesthesia over the dorsum of the foot, which recovered totally in a period of 3 weeks, to avoid this, in the last 22 cases we performed routinely a fasciotomy of the anterior compartment, through the same incisions. Another complication we observed was the wound dehiscence in 8 patients, in all cases healed by second intention, and the dehiscences never exceeded 4 mm.
***Conclusions*** The lateral approach with minimal incisions is a safe surgical procedure that allows good coverage of the plate and protects the periosteum of the tibia. Also the fixation is stable enough to allow free movements of the whole limb and permit weight bearing at 3 weeks. The use of a cast was limited to the patients that underwent lengthening of the Achilles tendon. We did not identify any loss of correction at follow up.
***Significance*** Clinical series.

### EP47
### Recurrence of CPT in Adulthood

Christoph Karner, **Franz Grill**

Orthopaedic Hospital Speising, Vienna, Austria

**LEVEL 4/Limb reconstruction**
Keywords: CPT, Pseudarthrosis, Malalignment, Recurrence, Limb reconstruction.

***Purpose*** For the treatment of congenital pseudarthrosis of the tibia, there are several techniques published and in clinical use, mostly with successful fusion of the pseudarthrosis and good clinical results. In literature, an intact tibia at skeletal maturity is counted as a successful outcome but there are almost no reports about long term results and recurrence of pseudarthrosis in adulthood.
***Methods*** Between 1983 and 2013, 55 patients underwent treatment for congenital pseudarthrosis of the tibia at our pediatric orthopedic department. By now, 28 have reached skeletal maturity. In a retrospective analysis, radiographs made during treatment and at skeletal maturity were compared to radiographs made when refracture happened, with focus on location of refractures, present pathology of the fibula, and alignment of the lower limb.
***Results*** In 28 skeletally mature patients, fusion was achieved in 24 cases, 1 patient had to undergo amputation of the lower leg, 1 pseudarthrosis did not heal, 2 patients were lost for follow-up.
6 patients showed recurrence of pseudarthrosis, respectively refracture. In 5 out of 6, the fracture site was located more distally. In all 6 cases, fibula pathology and malalignment of the ankle was present.
***Conclusions*** Fusion at skeletal maturity cannot be accepted as a final outcome of CPT, since refracture can also happen in adult patients. The focus of treatment should not only be directed to fusion, but also to correction of malalignment of the ankle joint and fibula pathology.
***Significance*** Our case series is the first one that defines the long-term sequelae in skeletally mature patients who were assessed as being treated successfully for CPT.

### EP48
### Correction of Complex Tibial Deformities in Children and Adolescents With Double Level Osteotomy and Circular External Fixation

**Amit Sigal**, Alexander Katzman, Mark Eidelman

Rambam Hospital, Haifa, Israel

**LEVEL 4/Limb reconstruction**
Keywords: Bone transport, Tibial osteotomy, External fixation.

***Purpose*** Treatment of complex deformities of tibia is a challenging task. Circular external fixation is the gold standard for bone transport and deformity correction, however all published studies in children deal with one level of correction. The aim of the current study is to evaluate outcomes of double level correction with bone transport in children and adolescents.
***Methods*** Eight patients with complex tibial deformities (4 males and 4 females) with mean age of 14.75 years old (range 8–21 y/o) were studied. All patients had significant shortening with deformities at various levels of the tibia. We performed double level osteotomies in all patients with subsequent deformity correction and bone transport using circular external fixators (Ilizarov/TSF). Five patients had posttraumatic deformities, one hereditary multiple osteochondromatosis (HME), one girl had hip dislocation with severe limb deformities and shortening and one patient had Garre's disease.
***Results*** All patients were corrected precisely and achieved equal length with contralateral side. Mean bone transport was 5.7 cm (range 4–12 cm); mean time in frame was 210.25 days (range 77–391 days). Seven patients have near normal or normal functional status, one patient still has limitation of ankle motion. No one had neurovascular complications associated with treatment. Complications included 3 pin tract infections and one patient developed a growth arrest of the distal tibia several months after removal of fixator.
***Conclusions*** Based on our experience we believe that double level deformity correction with subsequent bone transport is a safe and effective method of treatment of complex tibial deformities in children and adolescents.
***Significance*** Despite complexity of cases successful double level osteotomy with bone transport using circular external fixation might be performed safely in most children and adolescents.

### EP49
### Genu Recurvatum in Adolescents: Reasons, Diagnostics, Treatment

Edgar Bukharev

Turner Scientific and Research Institute for Children's Orthopedics, Saint-Petersburg, Russia

**LEVEL 3/Limb reconstruction**
Keywords: Recurvatum, Deformity correction, External fixation.

***Purpose*** The aims of our study were to determine causes of the genu recurvatum in adolescents, understand how it is possible to prevent this condition and compare two exFix methods of surgical treatment.

***Methods*** We performed a retrospective study of 15 adolescents with genu recurvatum. The average age at the time of surgery was 14.7 years (range 12–17 y). There were 8 female patients and 7 male patients. Four cases were due to deformity secondary to trauma and all had had skeletal traction through the tibial tuberosity, 10 patients had previous hip surgery for DDH and Legg-Calve-Perthes disease with a long plaster cast immobilization, 1 patient had spondyloepiphyseal dysplasia and 1 patient had nonossifying fibroma. All patients underwent X-ray and CT-scan examinations. The cases were divided into two groups according to the methods of surgical treatment. First group (A) patients underwent surgical correction using the Ilizarov technique, for treatment of group B we used computer-assisted Ortho-SUV ExFix Frame.

***Results*** The main reason of genu recurvatum in all patients was asymmetrical growth arrest of the proximal tibial physis. Partial growth arrest was the result of local injury. The average aPPTA was 106° ± 7.8°. The average correction time was 31 ± 4.5 days for group A and 23 ± 3.8 days for group B. ExFix index was 73.9 ± 13.4 and 52.1 ± 14.7 days/cm, respectively. At final follow-up, average aPPTA was 80 °± 2.8° (group A) and 88° ± 9.2° (group B). The accuracy of correction was 94.6 and 74.2 %, respectively.

***Conclusions*** To prevent partial growth arrest and recurvatum deformity we should avoid local injury of the growth plate. The use of Ortho-SUV reduces time for tibia recurvatum deformity correction (by 1.3 times) and provides over 90 % accuracy.

***Significance*** The use of modern computer-assisted external fixators (hexapods) for the correction of deformities can reduce treatment time and helps the patients to return to normal life as soon as possible.

### EP50
### A Reversed “Y” Forearm Bony Reconstruction in Longitudinal Radial Deficiency

**Gheorghe Burnei**, Ileana Georgescu, Stefan Gavriliu, Costel Vlad, Raluca Ghita, Ecaterina Japie

Maria Curie Hospital, Bucharest, Romania

**LEVEL 3/Limb reconstruction**

*Keywords*: Longitudinal radial deficiencies, Forearm lengthening, Radiocarpal neo-joint.

***Purpose*** Manske type IV longitudinal radial deficiencies require orthotic treatment from birth. In time the ulna gets curved and the hand goes into radial deviation, leading to difficulty with function and grip. The forearm is hypoplastic. The aim of this paper is to present this reconstructive procedure and its results.

***Methods*** This retrospective review (2000–2009) analyses 9 cases, 7 boys and 2 girls. Age at surgery: 3 to 9 years (mean 6.2 y). The configuration of a bony skeleton of the forearm with a bifid ulna (reversed Y-shaped) has been performed in 2 stages:

1. Excision of the fibrous anlage and sectioning of tendinous strips at the musculotendinous junctions was followed by the application of an external fixator (EF) and an ulnar corticotomy. Distraction rate: 1/4 mm/8 h. Ulnar lengthening: 4–6 cm.
2. Reconstruction: 1 month after distraction stopped: a proximal fibular graft was fixed next to the distal ulna by a K wire. Casting: 6 weeks to ensure stability and the reversed Y-shaped bony restoration, the hand preserving a normal position.

***Results*** The treatment ensured correction of the ulnar bow, gave a normal hand position, achieved forearm lengthening and a stable bony structure, creating a reversed Y-shaped bifid ulna. A radiocarpal neo-joint was stable but stiff. The ulnar distraction site consolidated in 4.5–5.6 months. Follow-up: 2–9 years (mean 5.2 y). During follow-up the lower levels of the ulna and the fibular graft were constant without length discrepancies. Complications: pin tract infection and distal K wire migration requiring EF removal and a short period of casting (1 case) and retraction of the flexor tendons (2 cases).

***Conclusions*** The distal bifid ulna is a therapeutic alternative to other procedures, ensuring a more stable bony structure. It allows the K wire removal after consolidation of the ulnar-fibular grafting and ulnar lengthening.

***Significance*** Forearm lengthening and curve correction, sustainable over time, may avoid some functional and aesthetic issues or surgical re-interventions.

### EP51
### Can Glucose Level of Joint Fluid Predict Diagnosis of Septic Arthritis?

**Maki Kinugasa**[1], Daisuke Kobayashi[1], Shinichi Satsuma[1], Yoshiyuki Shinada[2]

Kobe Children’s Hospital, Kobe, Japan; 2.Matsudo City Hospital, Matsudo, Japan

**LEVEL 3/Musculoskeletal infections**

Keywords: Septic arthritis, Children, Joint fluid, Glucose level.

***Purpose*** Differentiation between septic and reactive arthritis in children can be difficult. Although the glucose level of the joint fluid has been recognized as a diagnostic aid in arthritis, few reports have been published. The purpose of this study was to investigate the reliability of glucose level of the joint fluid for differentiating septic arthritis from reactive arthritis in children.

***Methods*** We collected data from 20 children with suspected septic arthritis presenting at our institution from 2008 to 2013. All had clinical variables suspicious of septic arthritis. The mean age at the first visit was 3.5 years (range 5 m to 10.5 y), and 15 hips, three knees, one ankle, and one elbow were involved. Arthritis was confirmed by magnetic resonance imaging or ultrasonography. The joint fluid was aspirated at the first visit for bacteriological culture and synovial glucose level determination using a portable glucose meter. When the joint fluid glucose level was less than 40 mg/dl, we performed an arthrotomy and gave intravenous antibiotics. When the glucose level was 40 mg/dl or above, only simple observation was applied. The final diagnosis of true reactive arthritis was assigned when the patient had a negative joint fluid culture or spontaneous resolution of symptoms without antibiotic therapy. The final diagnosis of true septic arthritis was based on a positive joint fluid culture or further development of a disease process without antibiotic therapy.

***Results*** Seven patients had joint fluid glucose levels of less than 40 mg/dl. All seven (100 %) had positive joint fluid cultures and were diagnosed with true septic arthritis. On the other hand, 13 patients had synovial glucose levels above or equal to 40 mg/dl. Three of these (23 %) had positive joint fluid cultures and were diagnosed with true septic arthritis. Univariate analysis demonstrated a significant difference ($P < 0.05$) between 10 patients with true septic arthritis and 10 patients with true reactive arthritis based on the joint fluid glucose level: 36.3 ± 35.0 compared with 90.0 ± 23.05 mg/dl.

***Conclusions*** Our study suggests that patients with a joint fluid glucose level of less than 40 mg/dl had a high risk for true septic arthritis. Further, even if the glucose level is more than 40 mg/dl, we cannot eliminate the possibility of septic arthritis. Careful and repeated observation is needed to avoid overlooking true septic arthritis.
***Significance*** Measurement of the joint fluid glucose level is an easy, convenient, and reliable procedure to help with the differential diagnosis between septic arthritis and reactive arthritis in children.

### EP52
### The Orthopaedic Sequelae of Childhood Meningococcal Septicaemia

**Fergal Monsell**, Tomos Edwards, Fiona Bintcliffe, Lowri Bowen, James Aird

Bristol children's Hospital, Bristol, UK

**LEVEL 4/Musculoskeletal infections**
Keywords: Meningococcal, Septicaemia, Orthopaedic, Sequelae, Limb-reconstruction.

***Purpose*** Meningococcal infection is the most common infective cause of death in children and causes significant morbidity in survivors.
***Methods*** Patients admitted to the Paediatric Intensive Care Unit (PICU) of the Bristol Royal Hospital for Children from 01/01/2001 to 31/12/2012 with a primary diagnosis of meningococcal septicaemia were reviewed
***Results*** 10 (7.7 %) of 130 developed orthopaedic complications. Those affected were significantly younger ($p < 0.05$), remained on PICU for longer ($p < 0.001$) and boys had a greater risk of developing orthopaedic complications (risk ratio 3.1; 95 % CI: 0.69–14.14).
9 patients required an amputation, 16/22 (72.7 %) in the lower limb. Patient requiring amputation had multiple limb involvement. 48 growth plate abnormalities were identified in 8 patients, 39 (81.3 %) in the lower limb, most commonly in the distal tibia. 6 patients developed varus malalignment in 10 ankles due to partial distal tibia arrest with relative fibular overgrowth. 6 patients had significant shortening of the tibia.
***Conclusions*** This study has identified a high incidence of musculoskeletal morbidity. Close surveillance of these patients is recommended to identify growth arrest before the onset of clinically significant deformity.
***Significance*** This study presents outcome in a defined population of patients with meningococcal septicaemia using admission to PICU as an entry criterion. This allows accurate determination of the incidence and characteristics of the skeletal consequences of this condition.

### EP53
### Bilateral Involvement or Concomitant Distant Infections of the Septic Hip in Children

**Kwang Soon Song**[1], Si Wook Lee[2], Dong Wan Kim[1]

Department of Orthopedic Surgery, Daegu, Korea; 2. Keimyung University, Daegu, Korea

**LEVEL 3/Musculoskeletal infections**
Keywords: Septic hip arthritis, Bilateral involvement, Concomitant infection.

***Purpose*** To investigate the incidence of bilateral involvement of hips or concomitant distant infection and evaluate the correlation between these two factors and end result in septic hip in children.
***Methods*** December 1987 to June 2006, 53 cases of acute septic arthritis of hip with a minimum follow up of 3 years were selected and analysed retrospectively. Incidence of consequent involvement of septic arthritis of the opposite hip, other joint or infections on other sites were evaluated. All patients were classified radiologically according to Choi's criteria for evaluation of long-term end result. 1A and 1B hips were classified as good result, whilst Groups 2, 3, 4 were considered as poor result. We divided our patients into two basic groups, group 1 (good result) and group 2 (bad results).
***Results*** We could find 15 of 53(28 %) cases of consequent involvement of the opposite hip or knee with septic arthritis or osteomyelitis of the hand, humerus, ilium, femur, or infection of the urinary tract or combination of these lesions.
6 of all 53 (11 %) cases were associated with septic arthritis of the opposite hip. 2 of 34 (6 %) were in group 1, and 5 of 19 (26 %) in group 2. 11 of 53 (25 %) cases concomitant infection on the other lesion. 6 of 34 (18 %) cases were involved in group 1 and, 5 of 19 (26 %) in group 2. More cases of were involved in group 2. The combined osteomyelitis of ipsilateral proximal femur were also noted in 29 of all 53 (55 %), 15 of 34 (44 %) in group 1, and 14 of 19 (74 %) in group 2.
The mean age of onset in the good result group was 1.7 years (range 2 days to 12.8 years) while in poor result group, it was 0.8 years (range 6 days to 9 years). However, they had no statistical significance and the final result in the neonate population was not poor as compared to the general population.
***Conclusions*** On the basis of our result, in addition to treating the diagnosed septic hip, we should give attention to high possibility of combined infection of opposite hip, other bones and joints which may be affected by septic conditions. Early detection of the concomitant infection followed by prompt treatment will provide a favorable result with reduction of serious sequelae.
***Significance*** Involvement of the septic hip arthritis with opposite hip and other distant infection of septic conditions are strongly related to prognosis.

### EP54
### Comparison of Treatment of Residual Changes of the Shoulder After Brachial Plexus Birth Palsy in Children and Skeletally Mature Patients Using External Rotation Muscle Transfers

**Jiri Chomiak**, Pavel Dungl, Martin OštáDal, Monika Frydrychova

Orthopaedic Department 1st Medical Faculty Charles University and Hospital Na Bulovce, Prague, Czech Republic

**LEVEL 4/Neuromuscular disorders**
Keywords: Brachial plexus birth palsy, Shoulder, Muscle transfer.

***Purpose*** Results of latissimus dorsi and teres major transfers to the lateral side of humerus for residual shoulder deformity were compared in children and skeletally mature patients.
***Methods*** 15 patients (9 children, 6 skeletally mature patients, age 3–30 years, follow-up 1–22 years) were treated between 1990 and 2012 for internal shoulder contracture after C5–C6 lesions (7 patients), C5–7 lesions (5 patients) and C5–C8–T1 lesions (3 patients), respectively. Range of movement, shoulder function in Mallet scoring system and radiographs were assessed.

***Results*** Preoperatively, restriction of external rotation and abduction of the shoulder were comparable in all patients. Retroversions of glenoid were present in 13 patients and glenohumeral subluxations were noted in 2 patients. Postoperatively in subjective assessment 8 patients improved significantly, 6 patients improved partially and one patient did not improve. In the objective assessment, the main observed function, namely active external rotation improved to 20° (standard dev. 14°) in children and to 17° (standard dev. 12°) in skeletally mature patients. The aggregate Mallet score ranged 11–19 points (average 17 points). All patients, except one, improved in 2–8 points (average 5.1 points) and moved to the group III and IV of this classification. One skeletally mature patient did not improve and the gain in Mallet score was 0.2 point only. Concerning the comparison of results of children and skeletally mature patients, the differences of improvement in all particular functions of Mallet score were non significant (p = 0.24–1).
***Conclusions*** Transfer of latissimus dorsi and teres major muscles to the lateral part of the humerus together with lengthening of the pectoralis major and subscapularis muscles increase the external rotation and abduction of the shoulder in patients with non-dislocated glenohumeral joints.
***Significance*** This method offers comparable results in children and skeletally mature patients.

### EP55

### A Radiographic Study of the Distal Femoral Epiphysis

**Cynthia Van Nguyen**[1], Janelle Dee Greene[1], Daniel Roy Cooperman[2], Raymond Weiwen Liu[1]

Case Western Reserve University, Cleveland, USA; 2. Yale University School of Medicine, Cleveland, USA

**LEVEL 3/Other**
Keywords: Distal femur, Epiphysis, Radiographs.

***Purpose*** Previous studies have described the complex undulation pattern in the distal femoral physis. We investigated whether standard radiographs can visualize these landmarks, in order to guide screw placement in the distal immature femur.
***Methods*** We studied 36 cadaveric immature femora in specimens 3–18 years of age. AP and lateral radiographs were obtained with and without markers placed on the major undulations and were analyzed to determine the relative height or depth of each topographical landmark.
***Results*** Physical examination of the specimens confirmed the central peak as the major structure on the metaphyseal side, and the anteromedial and posterolateral valleys as the major structures on the epiphyseal side. The height or depth of the landmarks increased with increasing age ($r^2 = 0.68, 0.62, 0.64$). AP and lateral xrays with markers correlated well with unmarked xrays for the central ridge ($r^2 = 0.93, 0.77$) and anteromedial valley ($r^2 = 0.86, 0.89$). For the posterolateral valley, the marked and unmarked images correlated well on the AP view ($r^2 = 0.83$) but had lower correlation on the lateral view ($r^2 = 0.53$). The correlation between AP and lateral xrays without markers on the posterolateral valley was also decreased compared to the other two landmarks ($r^2 = 0.22$, versus 0.53 for the central ridge and 0.46 for the anteromedial valley).
***Conclusions*** The sizes of the major peak and valleys of the distal femoral physis increase with age. The position of the central ridge and anteromedial valley are reliably seen on AP and lateral radiographs, while the posterolateral valley can be difficult to see on a lateral view.
***Significance*** While the peaks and valleys of the distal femoral physis are generally well visualized on plain AP and lateral x-rays, caution should be taken when placing screws in the posterolateral aspect of the epiphysis, as lateral radiographs do not visualize the undulation well.

### EP56

### Comparison of Orthopaedic Manifestations of Multiple Epiphyseal Dysplasia caused by MATN3 Versus COMP Mutations

**Tae-Joon Cho**[1], Sang Gyo Seo[1], Hae-Ryong Song[2], Hyun Woo Kim[3], Jong Sup Shim[4], Chin Youb Chung[5], Oh-Hwa Kim[6], In Ho Choi[1]

1. Seoul National University Children's Hospital, Seoul, Korea; 2. Korea University Guro Hospital, Seoul, Korea; 3. Yonsei University Severance Children's Hospital, Seoul, Korea; 4. Sungkyunkwan University Samsung Medical Center, Seoul, Korea; 5 Seoul National University Bundang Hospital, Seongnam, Korea; 6. Ajou University Hospital, Suwon, Korea

**LEVEL 3/Other**
Keywords: Multiple epiphyseal dysplasia, MATN3, COMP.

***Purpose*** Multiple epiphyseal dysplasia (MED) is a relatively common skeletal dysplasia mainly involving the epiphyses of long bones. However, it is a genetically heterogeneous group of diseases sharing certain aspects of radiologic phenotype. MATN3 and COMP are the most common causative genes for the autosomal dominant form of MED, of which radiographic findings were reported. Clinical manifestation of and subsequent orthopedic interventions were compared between MED patients caused by MATN3 and COMP gene mutations.
***Methods*** Fifty-nine molecularly-confirmed MED patients were subjects for this study. The MATN3 gene mutation group comprised of 37 patients (9 female, 28 male). The COMP gene mutation group consisted of 22 cases (15 females, 7 males). Medical records and radiographs were reviewed. Symptom level and clinical data at the first presentation and latest follow-up were compared. Hip radiographs were scrutinized to evaluate the hip development as well as occurrence of AVN. Type and purpose of the surgical intervention was categorized and recorded.
***Results*** At first presentation, the mean age was $8.8 \pm 2.8$ years (mean ± SD) in the MATN3 group, and $8.5 \pm 3.5$ years in the COMP group (p = 0.670). The height was significantly lower in the COMP group than in the MATN3 group ($p < 0.001$). Gait abnormalities were significantly different between the groups at first visit (p = 0.041) and at latest follow-up (p = 0.037). At latest follow-up, hip pain (p = 0.084) and limitation of daily activity (p = 0.075) tended to be more frequent in the COMP group. There was no significant difference in the incidence of AVN between the two groups. However, hip dysplasia was more common in the COMP group, with significantly larger acetabular angle (p = 0.037), smaller center-edge angle (p = 0.002), severe Stulberg classification ($p < 0.001$), and smaller femoral head coverage ($p < 0.001$).
***Conclusions*** Clinical manifestations of MATN3-MED and COMP-MED overlap, but MATN3-MED tends to be clinically milder than COMP-MED.
***Significance*** Radiographic differentiation between the two groups and confirmation with genetic analysis will provide useful information for prediction of clinical course of patients with autosomal dominant form of MED.

### EP57

### The Diagnostic Utility of Muscle Biopsy for Suspected Myopathy: a Primer for Orthopedists

**Brian J Deignan**[1], Corrine Link[1], Kenneth J Rogers[1], William G Mackenzie[1], Mihir M Thacker[2]

Nemours Alfred I du Pont hospital for Children, Wilmington, USA; 2. A.I DuPont Hospital for Children, Wilmington, USA

**LEVEL 4/Other**

Keywords: Muscle biopsy, Myopathy, Diagnosis.

***Purpose*** To review the results of muscle biopsies performed at our institution in order to determine the diagnostic yield and hence the utility of performing the procedure. This information can be used to make specific recommendations to an orthopaedic surgeon for when a muscle biopsy may be indicated, what it may diagnose, and the likelihood of obtaining a definitive diagnosis from the biopsy.
***Methods*** The Muscle Histochemistry Lab at our institution has maintained a log of samples dating back to 2000. Data from patients who had a muscle biopsy between 2000 and 2013 was compiled and cross-referenced with those who were also identified in our surgical database by diagnosis and CPT code. Biopsies performed to diagnose a process other than myopathy (i.e. neoplasm or infection) were excluded. Biopsy specimens that were diagnostic were flagged. For biopsies performed after 2000, preoperative and postoperative diagnoses codes were compared to see if the biopsy results either confirmed or changed the suspected diagnosis.
***Results*** There were 583 biopsy specimens that met criteria for analysis. Of these, 234 of the biopsies yielded a molecular diagnosis. Thus, diagnostic yield was 40 % for the entire sample. The three most common diagnoses made were muscular dystrophy (24 %), neurogenic changes (21.8 %), and mitochondrial myopathy (10.3 %). Diagnosis codes were available for the 225 most recent biopsies performed starting in the year 2000. Of this subset, 97 of the biopsies were diagnostic (yield of 43.1 %), 51 of which confirmed the preoperative diagnosis. Thus, when the biopsy was diagnostic, the preoperative diagnosis was correct just 52.6 % of the time.
***Conclusions*** Orthopaedic surgeons are sometimes consulted by their colleagues in genetics or neurology to biopsy the muscle of a patient with a suspected myopathy. The patients in question are often children, with a biopsy requested to assist in diagnosing the cause of motor delay, such as suspected muscular dystrophy or congenital or metabolic myopathy. Based on our findings, the likelihood of making a diagnosis from the biopsy procedure is 40 %. In the instance when the biopsy is diagnostic, it confirms the preoperative diagnosis just half (52.6 %) of the time.
***Significance*** The above information may be useful when discussing risks and benefits of a procedure with a patient and/or parents.

### EP58

### A Prospective Randomized Trial on the Efficacy for Music Therapy on Decreasing Anxiety in Children Prior to General Anesthesia

**Cynthia Nguyen**, Madeleine Larue, Darrell S George, Allison Gilmore, Raymond W Liu

Case Western Reserve University, Cleveland, USA

**LEVEL 2/Other**

Keywords: Music therapy, General anesthesia, Anxiety.

***Purpose*** The operating room can be a frightening environment for pediatric patients. This study investigated whether music therapy can mitigate anxiety in a child undergoing surgery.
***Methods*** Based on a power analysis set at 80 %, 150 children were randomized to music of the child or parents' choice (N = 46), lullaby music (47) or no music (57). A pulse oximeter was used to measure heart rate in the waiting room, first entry into the operating room and just prior to induction. The perception of anxiety by the anesthesiology assistant and nursing circulator were both graded on a four-point scale (not anxious, mildly anxious, anxious, extremely anxious). Ethnicity, involvement of child life, previous anesthesia, use of intravenous sedation, and parent presence in the operating room were recorded and analyzed in the multiple regression analysis.
***Results*** Average age was 9.2 ± 4.0 years, with 79 boys and 71 girls. The average increases in heart rate entering the operating room were 7.4 beats per minute (bpm), 6.1 bpm, and 5.1 bpm for the patient choice, lullaby and control groups, respectively. The average increases from waiting room to induction were 1.7, −0.7, and 1.2 bpm. Only younger age ($p = 0.001$) was associated with higher heart rate changes entering the operating room, while age showed a trend ($p = 0.077$). IV sedation showed a significant beneficial effect ($p = 0.049$) on heart rate change at induction. Grading by anesthesia showed significant but opposite effects with age (more anxiety with older age, $p = 0.023$), and beneficial effects of IV sedation ($p = 0.004$) versus negative effects with parent inductions ($p = 0.045$). Nurse grading showed benefit with IV sedation ($p = 0.041$), and trends towards significance with: age (more anxiety with older age, $p = 0.059$), beneficial effects with child life ($p = 0.074$), and negative effects with parent induction ($p = 0.082$).
***Conclusions*** Music therapy did not show any benefit in alleviating anxiety in children undergoing general anesthesia. IV sedation showed benefit by most measures, as might be expected by its direct anxiolytic properties. Scoring by anesthesia and nursing suggested that both perceived less anxiety in younger children despite higher heart rate increases. Nurses saw a trend towards benefit from child life, and both anesthesia assistants and circulators appeared to perceive negative effects from parent inductions, which was not reflected in heart rates measurements.
***Significance*** Music therapy does not show efficacy to support its routine use in the operating room. Anxiety may be more difficult to gauge in younger children, and the effects of child life and parent inductions may be influenced by observer bias.

### EP59

### Superior Mesenteric Artery Syndrome Following Surgery for Adolescent Idiopathic Scoliosis

Derrick Lam[1], Joel Lee[1], Joyce H Y Chua[2], York Tien Lee[2], **Kevin Boon Leong Lim**[2]

Yong Loo Lin School of Medicine, National University of Singapore, Singapore, Singapore; 2. KK Women's and Children's Hospital, Singapore, Singapore

**LEVEL 4/Spine**
Keywords: SMA syndrome, Spinal fusion, Gastrointestinal, Complication, Ileus.

***Purpose*** Superior Mesenteric Artery (SMA) syndrome is a rare but potentially fatal complication following spinal fusion for scoliosis. Our aims are twofold: to identify clinical features and evaluate their importance in SMA syndrome following posterior spinal fusion (PSF) in Adolescent Idiopathic Scoliosis (AIS) patients; and to recommend a simple approach for the recognition and management of SMA syndrome.
***Methods*** This is an IRB-approved study. Three patients in our series and 11 patients from other published case reports who underwent PSF for AIS were included for study. Patient demographics, clinical presentation, investigations and management were recorded and analyzed.
***Results*** In this combined series of 14 patients (mean age 14.8 years), the main presenting clinical features included vomiting of any kind (92.9 %), abdominal pain/tenderness (57.1 %), abdominal distension (42.9 %), bilious vomiting (35.7 %) and hypoactive bowel sounds (28.6 %). Most patients presented within 2 weeks of surgery (71.4 %). 50 % of patients presented with both vomiting (of any kind) and abdominal tenderness. The number of presenting symptoms appeared to be directly related to the severity of SMA syndrome. Our second patient had intermittent vomiting on POD3, which was initially regarded as insignificant; she was discharged on POD9 and readmitted for recurrent vomiting, during which SMA syndrome was diagnosed. Our first patient presented in the classical fashion. Our third patient had bilious vomiting post-discharge, which started on POD 13 and increased in frequency till re-admission on POD 27.
***Conclusions*** Vomiting and abdominal pain are non-specific symptoms following PSF; differentiating between SMA syndrome and post-operative ileus can be challenging. The highest index of suspicion applies to patients who present within the first week with symptoms of vomiting and abdominal pain.
***Significance*** We propose an algorithm for the management of SMA syndrome, which includes a focused clinical assessment to evaluate for intestinal obstruction followed by an abdominal x-ray and barium contrast study if clinical assessment is positive. An early referral to general surgery should be considered especially for high-risk patients (BMI <5 % percentile, sagittal kyphosis). Early diagnosis of SMA syndrome allows for early intervention, reducing the likelihood of future complications and need for surgery.

### EP60

### Deep Venous Thromboembolism after Pediatric Scoliosis Surgery

**Natasha O'Malley**, George H Thompson, Jochen Son-Hing, Christina Hardesty, Connie Poe-Kochert

Rainbow Babies and Children's Hospital, Cleveland, USA

**LEVEL 4/Spine**
Keywords: DVT, Scoliosis.

***Purpose*** With a recognized increase in the incidence of (DVT) in young patients, especially in those with complex chronic conditions, it is important for patient safety and risk management to identify subgroups that would benefit from prophylactic treatment. Thus, the aim of our study was to assess whether spine surgery in children was associated with an increased incidence of DVT, and if prophylaxis is warranted.
***Methods*** A prospectively and IRB approved, Pediatric Orthopedic Spine database (1992–2012) was reviewed to identify patients who had a DVT post operatively.
***Results*** 1264 patients (856 female, 408 male) with a mean age at surgery of 12 ± 3.54 years (range 0.75–18 years) who underwent spinal surgery (2062 procedures) were reviewed. Four patients had clinical DVT (0.3 %) within 10 weeks of their procedure (range 31–76 days). All were lower limb DVTs. There were no upper limb thromboses or pulmonary emboli. The affected children were 9 to 17 years old; 3 with a diagnosis of neuromuscular scoliosis (1 post-polio and 2 with myelomeningocele) who underwent posterior spinal fusion with segmental spinal instrumentation, and one who had in situ fusion of a grade 3 spondylolisthesis.
***Conclusions*** Despite extensive surgical procedures, our spinal surgery patients have a low rate of clinical DVT, which has diminished over time, with no events since 2005. The decrease in use of sub-clavian/internal jugular or femoral lines, secondary to the increase of peripherally inserted central catheters (PICCs) may be one contributing factor, as is earlier mobilization and implementation of a strict 2 h turning and leg-exercise protocol in the immediate post-operative period.
***Significance*** We conclude that there is no evidence to routinely treat pediatric patients undergoing spinal surgery with thromboprophylaxis.

### EP61

### Sub-Axial Cervical Spine Injuries In Children And Adolescents

**Robert F. Murphy**, Austin R. Davidson, William C. Warner, Derek M. Kelly, David D. Spence, Jeffrey R. Sawyer

University of Tennessee Campbell Clinic Department of Orthopaedic Surgery, Memphis, United States

**LEVEL 3/Spine**
Keywords: Sub-axial, Cervical, Spine, Trauma, Children, Adolescents.

***Purpose*** Limited data exists on pediatric sub-axial cervical spine injuries. The goal of this study was to characterize a large retrospective consecutive series of patients by injury type, neurologic injury, associated non-spine injuries, and treatment.
***Methods*** Medical records of all patients at one institution from 2003 to 2013 were reviewed. Data abstracted included age, injury type (fracture, dislocation, both, pure ligamentous injury), associated injuries, neurologic status, and treatment.
***Results*** 111 patients were identified, and grouped into three age ranges: infant: 0–3 years (2), youth: 4–12 years (13), and adolescent: 13–18 years (96). The most common mechanism of injury was motor vehicle accident (64 %) followed by sports (11 %) and all-terrain vehicle accidents (7 %). Isolated fractures were identified in all infant patients (100 %), and accounted for the majority of injures in youth (85 %) and adolescents (84 %). Multiple sub-axial cervical spine injuries were present in 1 infant (50 %), 2 youths (15 %) and 34

adolescents (35 %). Other non-contiguous injuries in the thoracic or lumbar spine were seen in one infant (50 %), 5 youths (38 %), and 46 adolescents (48 %). Fifty-three (48 %) of patients had an associated non-spine injury: there were 19 chest/abdominal injuries, 19 head injuries, and 31 musculoskeletal injuries. Neurologic deficits were noted in one infant (50 %), two youths (15 %), and 17 adolescents (18 %). Four patients died (1 youth, 3 adolescents). Most patients were treated non-operatively. One infant was treated in a cervical collar (c-collar) and with observation. All 13 (100 %) youth patients were treated in a c-collar. Of adolescents, 66 (69 %) were treated in a c-collar or with observation, 4 (4 %) were treated with halo-vest immobilization, 24 (25 %) underwent operative intervention, and 2 (2 %) with combined halo-vest and surgical treatment.

***Conclusions*** In this largest consecutive series in the literature, isolated fractures account for the majority of sub-axial cervical spine injuries in pediatric patients. One-third of patients present with multiple sub-axial injuries, and half have additional non-contiguous injuries in the thoracic or lumbar spine. Neurologic deficits and associated non-spine injuries are common. Most patients were treated with a c-collar, but adolescent patients were more likely to require surgical intervention. Adolescent patients were also more likely to sustain multiple spine injuries, both contiguous and non-contiguous.

***Significance*** Pediatric sub-axial cervical spine injuries are an indicator of severe trauma and associated injuries are common. An awareness of these injury patterns as well as a high index of suspicion for secondary injuries must be maintained in evaluating and treating children with sub-axial spine injuries.

### EP62
### Lumbopelvic Balance in Spondylolysis Pars Repair

**Anna M Ey Batlle**, Norberto M Ventura Gómez, Imma Vilalta Vidal, Augusto Covaro, Melisa Stitzman

Hospital Sant Joan de Déu, Barcelona, Spain

**LEVEL 3/Spine**

Keywords: Lumbopelvic balance, Spondylolysis, Smiley-face technique.

***Purpose*** To evaluate the results in terms of pain and complications of a technique (Smiley-face) for spondylolysis repair and the effects of the repair on the lumbopelvic balance.

***Methods*** 14 patients with a mean age of 13.4 years (range 10–17 years) were included in the study with an average follow-up of 20.4 months (range 3–45 months). All of them showed no disc disease and Meyerding's grade I or less spondylolisthesis. Patients were treated with "Smiley Face" (Guillet and Petit Spine 1999) pars repair technique by the same spine team. The Visual Analogue Scale (VAS) and the Oswestry Disability Index were used for the assessment of pain and clinical outcome before and after surgery. Plain films and, in some cases, a CT-Scan was performed for the diagnosis of pars defect and healing after surgery. Pelvic Incidence (PI), Pelvic Tilt (PT), Sacral Slope (SS), Lumbosacral Angle (LSA) and Lumbar Lordosis (LL) were measured by two different observers before surgery and at the end of follow-up.

***Results*** Between 2007 and 2011, 14 patients (mean age 13.4 years) were operated for a symptomatic bilateral spondylolysis of L5 in all cases. VAS indicated excellent overall pain control with little or no pain in daily activities (VAS $< 3$). Functional outcomes were assessed via the Oswestry Disability Index (ODI). Mean ODI before surgery was 56 (range 42–65), after follow-up score was 12.7 (range 2–18) indicating good overall results. No residual pain or complications that required secondary surgery were observed. X-rays and in some cases CT-scans were performed to confirm pars healing. Radiographic measures of PI, SS, PT, LSA and LL presurgery were: 62.4; 46; 15.8; 9.7 and 51.1, respectively, and values at final follow-up were: 66.4; 39.3; 23.1; 7 and 49.5 reaching statistical significance the reduction on SS and the augmentation of PT. Paired sample statistical significance is shown in Table 2.

***Conclusions*** Pars repair and reduction of low back pain could be directly involved on lumbopelvic sagittal balance modifications. Consequently, the reduction of SS and LSA indicates a decreased risk of progression in children with open physis (Risser sign $< 5$).

Pars repair with the pedicle screw-rod construct (Smiley-Face) shows excellent clinical results and contributes to improved lumbopelvic balance in paediatric isthmic type spondylolisthesis patients

***Significance*** Study comparing post and after surgery the sagittal balance in repairing spondylolisthesis using the Smiley-face technique.

### EP63
### Management of Children With Complicated Spinal Deformities in Countries With Minimal Resources

Alaaeldin Azmi Ahmad

Annajah Medical School, Ramallah, State of Palestine

**LEVEL 4/Spine**

Keywords: Severe scoliosis, Rib construct, Developing countries.

***Purpose*** It is conceded that complicated spinal deformity in underdeveloped countries presents specific obstacles, but these are often made into "using cheaper screws" and being cost effective. In fact, this has become the main discourse for addressing scoliosis in the global South. It is not simply an extra problem on the margin but a fundamentally different institutional context which presents unique constraints and requires innovative solutions. We can innovate ways to make the surgery have less operative and postoperative demands, or to make those demands easier to deal with GIVEN the adverse institutional context. This is precisely where 4 rib construct comes in as a surgical choice that is used for complicated spinal deformity in developing country with minimal resources for more than 5 years now.

***Methods*** 13 cases presented with severe spinal deformity: all previously surgically treated for scoliosis (7 congenital, 3 iatrogenic, 2 syndromic, 1 idiopathic). 8 of them had other associated problems: 6 with scoliosis, 6 with kyphoscoliosis and 1 with kyphosis. The surgical management before the 4 rib construct were 28 procedures done, 4 posterior fusion with instrumentation, 3 posterior fusion in situ, 2 anterior and posterior fusion in situ, 2 growing rods, 1 VEPTER, 1 posterior fusion and instrumentation with removal of the implants in same day.

***Results*** Mean age at surgery with 4 rib construct was 10.5 years, with a mean follow-up time of 34 months. All were done with 4 rib construct proximally and pedicular or iliac screws distally. The mean preoperative thoracic scoliosis was 83° and became 66°, thoracic kyphosis 113° to 70°, thoracolumbar scoliosis 60 became 40° and thoracolumbar kyphosis 63 became 22°. Complications were detected in 8 cases: 2 infection, 1 proximal hook dislodgement, 1 implant exposure, 2 broken rods, 1 spinal stenosis, 1 death from a non-surgical related cause.

***Conclusions*** A 4 rib construct can be an alternative choice in developing countries

1- No massive bleeding.

2- No dangerous procedures
3- follow-up problems are not life threatening
***Significance*** With this complex and demanding medical condition in developing countries, we think that 4 rib construct may be a good and safe surgical option.

### EP64

### A High Resolution CT Study on the Intra-Operative Three-Dimensional Morphology of Adolescent Idiopathic Scoliosis

**Tom P Schlosser**[1], Marijn Van Stralen[2], Winnie C Chu[3], Tsz P Lam[4], Bobby K Ng[3], Koen L Vincken[2], Jack C Cheng[4], René M Castelein[1]

1. Department of Orthopaedic Surgery, University Medical Center Utrecht, Utrecht, The Netherlands; 2. Image Sciences Institute, University Medical Center Utrecht, Utrecht, The Netherlands; 3. Department of Diagnostic Radiology and Organ Imaging, Prince of Wales Hospital, Chinese University Hong Kong, Shatin, Hong Kong SAR; 4. Department of Orthopaedic Surgery and Traumatology, Prince of Wales Hospital, Chinese University Hong Kong, Shatin, Hong Kong SAR

**LEVEL 2/Spine**
Keywords: Adolescent idiopathic scoliosis, Three-dimensional morphology, High-resolution computed tomography.

***Purpose*** Adolescent idiopathic scoliosis (AIS) is a three-dimensional deformity of the spine and is characterized by lateral deviation, axial rotation and apical lordosis. As a result of the axial rotation of the apical vertebral bodies towards the convexity of the curve, the anterior spinal column is always longer than the posterior elements of the spine. The exact anterior-posterior length difference, however, cannot be determined on conventional two-dimensional radiographs. This is the first study to estimate the intra-operative 3-D morphology of the spine quantitatively in different types of AIS in relation to normal anatomy.
***Methods*** A consecutive series of high-resolution CT scans of AIS patients (slice thickness 0.3 mm), made for CT-guided pedicle screw placement, were included ($n = 77$). Non-idiopathic curves were excluded. Trauma CT scans of twenty-two age and gender-matched controls were used as reference for normal anatomy. The true 3-D morphology of the curves was analysed using semi-automatic analysis software. By delineating the coronal and sagittal orientation of all upper and lower endplates between T1 and S1, transverse sections of each individual endplate were reconstructed and complete 3-D spine reconstructions were produced. Coronal deviation, axial rotation as well as the exact sagittal length discrepancy between the anterior and posterior side of the spinal column were analysed for all curves and junctional segments. Intraclass correlation coefficients for intraobserver reliability were 0.98–1.00.
***Results*** In the control cohort, the main thoracic spine was 4.1 % shorter anteriorly, and the lumbar spine 7.8 % longer anteriorly. All AIS curves (thoracic and thoracolumbar, structural and nonstructural,) were axially rotated and the 3-D length of the anterior side of the main thoracic and lumbar spine was on average 3.8 and 9.4 % greater than posteriorly. The junctional segment between the curves was not rotated and there was no anterior-posterior length discrepancy. Significant linear relations were observed between coronal Cobb angle, axial rotation and the anterior-posterior length discrepancy in the main thoracic curves ($r > 0.729$; $P < 0.001$) and thoracolumbar/lumbar curves ($r > 0.485$; $P < 0.001$). Cobb angle measurements on upright anterior–posterior radiographs correlated with the true 3-D morphology on prone CT scans ($P < 0.001$). Lateral radiographs, however, had no value for prediction of the 3-D morphology of AIS.
***Conclusions*** The results imply that the anterior side of the main thoracic curve in AIS is on average 7.9 % longer anteriorly compared to normal anatomy. Moreover, all AIS curves are rotated into lordosis and the junctional segment between the curves is already kyphotic.
***Significance*** This study provides a better understanding of the true 3-D morphology of AIS.

### EP65

### Ischio-vertebral Dysplasia: a Retrospective Analysis of 30 Consecutive Cases

**Jean-Charles AuréGan**, Thierry Odent, Lotfi Miladi, Philippe Wicart, Jean Dubousset

Department of Pediatric Orthopedics, Necker Enfants Malades Hospital, AP-HP, University Paris Descartes, Paris, France

**LEVEL 4/Spine**
Keywords: Ischio-vertebral dysplasia, Kyphosis, Scoliosis, Halo-gravity traction.

***Purpose*** Ischio-Vertebral Dysplasia (IVD) is a congenital bone dysplasia defined by a vertebral dysplasia combined with an ischio-pubic branch hypoplasia. Despite a dramatic presentation and a spontaneous evolution leading to an important thoracic kyphoscoliosis with potential neurological and/or respiratory complications, its description remains poor. Our purpose was to elucidate the clinical features and to draw treatment guidelines by reviewing all the cases of IVD treated in five specialized institutions.
***Methods*** We reviewed 30 consecutive patients treated between 1959 and 2010. Eleven were evaluated from medical charts, 16 from clinical review and 3 patients were deceased before the beginning of the study. Mean follow-up was 8.3 years (0.5–31 years). Clinical, radiological and therapeutic aspects were collected.
***Results*** There were 20 females and 10 males. Mean age at diagnosis was 4.8 years (0–29 years). Facial dysmorphism, retrognathism, cleft-palate, Pierre-Robin sequence, cervical spine anomalies, hypoplasia of the body of the scapula, eleven pairs of ribs, and coxa valga were regularly reported. All patients had a segmental dysplasia of the posterior aspect of the spine resulting in a severe thoracic kyphoscoliosis in 23 patients. The spinal 3D-CT revealed a characteristic deformity "in siphon" of the spine. Five patients had paraplegia and 2 a respiratory failure at initial presentation. The orthotic treatment had limited effect on curve progression. Twenty patients have been operated on, most of them with a circumferential spinal fusion resulting in 2 respiratory insufficiencies, 4 paraplegias and 1 pseudarthrosis with instrumentation rupture and paraplegia. Retrospective analysis underlined the role of pre-operative distraction (halo gravity or cast) to prevent neurological complications.
***Conclusions*** Ischio-vertebral dysplasia is a poorly recognised condition with a severe and rapidly evolving thoracic spinal deformity. Patients should be recognized early in order to prevent further complications.
***Significance*** Case series. Level of evidence IV.

### EP66

### Mid To Long Term Outcomes Following Pediatric Spinal Fusion In New York State

**Emily R Dodwell**, David M Dare, Alina Neganova, Ting Pan, Daniel Green, John Blanco, Roger Widmann, Stephen Lyman

Hospital for Special Surgery, New York City, United States

**LEVEL 3/Spine**

Keywords: Arthrodesis, Pediatric, BMP, Revision, Complications.

***Purpose*** Spinal instrumentation and arthrodesis are commonly performed in children for a variety of reasons, including scoliosis, spondylolisthesis and trauma. Surgical indications and intra-operative practices have changed considerably over the past 20 years, with the development of growing spine techniques and new augments such as BMP. The aim of the current study was to determine the rates of spinal arthrodesis, revision arthrodesis, BMP utilization and associated complications in New York State over the past 20 years.

***Methods*** Pediatric spinal fusion procedures performed in New York State between 1990 and 2010 inclusive were identified through the Statewide Planning and Research Cooperative System (SPARCS), an administrative database of hospital admissions held by the New York State Department of Health. The yearly rate of primary and revision pediatric spinal arthrodesis was determined. In-hospital and long-term complication rates were calculated. Regression analysis was used to assess how patients that received BMP might differ from those that did not, and whether in-hospital and long-term complications differed based on BMP utilization status.

***Results*** The rate of pediatric spinal arthrodesis in NY state increased steadily, by approximately 50 %, from 10.2/100 000 in 1990 to 16.2 per 100,000 in 2010. The revision arthrodesis rate more than doubled, from 0.3/100,000 in 1990 to 0.8/100,000 in 2010. Factors associated with BMP utilization included being Caucasian ($p < 0.001$), and having private insurance. BMP utilization increased over time from 3.30 % in 2004 to 8.45 % in 2010. The type of admission (emergency, elective, trauma etc.), the child's sex, and pre-existing medical co-morbidities were not related to the use of BMP. Complications did not differ based on BMP utilization status, although there was a non-significant trend for increased wound healing problems in the group that received BMP (3.7 % wound in patients that received BMP, and 2.3 % in patients that did not receive BMP, $p = 0.106$). Medical complications following surgery were significantly greater in the group that did not receive BMP ($p = 0.002$). Death was not associated with the use of BMP. The median length of stay was 5 days for those that received BMP and those that did not.

***Conclusions*** The rate of pediatric spinal arthrodesis in New York State has risen by over 50 %, while the rate of revision spinal arthrodesis has more than doubled over the past 2 decades. This may be related to changing indications for spinal arthrodesis in idiopathic scoliosis and other conditions, as well as development of novel techniques for treating congenital spine anomalies that were previously not amenable to surgical interventions. Socioeconomic status of the patient factors significantly in BMP utilization practices. Length of stay, death rate and complications did not differ based on BMP utilization status. This study was limited by lack of ICD-9 codes differentiating between BMP-2 and BMP-7 sub-types, and lack of information on BMP dosage in the SPARCS administrative database.

***Significance*** Primary and revision pediatric spinal arthrodesis has increased dramatically over the past 2 decades. Although no differences in complications were identified based on BMP utilization status, BMP has only been in clinical use since 2003, thus long-term follow up is not yet available. BMP is currently not FDA-approved for use in children, and is not indicated for most uncomplicated pediatric spinal fusions. Given recent reports of significant complications in adults, the safety, efficacy and effectiveness of BMP in pediatric spinal fusion should be further investigated to develop specific clinical guidelines for its use.

### EP67

### Growth Arrest Following ACL Reconstruction With Hamstring Autograft in Skeletally Immature Patients: A Review of Four Cases

**Roger F Widmann**, Daniel W Green, Robert Marx, Grant D Shifflet

Hospital for Special Surgery, New York, USA

**LEVEL 4/Sport injuries**

Keywords: ACL, Hamstring autograft, Growth arrest, Skeletally immature.

***Purpose*** We report four patients who developed growth arrest following transphyseal ACL reconstruction with hamstring tendon autograft, offer a review of the current literature, and discuss possible explanations for why this happens and which factors might place patients at risk.

***Methods*** A retrospective review of four skeletally immature patients who underwent ACL reconstruction at a mean age of 14.2 years (range 13.5–14.8) and developed growth arrests was performed. All patients had a transphyseal reconstruction with a hamstring autograft. Standard post-operative care was provided including clinical and radiographic follow-up at regular intervals. Clinically significant post-operative physeal arrest was confirmed on MRI or CT scan. Detailed chart review examined demographics, operative variables, and post-operative subjective and objective clinical measures.

***Results*** Average follow-up time from index procedure was 30.8 months (19–47 months). Two patients developed tibial recurvatum; two patients developed genu valgum. Standard radiographs were negative in the two cases of tibial recurvatum. However, CT scan confirmed arrest of the tibial tubercle apophysis in both cases.
MRI three-dimensional growth plate mapping demonstrated complete fusion of the growth plates in one case and a physeal bar representing 7.4 % of the cross-sectional area in another case. Three patients required further surgery. One patient underwent a distal femoral guided growth procedure, two underwent proximal tibial epiphysiodesis, and one patient was skeletally mature at presentation and did not require deformity correction.

***Conclusions*** Growth arrest following ACL reconstruction in skeletally immature patients is a real concern and this report highlights the importance of careful preoperative and postoperative evaluation and discussion with patients and family members. We hypothesized that the genu valgum cases were technique related with the femoral socket drilled via an anteromedial portal causing a greater area of injury to the physis due to greater obliquity. In the cases of recurvatum, rapid growth spurts could have created a tenoepiphysiodesis effect on the tibial tubercle apophysis resulting in premature closure. Careful attention to the physical examination, including assessment of knee hyperextension to identify tibial tubercle growth arrest cannot be overstated. Additionally, we routinely obtain long-leg films prior to performing an ACL reconstruction and then at six and twelve months post-operatively or every six months until the growth plates are closed to assess leg lengths and lower extremity alignment.
***Significance*** This case series adds to the literature supporting the risk of growth arrest following ACL reconstruction and provides clinical recommendations for early identification and treatment of this potentially devastating complication.

### EP68

### Meniscal Injuries in Adolescent Patients With Anterior Cruciate Ligament Tear

Hsuan-Kai Kao Kao

Chang Gung Memorial Hospital, Taoyuan County, Taiwan

**LEVEL 4/Sport injuries**
Keywords: Anterior cruciate ligament, ACL, Meniscus, Meniscal Tear, Adolescent.

***Purpose*** To evaluate the incidence of meniscal injuries in adolescents with ACL tears. We also evaluate the relationship between the time from injury to ACL reconstruction and the incidence of meniscal injuries.
***Methods*** After approval by the institutional review board, the records of adolescent patients who underwent ACL reconstruction between January 2001 and June 2013 were reviewed. The incidence of meniscal injuries at ACL reconstruction was analyzed by Chi square test. Median time from injury to surgery in patients with or without meniscal injuries were analysed by Mann–Whitney *U* test. Injuries were classified as acute if the elapsed time from injury was within 12 weeks and chronic if the elapsed time was longer than 12 weeks. Relationships between the elapsed time and the incidence of meniscal injuries were analysed and these 2 groups were compared by independent t test and Chi square test.
***Results*** Ninety-five adolescent patients who underwent arthroscopic ACL reconstruction were identified. The mean age at ACL reconstruction was 15.8 years. The mean elapsed time from injury to surgery was 30.4 weeks. The incidence of meniscal injuries was 66.3 %, including medial meniscal tears in 17 patients (17.9 %), lateral meniscal tears in 33 patients (34.7 %), and both meniscal tears in 13 patients (13.7 %). Forty patients were treated within 12 weeks from injury and the other 55 were treated after 12 weeks. Chronic ACL injury was not associated with a greater incidence of meniscal tear.
***Conclusions*** The incidence of meniscal injury was high (66.3 %) in adolescent patients with ACL tears. Longer elapsed time from injury to surgery was not associated with an increase in medial or lateral meniscal tears. The study results did not support early ACL reconstruction in order to prevent subsequent meniscal injuries.
***Significance*** A longer elapsed time from injury to surgery was not associated with an increase in medial or lateral meniscal injuries. Our study did not support early ACL reconstruction in order to prevent subsequent meniscal injuries. The ACL reconstruction could be delayed to minimize the risk of growth disturbance without increasing the incidence of meniscal injuries.

### EP69

### Anatomical Risk Factors for Anterior Cruciate Ligament Tears in Children: A Case-Control Study of Radiological Parameters Describing the Intercondylar Fossa Anatomy

**Joseph Vincent Fournier**, Benjamin Freychet, François Bergerault, Benoit De Courtivron, Christian Bonnard

Service de Chirurgie Orthopédique Pédiatrique, Hôpital Clocheville, CHRU Tours, Tours, France

**LEVEL 3/Sport injuries**
Keywords: ACL tears, Intercondylar fossa, MRI.

***Purpose*** While external factors can account for the increasing incidence of anterior cruciate ligament (ACL) tears in children, structural risk-factors are largely unknown in children. Anatomic variation of the intercondylar fossa could explain some ACL tears. Intercondylar notchplasty is frequently performed during ACL reconstruction to limit anterior conflict. The notch width index (NWI) has been described in adults to measure the shape of the intercondylar fossa. A narrow intercondylar fossa, characterized by a lower NWI is recognized as a risk factor for injury in adults. Other anatomical variations, considered to create an anterior conflict have also been suspected. We aimed to assess the anatomy of the intercondylar fossa to discover risk factors by studying the NWI and the anterior conflict as risk factors for ACL tear in children.
***Methods*** This is a case control study of knee MRIs. Inclusion criteria for the case group were patients who had undergone ACL reconstruction following traumatic ACL tears with at least one open knee physes, and who had had an MRI of the knee prior to surgery. Exclusion criteria were agenesis of the ACL, existence of a systemic connective tissue disorder, or past history of tibial spine fracture. Controls were age matched patients having had a knee MRI interpreted as within normal limits. All measures were performed on a fat sat MRI sequence. NWI consisted of the ratio of the maximum width of the femur epiphysis and the intercondylar notch measured on the same line on a coronal plane. Two additional measures were made to assess for an anterior conflict. Both were performed in the sagittal axis, with the knee at 0° extension. The first one measured the angle of the Blumensaat line and the tibial axis (BT angle). The second one measured the ratio (R) of the two segments of the line extending from the most anterior part to the most posterior of the spinal surface and its intersection with the Blumensaat line. Statistical analyses were performed using T-test and U Mann–Whitney. P level was set at 5 %.
***Results*** 49 patients were included in the case group, 33 males and 16 females, with a mean age of 13.6 years (10–17). Control group consisted of 18 males and 32 females, with a mean age of 13.8 years. The NWI was found to be statistically lower in the case group at 0.244 (SD of 0.02) vs 0.2263 (SD of 0.02). The BT angle was found to be statistically lower in the case group 138.74 (SD of 4.6) vs 141.30 (SD of 7.9) Study of the R ratio showed no statistical difference.
***Conclusions*** A lower NWI is correlated with ACL tears in children. A narrow notch could be a risk factor for ACL tears. Anterior conflict as shown by a lower BT angle could also be a risk factor by creating conflict between the ACL and the femur in extension.
***Significance*** Intercondylar notchplasty, performed at the time of ACL reconstruction is an important part of the reconstruction procedure and could be helpful in preventing iterative rupture of the ACL graft.

### EP70

### Physeal-Sensitive MRI Analysis Shows That an All-Inside, Anterior Cruciate Ligament Reconstruction in Skeletally Immature Athletes is Safe to the Physis

**Danyal H. Nawabi**, Kristofer J Jones, Moira M Mccarthy, Hollis G Potter, Daniel W. Green, Frank A Cordasco

Hospital for Special Surgery, New York, USA

**LEVEL 2/Sport injuries**
Keywords: ACL, Knee, Pediatrics.

***Purpose*** Anterior cruciate ligament (ACL) reconstruction in skeletally immature patients poses a potential risk of growth disturbance due to iatrogenic physeal injury. Multiple physeal-sparing techniques have been described but none to our knowledge combine the benefits of anatomic reconstruction and socket fixation, without violation of either the femoral or tibial physis. The purpose of this study was to evaluate the safety and efficacy of an all-inside, ACL reconstruction technique in skeletally immature athletes.
***Methods*** Twenty-three skeletally immature patients (mean chronologic age 12.6 years, [range 10–15]) were prospectively evaluated following an all-inside ACL reconstruction utilizing hamstring autograft. The mean bone age (Greulich and Pyle method) was 13.5 years. There were 8 females and 15 males. Fifteen patients had an all-epiphyseal (AE) ACL reconstruction and 8 patients had a partial transphyseal (PTP) ACL reconstruction, which spared the femoral physis but crossed the tibial physis. The PTP option was reserved for patients with minimal proximal tibial growth remaining. The mean duration of residual distal femoral growth for patients in the AE group was 2.7 years compared to 1 year in the PTP group. At a mean follow-up of 12.6 months (range 6–24), graft survival, growth arrest, and the amount of physeal violation was quantified using a previously-validated model obtained from 3-D fat suppressed spoiled gradient-recalled echo (SPGR) MRI sequences. Angular deformity and leg length discrepancy were evaluated using full-length standing radiographs.
***Results*** Minimal post-operative tibial physeal changes were seen in 10 of 15 patients in the AE group and by definition; all patients in the PTP group. The mean area of tibial physeal disturbance (±SD) was $42.4 \pm 38.6$ mm$^2$ (mean 1.7 % of total physeal area) in the AE group compared to $216.7 \pm 129.1$ mm$^2$ (mean 7.3 % of total physeal area) in the PTP group ($p = 0.003$). Minimal post-operative femoral physis encroachment was seen in one case of both groups resulting in a mean physeal disturbance of 1.5 %. No cases of growth arrest, tibial fracture, articular surface violation or avascular necrosis were noted on MRI in either group. Graft survival was 100 % amongst the entire cohort. The mean leg length discrepancy was −0.7 mm and there were no cases of angular deformity.
***Conclusions*** Our data suggests all-inside ACL reconstruction is a safe and effective technique for skeletally immature athletes at short-term follow-up. Physeal-specific MRI reveals minimal growth plate disturbance following AE reconstruction relative to a PTP technique; however, both techniques reveal no evidence of significant complications.
***Significance*** Our data suggests all-inside ACL reconstruction is a safe and effective technique for skeletally immature athletes at short-term follow-up. Physeal-specific MRI reveals minimal growth plate disturbance following AE reconstruction relative to a PTP technique; however, both techniques reveal no evidence of significant complications.

### EP71

### Comparison Between Knee Arthroscopy and Ultrasonography in Adolescents

**Tomasz Jaroslaw Rawo**, Marek Trams, Mariusz Chomicki, Andrzej Czop

Szpital Dzieciecy im. prof. Bogdanowicza SPZOZ, Warsaw, Poland

**LEVEL 1/Sport injuries**
Keywords: Ultrasonography, Arthroscopy, Knee disorders.

***Purpose*** The aim of our study is to consider the accuracy of ultrasound for pre-operative assessment of youths with internal knee disorders.
***Methods*** Our study group consists of 21 (9 girls, 11 boys) adolescents, who had an arthroscopy and ultrasonography in 2012. Median age was 14.5 (13–17) years. Isolated disorder of one knee structure was present in 7 cases, 14 cases had complex disorders of more than one internal knee structure. Method was an ultrasonographic assessment of 7 parameters in 6 projections of 21 patients. Ultrasound has been performed by one and always the same physician, who had no knowledge of diagnosis, medical history nor physical exam. Arthroscopy has been performed by another, but always the same physician, who had no knowledge about the outcome of ultrasonography.
***Results*** Outcomes of ultrasonographic and arthroscopic assessment have been compared. Accordance has been reached in 115 parameters (78.2 %). 32 (21.8 %) have been discordant. Sensitivity of ultrasonography in comparison to arthroscopy was 0.78 (78.3 %), accuracy was 0.79 (79.3 %).
***Conclusions*** Ultrasonography is an accurate and suitable tool in assessment of adolescent knee disturbances, such as synovial pathologies, patellar dislocation and ligaments.
***Significance*** We believe that our study will encourage orthopaedic surgeons to use ultrasonography by themselves in preoperative assessment of patients.

### EP72

### Arthroscopic Resection of the Lower Patellar Pole in Adolescents Sportswomen/Men With Chronic Patellar Tendinopathy

Sandor Roth

KBC Rijeka, Rijeka, Croatia

**LEVEL 3/Sport injuries**
Keywords: Patellar tendonitis, Arthroscopy, Sport injury.

***Purpose*** The aim was to compare clinical results of resection of the lower pole of the patellar in adolescent athletes with chronic Jumper's knee by the arthroscopic and the open approach.
***Methods*** From 2006 to 2010, we did 20 resections of the lower pole of the patella in 18 patients (14 F, 4 M), 8 patellae were done with an open approach, and 12 with an arthroscopic approach. The average age of the patients undergoing arthroscopic treatment was 15.3, and open 15.6. All patients were initially treated non-operatively and we used Martens' treatment program. If non-operative measures failed in Blazina stage 3 we used surgical treatment. The mean time of the conservative therapy before arthroscopic treatment was 14 months, and in open 20 months. Postoperative average follow-up in arthroscopic/open approach was 48/56 month.
***Results*** Preoperatively and postoperatively, for clinical evaluation of the results we used the Kujala score, the Lysholm score, and VISA questionnaire. In both groups we found significant improvement of the score in all three questionnaires. We were getting better results with longer follow-up time with open surgery. Postoperative treatment was shorter with arthroscopic approach, with considerably smaller scars and faster recovery.
***Conclusions*** In our experience it is proved that the arthroscopic resection of the lower pole of the patella as a minimally invasive method provides better clinical results to treat the jumper's knee in adolescents.
***Significance*** The return to the sports activity was faster than in the surgical approach. Atypically, we found that in this age group that girls were treated surgically three times more commonly than boys.

### EP73

### The Effect of Regional Trauma Networks on Paediatric Trauma Care in an Integrated Adult Service

**Robert Jordan**, Daniel Westacott, Giles Pattison

University Hospital Coventry and Warwickshire, Coventry, UK

**LEVEL 4/Trauma—Lower limb**
Keywords: Trauma, Resources, Trauma network, Major trauma centre.

***Purpose*** Regional trauma networks have been implemented in an attempt to improve trauma care. However the low volume of paediatric trauma and the geographical variation in children's services provide unique challenges for resource allocation. Our study analyses the impact of becoming a major trauma centre (MTC) on paediatric trauma workload.
***Methods*** We retrospectively reviewed all paediatric 'Trauma calls' admitted between 1st April 2010 and 31st March 2013. As our centre became a major trauma centre on 1st April 2012 our study population was split into 'pre-MTC' and 'post-MTC' groups. Patient demographics, mechanism of injury, patient outcome, Injury Severity Score and radiological investigations were recorded.
***Results*** There were 132 paediatric trauma calls with a 72 % annual increase post-MTC. More children with minor injuries, according to the Injury Severity Score, were seen post-MTC (47.5 versus 29.6 %). Although the proportion of patients undergoing a CT scan remained static, the actual number increased and a higher proportion were normal in the post MTC group (72.9 versus 52.4 %). This contributed to a higher proportion of patients being discharged home directly from the emergency department post MTC (47.5 versus 36.6 %).
***Conclusions*** The implementation of a regional trauma network has led to a rise in the number of paediatric trauma cases. Paediatric trauma calls tend to be less severely injured but the proportion undergoing CT scans has remained the same and these scans are more likely to be normal.
***Significance*** This data illustrates the change in workload associated with the implementation of Regional Trauma Networks. This change will have direct implications for allocation of resources and workforce.

### EP74

### Pediatric Musculoskeletal Injuries Associated With Recreational Motorized Vehicle Use: Do More Wheels Mean a Safer Ride?

Grant D Hogue[1], Robert Lane Wimberly[2], Ryan A Rose[1], **Anthony Ian Riccio**[2]

University of Texas Southwestern Medical Center, Dallas, USA; 2. Texas Scottish Rite Hospital for Children, Dallas, USA

**LEVEL 3/Trauma—Lower limb**
Keywords: All terrain vehicle, Motor bike, Extremity trauma, Protective equipment.

***Purpose*** To identify differences in musculoskeletal injury location, severity, and associated trauma between pediatric operators of all-terrain (ATV) and motorized two-wheel (motor-bike) vehicles.
***Methods*** An IRB approved retrospective review of all ATV and motor-bike related emergency department (ED) visits to a single level 1 trauma center over a 6 year period was performed. Details of all musculoskeletal injuries were documented. Isolated soft tissue injuries and strains were excluded. The use of protective equipment, injury severity score (ISS), length of hospital stay (LOS), associated injuries and demographic data were noted. Statistical analysis using the Student's t-test was performed to identify differences in injury characteristics between ATV and motor-bike riders.
***Results*** 334 ED visits resulted from ATV related trauma with 156 orthopaedic injuries identified in this cohort. 170 ED visits resulted from motor-bike related trauma, accounting for 123 orthopaedic injuries. The percentage of musculoskeletal injuries associated with motor-bike related ED visits (72 %) was significantly higher than that associated with ATV related visits (47 %) [$p < 0.001$]. While lower extremity injuries predominated over upper extremity trauma in both groups, motor-bike riders sustained a significantly higher rate of lower extremity injuries than ATV riders ($p = 0.0003$). Furthermore, motor-bike riders sustained a significantly greater number of tibia/fibula ($p = 0.03$) and wrist ($p = 0.03$) injuries. While a greater proportion of motor-bike riders used protective equipment ($p < 0.0001$), this did not result in a lower ISS, LOS or a diminished incidence of extremity trauma.
***Conclusions*** Children injured while riding motor-bikes are more likely to sustain musculoskeletal trauma than their counterparts using all terrain vehicles, despite a higher rate of protective equipment use. Lower extremity injuries and fractures about the leg and wrist occur with greater frequency in pediatric motor-bike riders.
***Significance*** As musculoskeletal trauma is exceedingly common in children injured while operating recreational motorized vehicles, orthopaedic surgeons should be aware that injury characteristics vary with vehicle type. While the importance of protective equipment use cannot be overemphasized, modifications to existing equipment may allow for better protection of the most common locations of injury.

### EP75

### Outcomes And Complications Of Tibial Tubercle Fractures Within The Pediatric Population. A Systematic Review Of The Literature

Juan Pretell-Mazzini[1], **Derek M. Kelly**[1], Jeffrey R. Sawyer[1], Alice A.[2], David D. Spence[1], William C. Warner[1], James H. Beaty[1]

University of Tennessee/Campbell Clinic Department of Orthopaedic Surgery, Memphis, United States; 2. Le Bonhuer Children's Hospital, Memphis, United States

**LEVEL 3/Trauma—Lower limb**
Keywords: Tibial, Tubercle, Fractures, Pediatric, Systematic, literature, Review.

***Purpose*** The purpose of this study was to perform a comprehensive review of the literature to determine the overall evidence on associated injuries, functional as well as radiological outcomes, method of treatment, and complications when treating tibial tubercle fractures within the pediatric population.
***Methods*** A systematic review of the English literature from 1970 until 2013 was undertaken, comprising 23 eligible articles (Figure 1) reporting on 336 fractures with a mean follow up of 33.56 months (5.7–115). Fracture types were classified as: Type I (fracture of the secondary ossification center near the insertion of the patellar tendon), type II (fracture propagates proximal to the junction with the primary ossification center), type III (fracture extends posteriorly to cross the primary ossification center), type IV (fracture through the entire proximal tibial physis with posterior component), type V (periosteal avulsion of the extensor mechanism from the secondary ossification center or Type IV with intra-articular involvement-"Y" type fracture) and two modifiers: "A" (Patellar/quadriceps tendon avulsion) and "B" (meniscal tears). Clinical outcomes were assessed by the rate of return to pre-injury activity and knee ROM. Rate of fracture healing, associated injuries (patellar/quadriceps tendon avulsion and meniscal tears), compartment syndrome and complications were also reported.
***Results*** Mean age at surgery was 14.6 years. and the most common fracture reported was type III (51 %). The overall associated injury rate was 4.1 % being more common in type III fractures (5 %). Compartment syndrome was present in 4 % of cases. ORIF was performed in 98 % of cases and arthroscopic assistance was reported in 10 cases. Rates of return to pre-injury activity and knee ROM were 98 and 98 %, respectively regardless of the type of fracture ($p = 0.55$). Fracture consolidation was achieved in 99 % of cases. Overall complication rate was 28 %. Removal of hardware due to bursitis (56 %) was the most common complication. Tenderness/prominence (18 %), re-fracture (6 %), and recurvatum (4 %) were also present.
***Conclusions*** Treatment of tibial tubercle fractures produced good clinical as well as radiological results regardless of the type of fracture. Awareness of associated injuries and compartment syndrome is justified. Complications are common without significant effect on final outcomes.
***Significance*** Good results regardless of the type of fracture can be achieved. Complications could be more common than expected.

### EP76

### Outcome of Displaced Distal Tibial Metaphyseal Fractures in Children Between Six and Fifteen Years of Age Treated by Elastic Stable Intramedullary Nails

**Mattia Cravino**[1], Federico Canavese[2], Antoine Samba[2], Vincenzo De Rosa[3], Lorenza Marengo[1], Antonio Andreacchio[1]

Pediatric Orthopedic Department, Regina Margherita Children's Hospital, Torino, Italy; 2 Pediatric Orthopedic Department, University Hospital Estaing, Clermont-Ferrand, France; 3. Pediatric Orthopedic Department, Bellinzona e Valli Regional Hospital, Bellinzona, Switzerland

**LEVEL 4/Trauma—Lower limb**
Keywords: Distal tibia fracture, Fracture, Tibia, Elastic stable intramedullary nails.

***Purpose*** The main objective of this study is to retrospectively evaluate the clinical and radiographic outcomes of displaced distal tibial metaphyseal fractures in children treated by elastic stable intramedullary nails (ESIN).
***Methods*** From June 1995 to August 2012, 18 children were treated surgically using ESIN for displaced closed fractures of the distal metaphysis of the tibia. The patients were followed on a regular basis until union was achieved both clinically and radiographically.
***Results*** Thirteen boys and five girls with a mean age at trauma of $11 \pm 2.9$ years (6–15) were included in the study. Radiographically, all fractures healed without evidence of delayed union, re-fracture or hardware migration. All patients were pain free at last follow up and all regained full, normal activities including sports.
***Conclusions*** This study showed good functional and radiological results in the pediatric population who had sustained closed, traumatic, displaced fracture of the distal tibial metaphysis with or without associated fibula fracture, without neurovascular insult, treated surgically by ESIN. Results were considered good for children aged 6–15 years. Severely displaced distal tibial metaphyseal fractures with associated fibula fracture close to growth plate require stabilization of the fibula as well. Post-operative cast immobilization is recommended in order to prevent a potential risk of secondary displacement.
***Significance*** We believe that ESIN technique is a useful tool in the armamentarium of the pediatric orthopedic surgeon dealing with patients with displaced distal tibial metaphyseal fractures requiring surgical treatment. It results in good clinical and radiological outcomes. It is also reliable and easy to perform.

### EP77

### Sternoclavicular Capsular Ligamentoplasty in the Treatment of Sternoclavicular Dislocation in Older Children and Adolescents: Long Term Results

**Gheorghe Burnei**, Stefan Gavriliu, Costel Vlad, Ileana Georgescu, Ecaterina Japie, Raluca Ghita

Maria Curie Hospital, Bucharest, Romania

**LEVEL 3/Trauma—Upper limb**
Keywords: Sternoclavicular dislocation, Capsular ligamentoplasty, Sternocleidomastoidian Slip.

***Purpose*** In 1986 we performed the first sternoclavicular capsular ligamentoplasty in a 12 years old child with an operated and relapsed sternoclavicular dislocation, by a technique described by Clement Baciu in 1981. The lesion is extremely rare in paediatric pathology, the literature presenting usually the displacement of the external part of the clavicle (Salter-Harris 1 and 2 fractures). Our aim is to evaluate long term results in patients with relapsed, irreducible and old sternoclavicular displacements, where we performed capsular ligamentoplasty with a slip of the sternal head of the sternocleidomastoid muscle.
***Methods*** We operated on 9 patients aged from 10 to 19 years old (between 1986 and 2001) with a mean age of 14 years and 10 months. The follow-up period ranged 12–27 years, averaging 18.5 years. Sex ratio was 5 boys/4 girls. 4 patients presented displacement after failed orthopaedic treatment, 2 after failed fixation with K wires and 3 non-operated, but irreducible. The lesion occured in 6 patients after polytrauma and in 3 it was isolated. In 5 cases the displacement was anterior, in 3 posterior and in one case it was cranial. Surgery intended to rebuild the intraarticular ligament and the anterior capsule by a slip of the sternal head of the sternocleidomastoid muscle preserving the sternal insertion. Plaster cast immobilization was applied for 3 weeks.
***Results*** The joint was stabilized after surgery. In 2 cases the tearing of the tendinous slip was fixed by a 3 weeks transarticular wire to avoid relapse. Recovery started 4 weeks later. One year after surgery 6 patients out of 9 could perform tractions and 3 climbing. Nowadays all are adults. 3 years after surgery all 9 patients are performing sport and daily activities without problems. 2 patients present weather related sensibility and present a limited elevation of the involved clavicle.
***Conclusions*** Sternoclavicular capsular ligamentoplasty offers stability to the reduction, avoids recurrence and allows the desired physical activities to be performed.
***Significance*** Even if rare in children and teenagers, sternoclavicular displacements in severe polytrauma are irreducible and require a simple and effective therapeutic solution ensured by the capsular plasty with a slip from the sternocleidomastoid muscle.

### EP78

### Long Term Follow Up of Conservative and Surgical Treatment Effectiveness and Radiological Changes of Displaced Supracondylar Humeral Fractures in Children

**Emilis Cekanauskas**, Ali Khalil Chahine, Ramune Degliute-Muller, Jolita Gintautiene, Mindaugas Gruzauskas, Rasa Simonaityte

Hospital of Lithuanian University of Health Sciences Kaunas Clinics, Kaunas, Lithuania

**LEVEL 3/Trauma—Upper limb**
Keywords: Supracondylar humeral fracture, Percutaneous pinning, Nerve injury.

***Purpose*** Estimate effectiveness, late changes in function of elbow joint, ROM, radiological changes, cosmetic arm deformities and quality of life in conservative and surgical treatment of supracondylar fractures.
***Methods*** Retrospective study was performed in a single pediatric orthopedic center, following conservative and surgical treatment in children with type II and III (GC) displaced supracondylar humeral fracture (DSCHF) between 2000 and 2008. The patients were divided in two groups according treatment method applied:
–The first group (conservative treatment): closed reduction and immobilization. One patient in this group was treated by skeletal traction ($n = 59$).
–The second group (surgical treatment): percutaneous pinning with K wires (PPKW) (cross pinning)—or open reduction and internal fixation (ORIF) ($n = 110$).
***Results*** DSCHF were diagnosed more in male subjects (61.54 %), than female (38.46 %). Type III DSCHF according GC was diagnosed more frequently: 88.76 % vs type II 11.24 %. Extension type DSCHF dominated: 95.86 %. Left arm fracture occurred in 55 % of all cases. 58 (34.32 %) were treated using closed reduction, 1 (0.59 %) had skeletal traction as the final treatment. 110 had surgery: 99 (58.58 %) had PPKW and 11 (6.51 %) an ORIF. Mean age at injury was $6.52 \pm 2.69$ years (range 1–16 years). The mean follow up was $3.56 \pm 2.36$ years. Neuropathies were diagnosed in 26 (23.64 %) cases in surgery group: 16 (14.55 %) traumatic nerve injuries and 10 (9.09 %) iatrogenic, while in 3 (5.08 %) cases in conservative group. These results were statistically significant ($p < 0.005$). Flexion/extension deficiency in surgical group was $2.68° \pm 2.98/2.04° \pm 2.88$, and in conservative group $3.22° \pm 3.65/1.85° \pm 2.66$. No significant difference was established. Using the Flynn criteria, the results were excellent and good in 58 (96.61 %) cases in the conservative group, and in 108 (99.08 %) cases in surgery group evaluating flexion deficiency and excellent and good in 59 (100 %) cases in conservative group, and in 107 (98.17 %) cases in surgery group evaluating extension deficiency. Humeroulnar (carrying) angle in A–P X-rays of elbow joint was evaluated according varus or valgus changes of the axis of traumatized arm. Average valgus displacement was $3.85° \pm 2.68$ in surgery group, and $4.95° \pm 3.14°$ in conservative group. No significant difference between those values was determined. Average varus displacement was $6.82° \pm 26.50$ in surgery group and $10.52° \pm 6.53$ in conservative group. Those values differ significantly ($p < 0.01$). Evaluating lateral humerocapitellar angle in sagittal X-rays, average extension displacement was $11.25° \pm 10.70°$ in surgery group and $13.47° \pm 10.67°$ in conservative group. No significant difference was established between those values. Average flexion displacement was $8.18° \pm 6.42°$ in surgery group and $12.67° \pm 11.83°$ in conservative group. Those values differ significantly ($p < 0.05$).
***Conclusions*** Statistically significant differences between groups were not obtained ($p > 0.05$), however radiological deviation of humeral head into flexion was greater in conservative group ($p < 0.05$) as well as the varus deviation ($p < 0.005$). Evaluating possible cosmetic deformation of traumatized arm, the results were excellent and good in 36 (61.02 %) cases of conservative group, and in 91 (84.26 %) in surgery group, while unsatisfactory respectively in 10 (16.95 %) and 6 (5.55 %). Frequency of neuropathies was significantly lower in conservative group.
***Significance*** In spite of the fact that the children's quality of life was better after conservative treatment, the cosmetic and functional results over a three year period was better in surgery group. We also need to keep in mind the iatrogenic nerve injury.

### EP79

### Tension Band Fixation for Pediatric Lateral Humeral Condyle Fractures: Novel Technique Using Absorbable Sutures Tightened by Double Loop Sliding Knot

**Kohji Kuriyama**, Ayako Uesugi, Toshiyuki Kataoka, Masayuki Hamada

Hoshigaoka Koseinenkin Hospital, Hirakata, Japan

**LEVEL 4/Trauma—Upper limb**
Keywords: Pediatric lateral humeral condyle fractures, Absorbable sutures, Tension band fixation.

***Purpose*** We have investigated a new tension band fixation technique using absorbable sutures for pediatric lateral humeral condyle fractures.
***Methods*** Eight skeletally immature patients were treated with open reduction and fixation with Kirschner wires and absorbable tension band suture for displaced pediatric lateral humeral condyle fractures. A double-loop sliding knot technique was used in tightening absorbable suture. The mean age of the patients was 5 years 2 months (range, 1 year 9 months—8 years 9 months). There was 1 girl and 7 boys. Postoperatively, a long arm cast was applied for 6 weeks. The Kirschner wires were removed in outpatient department at 6 weeks and range of motion exercise was begun. Fracture healing, malunion, growth disturbance, and presence of avascular necrosis were evaluated radiographically. Range of motion, and cosmetic deformity were recorded according to Flynn's criteria.
***Results*** The mean follow-up was 13.4 months (range 3.3–28.2 months). All the fractures healed satisfactorily without intraoperative or postoperative complications. There were no cases of malunion, growth disturbance or avascular necrosis. According to Flynn's criteria, functional results were excellent in five patients, good in two, and fair in one. Cosmetic results were excellent in seven patients, good in one.
***Conclusions*** The novel tension band fixation technique using absorbable sutures tightened by double loop sliding knot resulted in good outcomes.
***Significance*** This technique provides rigid initial fixation without a need for implant removal.

### EP80
### Our View of the Treatment of Sequelae After Monteggia Lesions in Childhood

**Jiri Jochymek**, Miroslav Straka

University Hospital Brno, Brno, Czech Republic

**LEVEL 4/Trauma—Upper limb**
Keywords: Chronic Monteggia lesion, Sequelae, Surgery reconstruction, Childhood.

***Purpose*** To review the current methods of operative management of post-traumatic radial head dislocation in chronic Monteggia lesions in children.
***Methods*** Post-traumatic chronic dislocation of the proximal radius usually occurs following a missed or ineffectively treated Monteggia fractures. The radial head is usually dislocated anteriorly. We performed open reduction of radial head, reconstruction of the annular ligament and corrective osteotomy of the ulna in eleven patients. Three of our patients underwent ulnar lengthening via gradual distraction using an Ilizarov external fixator. This procedure was used in nine boys and five girls who were treated for complications following Monteggia lesions between 2000 and 2009. The average age at the time of surgery was 8 years and 4 months, the average delay to surgery was 19 months, and a prerequisite for surgery was a normal concave articular surface of the proximal radius. The patients were followed up for an average of 28 months and, at final follow up, all were fully active and had no pain or instability.
***Results*** The complications included: non-union of the ulnar osteotomy site in two patients, residual radiocapitellar subluxation in two patients, and one patient had revision surgery. Nine patients showed a full range of motion, two had a loss of extension and three had a mean loss of pronation of 20° and a mean loss of supination of 20°. According to Letts, Locht and Wiens 57 % of the group had excellent results.
***Conclusions*** The operative treatment of consequences after Monteggia lesions in children has a relatively high rate of complications and unpredictable results. Indications for reconstruction include a normal articular surface of the radial head and normal alignment of the radius and ulna.
***Significance*** The Monteggia lesion is a rare fracture in childhood, but its sequelae can be serious. So far a unified therapeutic approach has not been proposed. Most authors prefer reconstruction surgery. Our results are in full agreement with the published data.

### EP81
### Distraction Osteogenesis Using External Fixators in Managing Residual Leg Length Discrepancy in Post-Malignant Bone Tumour Salvage Procedures

**Kwang-Won Park**, Hae-Ryong Song

Korea University Rare Diseases Institute, Seoul, Korea

**LEVEL 4/Tumors and metabolic disorders**
Keywords: Distraction osteogenesis, External fixator, Leg length discrepancy, Tumour salvage.

***Purpose*** Residual leg length discrepancy (LLD) is a common complication of limb-salvage techniques employed in the management of malignant musculoskeletal tumours. In this report, we present the use of distraction osteogenesis in the treatment of residual LLD in patients who had previously undergone limb salvage surgeries for osteosarcoma and the postoperative complications, as well as the advantages and disadvantages of this method.
***Methods*** A total of 5 patients who had undergone limb salvage surgery for malignant bone tumour and who were referred to our institute for treatment of subsequent LLD, were reviewed for this study. All 5 patients underwent limb lengthening, using either the monolateral external fixator system or the Ilizarov apparatus between 2004 and 2012. The mean age of the patients was 14.4 years (range 10–23 years) at the time of surgery. The mean LLD was 6.5 cm (range 5.3–8.6 cm) and the mean follow-up time after surgery was 2.4 years (range 0.9–5.4 years).
***Results*** The planned lengthening was achieved in all patients except 1. The mean amount of lengthening was 6.3 cm (range 5.6–7.8 cm). The mean fixation time was 8.5 months (range 5.5–12.5 months), and the mean external fixator index was 1.4 months/cm (range 0.9–2.2 months/cm). The postoperative complications included superficial pin track infection, equinus deformity, hardware failure, premature consolidation, re-fracture and prosthetic dislocation. The mean pixel value ratio (PVR) at the time of external fixator removal was 1.2. All calluses showed a cylindrical pattern with intermediate densities and were mostly homogenous.
***Conclusions*** The use of distraction osteogenesis with an external fixator has potential benefits in the treatment of residual LLD in patients who have previously undergone limb salvage surgeries for malignant bone tumours. The regeneration, consolidation and expected complications of this procedure are comparable with those of lengthening of normal bones.
***Significance*** Distraction osteogenesis with an external fixator may be recommended in the patients with residual LLD who have previously undergone limb salvage surgeries for malignant bone tumours.

### EP82

### Characterisation of Lower Limb Alignment Deformity in Children With Mucopolysaccharidosis Type I and Treatment With Guided Growth

**Elizabeth Ashby**, Markus Baker, Deborah Eastwood

Great Ormond Street Hospital for Children, London, UK

**LEVEL 4/Tumors and metabolic disorders**
Keywords: Mucopolysaccharidosis, Deformity, Guided growth.

***Purpose*** To describe the type of lower limb deformity seen in children with mucopolysaccharidosis (MPS) type I and describe its natural progression. We report the outcome of treating these deformities with guided growth.
***Methods*** This is a retrospective case series report of lower limb alignment in children with MPS type I at a teaching hospital from 2004 to 2013. Standardised full-length standing leg x-ray films were examined to determine the tibio-femoral angle. Children had an average of 6 x-rays with an interval of 12–24 months between images.
22 patients had full-length standing leg x-rays at the age of 8. Average tibio-femoral angle and range are reported at this age to provide a 'snap-shot' of limb deformity. These patients had a minimum of 3 x-rays performed over a period of 5 years. This allowed analysis of disease progression over time.
12 of the above 22 patients had their deformity treated with guided growth in the form of 8-plate insertion. We report the average time taken for the deformity to correct and the outcome when the 8-plates were removed.
***Results*** At the age of 8 years, the average tibio-femoral angle was 18° of valgus (range 4–34° valgus). When analysing knee deformity over a period of 5 years, 3 legs showed spontaneous improvement in alignment, 24 legs remained relatively stable and 17 legs developed a gradually worsening deformity.
12 patients were treated with guided growth and this was initially successful in all cases. The average deformity at the start of treatment was 26° valgus. 6 patients still have the 8-plates in situ and 6 patients have had the plates removed. The average tibio-femoral angle at the time of 8-plate removal was 4° of valgus (normal alignment). The plates remained in situ for an average of 22 months. Since removal 4 patients have developed a recurrence of their leg deformity almost equal to pre-surgery levels.
***Conclusions*** Children with MPS type I have valgus knee deformity. Approximately half of these deformities worsen with time and half remain stable. Treatment with guided growth is initially successful and results in normal knee alignment. When 8-plates are removed, the deformity commonly recurs. Further investigation is needed to assess if leaving the 8-plates in longer (allowing over-correction) would lead to a longer-lasting deformity correction.
***Significance*** Children with MPS type 1 commonly have a valgus deformity of the lower limbs. This can be successfully treated using guided growth in the form of 8-plate insertion. Once the 8-plates are removed, the deformity can recur. Further studies are needed to assess if initial over-correction of the deformity with 8-plates could lead to a more permanent result.

### EP83

### Radiographic and Clinical Skeletal Changes Following Bone Marrow Transplantation in Children With Osteopetrosis

**Dan Izrael**, Polina Stepensky, Amnon Khen, Naum Simanovsky, Vladimir Goldman, Ron Lamdan

Hadassah Hospital, Hebrew University, Jerusalem, Israel

**LEVEL 3/Tumors and metabolic disorders**
Keywords: Osteopetrosis, Bone marrow transplantation, Remodeling.

***Purpose*** Bone marrow transplantation has become the treatment of choice for children with osteopetrosis. Previously, affected children typically had "marble bones", suffered multiple frequent long bone fractures and exhibited diminished life expectancy. With current transplantation protocols survival rates are dramatically improved. However, little is known regarding the clinical behaviour and skeletal radiographic changes after transplantation. We describe the clinical and radiographic course after bone marrow transplantation in children with osteopetrosis
***Methods*** Medical records of 12 children with osteopetrosis who underwent bone marrow transplantation in our institution were reviewed. Type of genetic mutation, height, weight, blood count and electrolytes, orthopaedic manifestations and incidence of fractures were recorded. Radiographs were reviewed and measured.
***Results*** All 12 patients survived during an average follow up of 2.6 (range 1–5) years after transplantation. Gradual improvement in height and weight was noted. Hemoglobin level increased from 9.88 to 12.02 g% at 6 months and plateaud at 12.7 g% after 1 year. Initial radiographic changes were first noted 2–3 months after transplantation. However, contrary to a previous report, complete normalization of radiographs occurred only after 2–3 years with early constitution of a medullary canal and only later remodelling of the bone to its normal shape.
***Conclusions*** Bone marrow transplantation for osteopetrosis results in a slow ordered process of radiographic normalization of the skull and long bones lasting 2–3 years. During this period no increase in fracture incidence was noted.
***Significance*** This study adds to our knowledge regarding sequence of events, order and rate of the remodelling process after bone marrow transplantation for osteopetrosis.

### EP84

### An Evaluation of Forearm Deformities in Multiple Hereditary Exostoses: Modified Classification of Masada and Factors Associated with Radial Head Dislocation

**Ah Reum Jo**, Sung Taek Jung, Jae Young Moon, Hyun-Kee Yang

Chonnam National University Hospital, Gwangju, Korea

**LEVEL 3/Tumors and metabolic disorders**
Keywords: Multiple hereditary exostoses, Forearm deformity, Masada classification, Radial head dislocation, Ulnar shortening.

***Purpose*** Forearm and wrist are the most common sites of deformity in patients with multiple hereditary exostoses which include relative shortening of the ulna; bowing of the radius; ulnar deviation of the distal aspect of the radius, wrist and hand and dislocation or subluxation of the radial head. From our experience, when categorizing forearm deformities with Masada classification, a few cases did not fit the classification system well. It was not sufficient to estimate the progression of the deformity.
The purpose of the present study was to evaluate a series of patients who did not follow the previous classification in order to ascertain new combined categories and find out the indicator to predict the extent of deformities through the radiographic variables, such as radial bowing, ulnar shortening, ulnar variance, radioarticular angle and comparison of the clinical and radiologic outcomes.
***Methods*** We retrospectively reviewed 72 upper limbs of 36 paediatric MHE patients treated in our hospital from 2004 to 2012. The demographics, site of forearm involvement, treatments, and clinical and radiographic outcomes were reviewed. Proportional radial bowing calculated by a ratio of the distance from bicipital tuberosity to a maximal convex point of radius to radial length. Proportional ulnar shortening was calculated by a ratio of ulnar length to radial length.
***Results*** According to the Masada classification, three limbs were normal, 6 limbs were I, 2 limbs were II and 18 limbs were III. Using modified Masada classification, we classified the remaining 43 limbs as combined cases. We subdivided the combined cases into 3 groups. 35 limbs were I + III, 7 limbs were II + III, and 1 limb was I + II + III. Nine of 72 limbs (12.5 %) had a dislocation of radial head. From radiographic analysis, we found that the proportional ulnar shortening can cause a dislocation of radial head. The patients with proportional ulnar shortening less than 0.9 have a 7-times higher risk than those with more than 0.9. Analysis on mass location revealed that isolated distal ulnar mass has a tendency to increase dislocation of radial head. In addition, we discovered a correlation between ulnar variance and extent of radial bowing, with higher ulnar variance having more radial bowing.
***Conclusions*** We suggest that the patients with increased ulnar variance and significant ulnar shortening may deserve early surgery to decrease the progression of deformities especially radial head dislocation and radial bowing, contributing to a better forearm function.
***Significance*** Forearm deformities such as radial head dislocation are harmful for patients with multiple hereditary exostoses. With our indicators such as proportional ulnar shortening and ulnar variance, proportional radial bowing, we can reverse or decrease the progression of deformities especially radial head dislocation and radial bowing, contributing to better forearm function.

### EP85

### Genetic Study in Madelung's Deformity in Children

**Sandrine Huguet**, Florence Muller, Madeleine Aslan, Pierre Journeau

Children Hospital, Vandoeuvre, France

**LEVEL 3/Upper extremity**
Keywords: Gene mutation, Madelung's deformity, Children.

***Purpose*** Madelung's disease is a bone dysplasia predominantly seen in adolescent females, characterised by an epiphysiodesis of the medial and distal part of the radius. It includes a ventral and ulnar deformity of the radius, associated with a dorsal dislocation of the distal ulna. Madelung's deformity is known to be part of Leri-Weill disease (SHOX gene mutations) and is considered otherwise as an isolated anomaly. Our hypothesis is that the Madelung deformity is not found in isolation and is always part of some genetic modifications.
***Methods*** Our series includes 11 patients with bilateral deformities. Mean age was 9.3 years (8–13). We asked for the presence of wrist pain, and checked for patient's height, range of wrist motion and grip strength (JAMAR device). Xrays of the wrists were analysed. All patients had a genetic screening in keeping with the ethical recommendations of Helsinki (2000).
***Results*** Average wrist pain was 3/10. On clinical examination, all patients showed a small height (121–147 cm, minus 2 SD on average) and a decrease in all ranges of motion. The radiological values were all modified. All genetic screenings were positive: 2 for Turner syndromes, and 9 for SHOX gene mutations.
***Conclusions*** Limitations in range of motion are due to the vertical radial distal slope and to the dislocation of the distal ulna, whilst decreased grip strength seems to be due to the same reasons. Pain does not seem to be correlated to the degree of deformity. In our series Madelung's deformity was always associated with a genetic mutation, thus we recommend genetic screening when Madelung's deformity is observed.
***Significance*** Wrist pain in Madelung's disease is not correlated with the severity of wrist deformity. A Madelung deformity is associated with Leri-Weill disease (SHOX gene mutation) or Turner Syndrome (X0).

### EP86

### Prevalence of Carpal Coalition in the Pediatric Population

**Blazej A Pruszczynski**[1], Laurens Holmes Jr.[2], Kenneth J. Rogers[2], Jennifer M. Ty[2]

1. Department of Orthopaedics and Paediatric Orthopaedics, Lodz, Poland; 2. Department of Orthopaedics, Nemours/Alfred I. DuPont Hospital for Children, Wilmington, USA

**LEVEL 2/Upper extremity**
Keywords: Carpal coalition, Pediatric wrist, Congenital malformation.

***Purpose*** Carpal coalitions (CC) result from the failure of segmentation of the carpal anlage in early development. In the adult population, CC is usually described as an asymptomatic radiographic finding. Data on CC in the pediatric population are limited. This current study aims to examine the prevalence of CC in the pediatric population, and to characterize the presentation and associated musculoskeletal conditions.
***Methods*** We used a cross-sectional design to assess data collected from an estimated 1.5 million pediatric patients seen from August 2000 through February 2013 in our institution and associated facilities. A natural language software recognition (NLSR) program for key words was used. Ninety-four patients with CC were identified. Period prevalence, demographic and radiographic data were estimated using frequency and percentages.
***Results*** The period prevalence of carpal coalition in this pediatric population was 6:100,000. The frequency between sexes was comparable. Carpal coalitions were more prevalent among African American children. The initial age of presentation of the radiologic appearance of the CC varies between types. The most common CC (luno-triquetral, 69.5 %) had a mean age of presentation of 12 y10 m (SD 2 y7 m, 7 y2 m–19y6 m). The second most frequent CC (capito-hamate 18.5 %) had a mean age of presentation of 8 y6 m (SD 3 y7 m, 1 y0 m–16 y7 m). Carpal coalition was symptomatic in two patients. Thirty-eight patients had associated conditions such as skeletal dysplasia and limb deformity and most of these patients were diagnosed with capito-hamate coalitions.
***Conclusions*** The period prevalence of carpal coalition in this pediatric population is 6:100,000. This is lower than that previously reported. While the prevalence of CC is comparable by sex, it is most common among African American children. The most common CC is luno-triquetral followed by capito-hamate. Capito-hamate coalitions are more common among patients with associated conditions and can be diagnosed at a younger age relative to other subtypes in our group.
***Significance*** This study describes the period prevalence of carpal coalition in the pediatric population and highlights differences between types of CC and associated conditions such as skeletal dysplasia. A natural language software recognition program was used to search an electronic medical database of an estimated 1.5 million patients for information which had not specifically been recorded or coded as a diagnosis or problem. This suggests the reliability of NLSR in data capturing and sorting.

### EP87
### Influence of Hand-Dominance on Grip Strength in Children and Adolescents

**Ann Marjolein Hepping**, Joris Jan Willem Ploegmakers, Joannes Henricus Bernardus Geertzen, Sjoerd Klaas Bulstra, Martin Stevens

University Medical Center Groningen, Groningen, The Netherlands

**LEVEL 2/Upper extremity**
Keywords: Grip strength, Children, JAMAR hand dynamometer, Hand-dominance.

***Purpose*** In adults the dominant hand is often regarded to be 10 % stronger than the non-dominant hand. However, what is the influence of hand-dominance on grip strength in children and adolescents: are there differences between children of a different hand-dominance or gender?
***Methods*** Prospective cross-sectional study including children and adolescents aged 4–17 years. Participants were recruited from schools in the northern part of the Netherlands. All children were allowed a total of four attempts using the JAMAR hand dynamometer: twice with each hand. All individual attempts were scored. Hand-dominance was determined by asking what hand was used to write, or in case of 4- and 5-year olds when drawing a shape.
***Results*** The study population comprised 2284 children and adolescents. Right-dominant boys and girls scored significantly higher with their dominant hand, the difference amounting to 9.5 and 10.1 % respectively. Left-dominant girls scored significantly higher with their dominant hand, but this difference was only 3 %. For left-dominant boys no difference was found in favour of either hand. Left-dominant children more often score equal or higher with the non-dominant hand. Finally, there are trends for the dominant group to score higher on that particular side than the non-dominant group, but overall neither right-dominant boys nor girls were significantly stronger than their left-dominant peers, nor the other way around.
***Conclusions*** The 10 %-rule of the dominant hand regarding grip strength in adults also holds true for right-dominant children, but cannot be generalized for the left-dominant population. Left-dominant girls are significantly stronger with their dominant hand, but the effect is less evident amounting to only 3 %. For left dominant-boys no significant difference in strength in favor of either hand was found.
***Significance*** To our knowledge this is the first study that challenges the 10 %-rule of the dominant hand in children. It provides new insights regarding the influence of hand-dominance on grip strength in both left- and right-dominant boys and girls.

### EP88
### Outcome of Single Event Multilevel Surgery of Upper Limb (SEMLS) in Cerebral Palsy—Patients Perspective

Chakravarthy U Dussa

Orthopaedische Kinderklinik, Aschau, Germany

**LEVEL 2/Upper extremity.**
Keywords: Cerebral palsy, Upper Limb, Outcome.

***Purpose*** The purpose of the study is to assess patients/caretakers perception of functional improvement of the hand following SEMLS of upper limb.
***Methods*** A prospective study was conducted on 25 patients (27 limbs) with cerebral palsy who underwent single event multilevel surgery (SEMLS) of the upper limb. Apart from demographics, the types and levels of surgery and complications were documented. Minimum follow up time was 4 years.
The function of the upper limb was classified using MACS both pre and postoperatively. A patient/caretaker questionnaire was designed to document the hand function in his/her natural environment. The hand function preoperatively as well as at last follow-up and satisfaction was documented. The perception of improvement was documented in activities involving 5 areas of daily living. In addition patients/caretakers perception of improvement of elbow, wrist and thumb position was also documented. Other perceived areas of improvement were documented.
***Results*** M:F is 8:17. 27 limbs were operated on in 25 patients. 4 limbs in 4 patients were excluded due to lack of follow up. Mean age at the time of operation was 17.9 years. The procedures included muscle releases, transfers, capsulotomy, arthrodesis, and soft-tissue flaps. 93 % reported improvement in elbow, wrist and thumb position following surgery. At follow-up a significant improvement was seen in activities involving holding and gripping. Complex activities involving precision grip, turning and eating showed minimal to no improvement. However 44 % patients/caretakers felt in spite of visible improvement of the thumb position, it was still in the way of holding an object. Therefore, they preferred to choose a thumb abduction orthosis.

MACS score did not improve as expected in 40 % of our patients following SEMLS. Dividing these patients according to their age, 33 % of patients <15 years and 38 % >15 years did not improve in MACS following SEMLS. Relation to dominant side could not be measured. >80 % of the patients with no improvement reported that they would like to undergo the surgery again.

Complications included: temporary neuropraxia (2), supination deformity (1), recurrent elbow contracture (1) and thumb rearthrodesis (1).

***Conclusions*** SEMLS in upper limb corrects the deformities at all levels in a single sitting. Significant improvement was only noted in simple activities. Complex activities did not improve indicating lack of complex motor control. Suboptimal thumb correction inhibited the use of hand. Our questionnaire did not document the cosmetic aspect of the correction. However, this was evident from the satisfaction rate as well as patient reporting in the section 'other areas'.

***Significance*** SEMLS in upper limb addresses multiple problems at the same time. Even though the improvements following surgery are modest, they put the arm in a better position in space and optimize the already present function. Lack of improvement in function has little impact on patient satisfaction following surgery. Goal setting should be patient specific.

### EP89

### Surgical Correction of Congenital Radioulnar Synostosis: A Protocol for Treatment

**Tarek Hassan Abdelaziz**[1], Adel El-Sayyed[2]

Ain Shams University, Cairo, Egypt; 2. Children of Egypt's Hospital, Cairo, Egypt

**LEVEL 4/Upper extremity**

Keywords: Congenital radioulnar synostosis.

***Purpose*** Congenital radioulnar synostois (CRUS) is a rare anomaly that can be very disabling. It is more commonly bilateral than unilateral and the forearm is fixed in variable degrees of pronation. The decision to operate should be carefully planned in regard with the affected side, the dexterity of the child and the degree of the deformity. Surgical options include techniques that aim at restoring a range of pronation/supination through excising the mass, splitting the IOM and interposition of a graft and techniques that aim at placing the forearm in a new fixed but more functional position through a rotational osteotomy of the radius and/or ulna or through the synostosis mass. The results of techniques that attempt to restore a ROM are mostly disappointing and osteotomies through the radius and ulna are associated with a relatively high incidence of neurovascular (NV) complications. The decision to correct one/both hands in bilateral cases, when and whether to interfere in unilateral cases has also been a matter of debate. Accordingly we decided to set the following protocol for management of CRUS. Intervention should be around the age of four years. An osteotomy through the synostosis mass is performed. The non dominant hand should be shifted to a position of 20°–35° of supination. If the fixed pronation in the dominant hand is = 45° it is left alone, if >45° it is corrected to 30°. The aim is to allow eating and self care with the non dominant limb, while retaining the dominant limb for writing, keyboard activities and buttoning/unbuttoning clothes. The decision in bilateral/unilateral cases is taken accordingly. The aim of this study is to assess the results of our protocol including clear guidelines on when to intervene and performing an osteotomy through the synostosis mass.

***Methods*** Between August 2007 and July 2009, 20 forearms (12 patients) with CRUS underwent surgical correction through an osteotomy of the synostosis mass according to our protocol. The mean age at surgery was 6 years (4–8 y). Through a Boyd approach the synostosis mass was divided with an oscillating saw A 2 mm K-wire was introduced proximally into the olecranon and proximal ulna, the forearm was derotated into the desired position and then the K-wire was pushed distally into the radial shaft. A well moulded cast in the corrected position was applied for 6 weeks.

***Results*** The average duration of follow up was 5.2 years (4–6). The desired correction was achieved intraoperatively in all patients and bone union occurred at 6 weeks after surgery without any NV complications but there was a 10°–15° loss of correction in the cast in all forearms noted immediately after removal of the cast which did not increase over the years.

***Conclusions*** Correction of CRUS by an osteotomy through the synostosis mass is a safe, easy and efficient technique that can markedly improve the child's function and ability to perform activities of daily living. The clear guidelines on when to intervene and which forearm to correct help to clarify the confusion in the literature. Because of the loss of correction observed in all forearms we recommend overcorrection by 10° (more than in the protocol).

***Significance*** Level IV, case series study.
